# An undecacyclic carbon-centered hydrocarbon radical: synthesis and investigation

**DOI:** 10.3762/bjoc.22.95

**Published:** 2026-08-25

**Authors:** Ali S Acan, Johannes Werner, Joachim Podlech

**Affiliations:** 1 Institute of Organic Chemistry, Karlsruhe Institute of Technology (KIT), Kaiserstraße 12, 76131 Karlsruhe, Germany; 2 Institute of Inorganic Chemistry, Karlsruhe Institute of Technology (KIT), Kaiserstraße 12, 76131 Karlsruhe, Germany

**Keywords:** aromaticity, cyclopenta-fused polyaromatic hydrocarbons, DFT calculations, radicals

## Abstract

A stable, cyclopenta-fused polyaromatic hydrocarbon (CP-PAH) radical, comprising six six-membered and five five-membered rings alternately fused within an undecacyclic framework, was synthesized via *ortho*-fusion of a suitably *o,o*’-substituted difluorenylfluorene, followed by establishment of the π-conjugated system. The target radical was obtained in eight consecutive synthetic steps with an overall yield of 7%, with a Suzuki coupling and cyclization via electrophilic aromatic substitution (S_E_Ar) serving as key steps. Mesityl substituents attached to the five-membered rings provide kinetic stabilization of the radical species. Comprehensive characterization was performed using EPR spectroscopy, UV–vis spectroscopy, and cyclic voltammetry, further supported by quantum-chemical calculations. The computational studies enabled simulation of the UV–vis spectra and provided insights into spin-density distribution, aromaticity, and frontier orbital energies. The radical is best described by the resonance structure in which the unpaired electron is predominantly localized on the outer cyclopentadiene units, maximizing the number of preserved aromatic benzene sextets. Calculations indicate only a minor triradical contribution to the electronic ground state. Notably, the five-membered rings, particularly the central ring, exhibits pronounced antiaromatic character.

## Introduction

**Figure 1:**
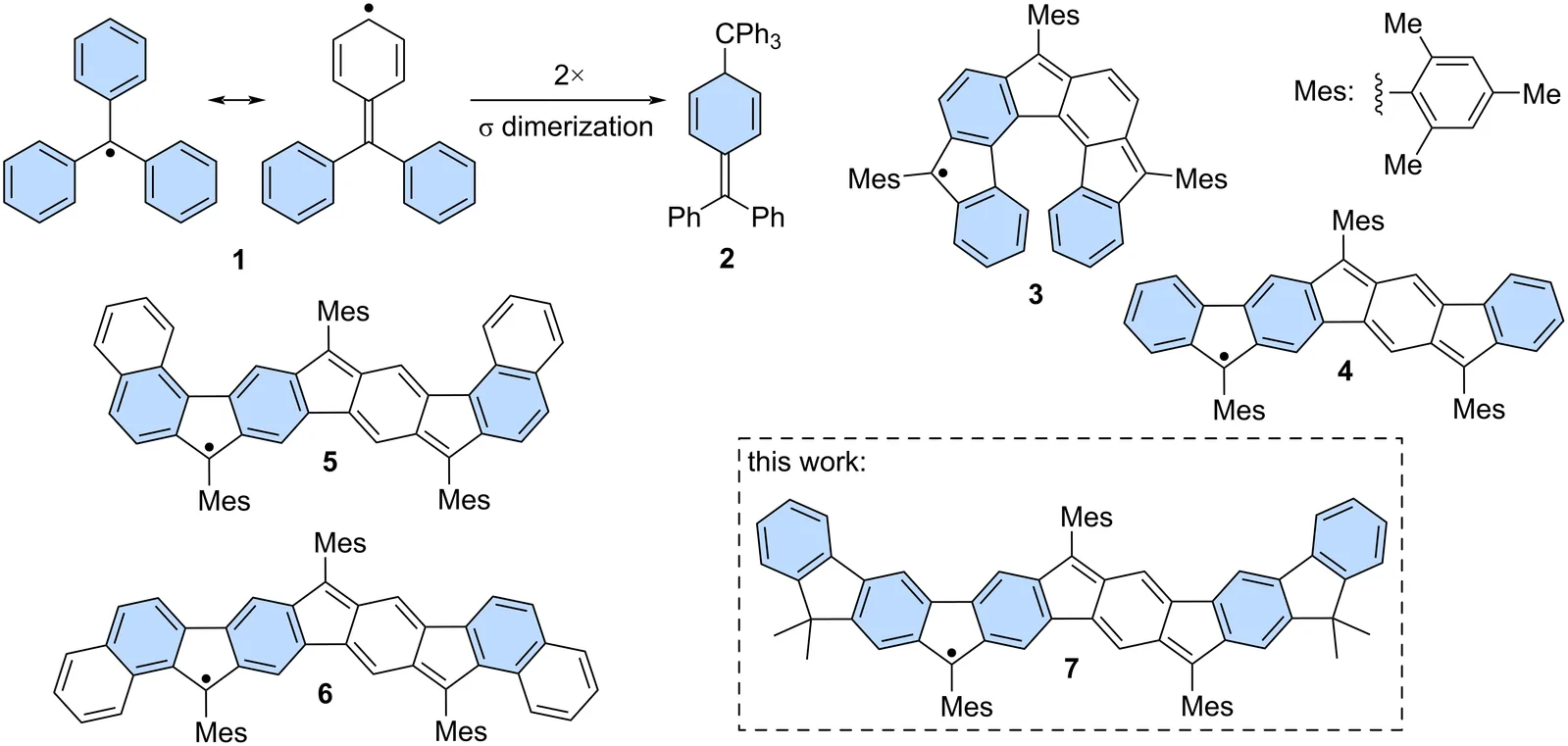
CP-PAH radicals synthesized in our group.

Polyaromatic hydrocarbons (PHs) [1–6] have found significant interest among the aromatic compounds due to their unique electronic properties, particularly the small gaps of their frontier orbitals. When five-membered rings are part of the polyaromatic systems [7–13], these are commonly referred to as cyclopenta-fused polyaromatic hydrocarbons (CP-PAHs or CPPAs) [14–17], as non-alternant conjugated polycyclic hydrocarbons (CPHs) [18–19], or are classified as compounds featuring quinoidal conjugated units. Since the first report of the triphenylmethyl radical **1** by Gomberg (Figure 1) [20–21], numerous stable carbon-centered hydrocarbon radicals [22] have been synthesized and investigated. However, only a limited number of these radicals turned out to be stable towards air, moisture, and solvents, or showed resistance to dimerization (as in the formation of Gomberg’s dimer **2**) or disproportionation. Enhanced radical stability is generally achieved through extended π-conjugation and/or the incorporation of bulky substituents to prevent dimerization [23–26].

These compounds are quite special due to their structural features correlated with their radical properties. These give rise to interesting electronic, optical, and magnetic properties and might thus be utilized in organic electronics [4] or in nonlinear optics [27]. They might further find application in spintronics [28–29], as organic magnets [30–32], or in fully organic spintronic devices [33]. Moreover, since the physical properties of hydrocarbon radicals primarily depend on the arrangement of the π electrons, the design and synthesis of novel hydrocarbon radicals remain essential for expanding the library of molecular scaffolds available for spintronic applications [24].

Aromaticity is a fundamental feature of fully conjugated polycyclic systems [34], where aromatic systems are easily identified by applying Hückel’s [4*n* + 2] π electron rule [35]. Dewar and Breslow later presented the [4*n*] π electron rule, which allowed for the easy identification of antiaromatic systems [36–38]. While these rules provide a useful framework for assessing aromaticity, they are of limited use for the differentiated characterization of polycyclic systems [39–40]. Furthermore, since aromaticity cannot be measured directly, both its definition and its practical significance have been a subject of controversy [41]. Nevertheless, due to the concept’s practicability, it is continuously applied in basic and even in sophisticated investigations [42]. Computational methods based on density functional theory (DFT) have meanwhile emerged as powerful supplementary tools to elucidate and quantify the aromatic and antiaromatic character of molecular systems. Here, the nucleus-independent chemical shift XY scan (NICS-XY-scan) has proven particularly useful for the evaluation of polycyclic compounds. It allows the visualization and analysis of ring-current effects by calculation of a molecule’s magnetic response to an external magnetic field [43].

We recently reported the synthesis of a series of radical systems based on indenofluorene (IF) cores, including a helical radical **3** [44] and its linearly arranged isomer **4** [45]. Building on these studies, we focused in further investigations on the expansion of the linear framework, where we synthesized nonacyclic radicals **5** and **6** [46].

Herein, we report the synthesis and comprehensive characterization of a new CP-PAH radical **7** as a further rare example of a stable, carbon-centered radical with a further extended π system.

## Results and Discussion

### Synthesis

The synthesis of radical **7** was initiated by preparation of the corresponding boronated building block **12**, followed by Suzuki coupling with a dibromofluorene building block **13**, and concluded by a double *ortho*-cyclization and subsequent rearomatization.

**Scheme 1:**
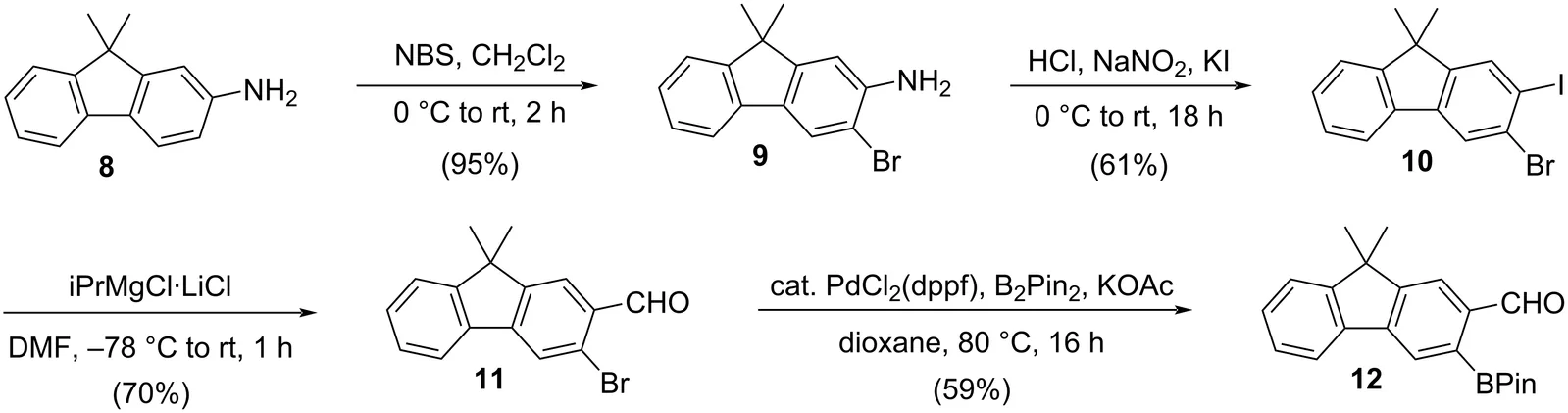
Synthesis of the nucleophilic outmost ring systems.

Pinacol (Pin) boronate **12** was synthesized from commercially available fluorene **8**. Initial bromination with *N*-bromosuccinimide (NBS) furnished the brominated intermediate **9**, which was subsequently converted into the corresponding iodinated derivative **10** in a Sandmeyer-type reaction (Scheme 1). Treatment of **10** with iPrMgCl·LiCl furnished aldehyde **11** and the synthesis was concluded by a Miyaura borylation to the boronated species **12**.

**Scheme 2:**
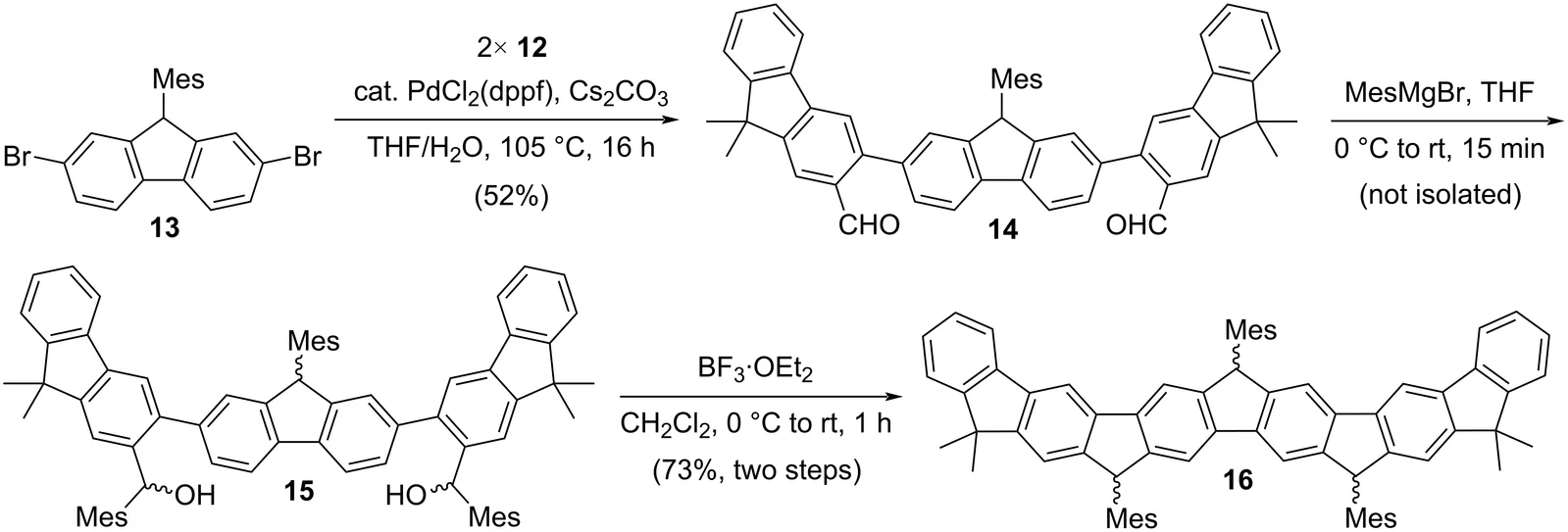
Suzuki coupling and establishment of the undecacyclic ring system.

The synthesis of mesityl-substituted fluorene **13** from commercially available 2,7-dibromo-9*H*-fluoren-9-one has already been reported [45]. Its subsequent double Suzuki coupling with boronate **12** generated terfluorene **14** (Scheme 2). The following nucleophilic addition with a mesityl Grignard reagent (MesMgBr) converted dicarbaldehyde **14** into the corresponding secondary dialcohol **15**. Subsequent treatment with boron trifluoride etherate furnished precursor **16** as a mixture of diastereoisomers, whose separation was neither expected easy to achieve nor necessary for the following step.

**Scheme 3:**
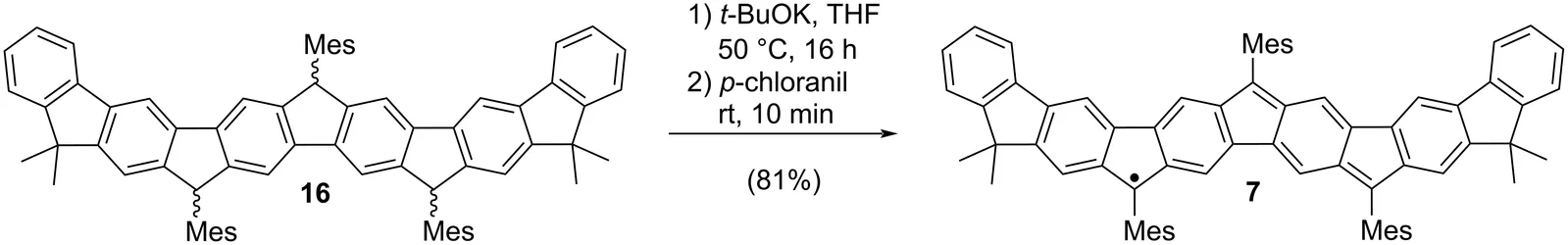
Finalization of the radical synthesis.

In a finalizing step, **16** (as the mixture of isomers) was deprotonated with *t*-BuOK and immediately oxidized with *p*-chloranil to yield radical **7** as a dark green solid (Scheme 3).

Compound **7** was thus synthesized from **8** in eight consecutive reaction steps with an overall yield of 7%. This radical displayed excellent stability under ambient conditions, showing resistance towards air, moisture, daylight, and dimerization. Furthermore, in its solid state and under an inert atmosphere, **7** proved to be stable for over a year.

**Figure 2:**
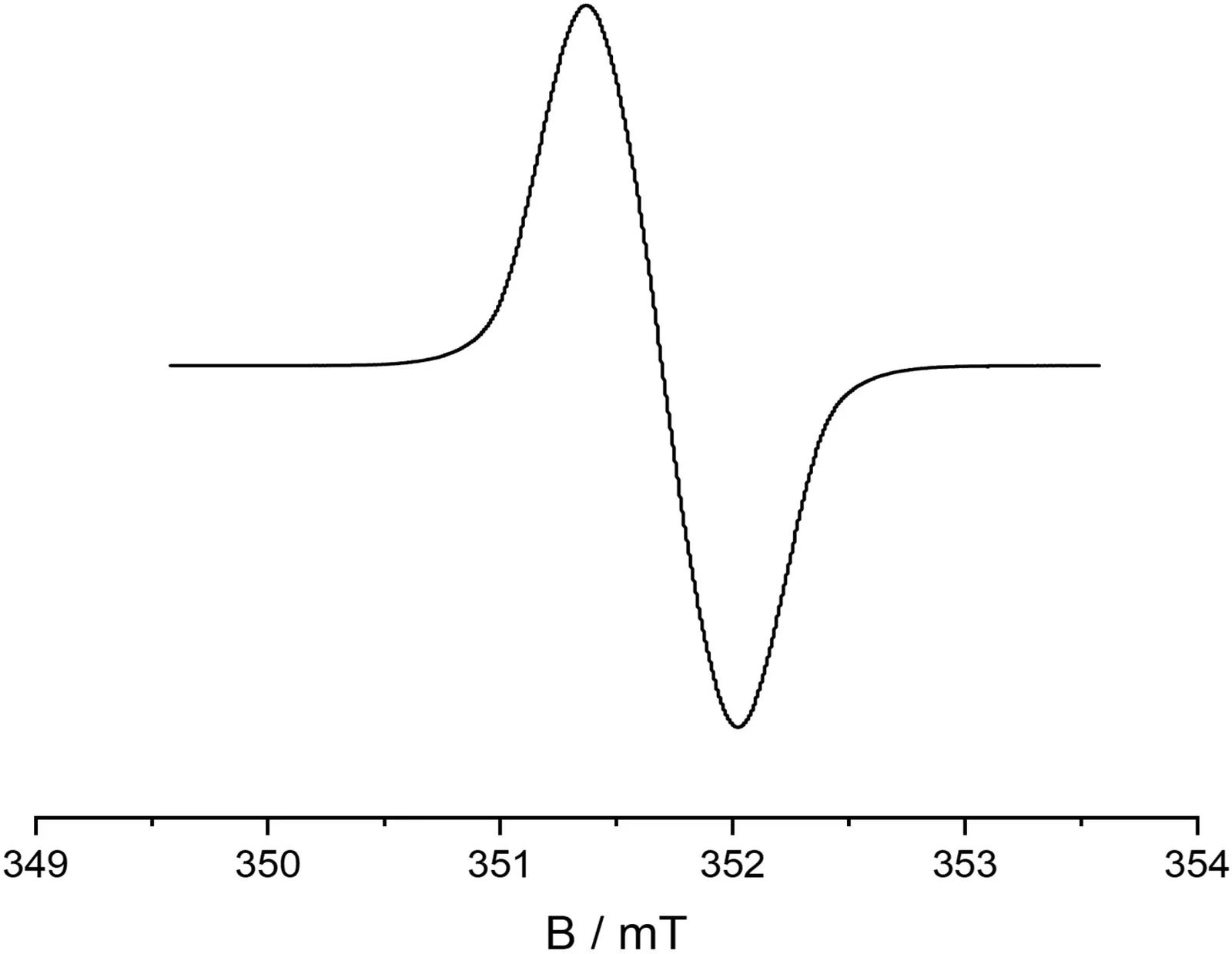
CW (continuous wave) X-band (9.4214 GHz) EPR spectrum of **7** in toluene (1 mM) at ambient temperature showing a single resonance signal centered at *g* = 2.0038.

The paramagnetic nature of radical **7** was demonstrated by the lack of observable resonances in conventional frequency ranges in the respective NMR spectra and by its response in EPR spectroscopy. The measured X-band EPR spectrum of **7** showed a single line centered around *g* = 2.0038 without any discernible hyperfine couplings (Figure 2).

**Figure 3:**
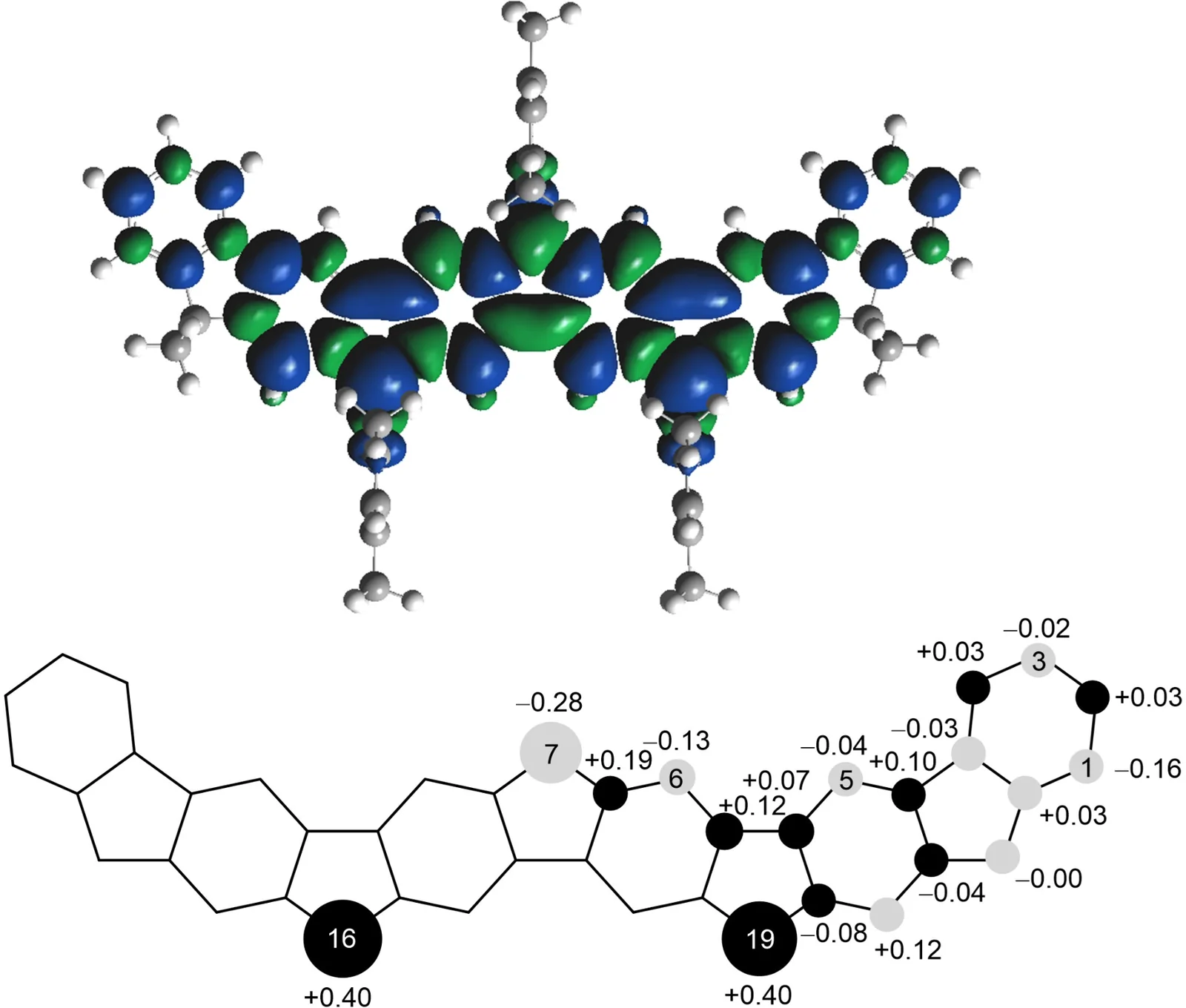
Calculated spin densities of **7**; blue: α spin, green: β spin (isovalue: 0.004 electrons per bohr; calculated at the upbe0/def2-TZVP/GD3BJ level).

**Scheme 4:**
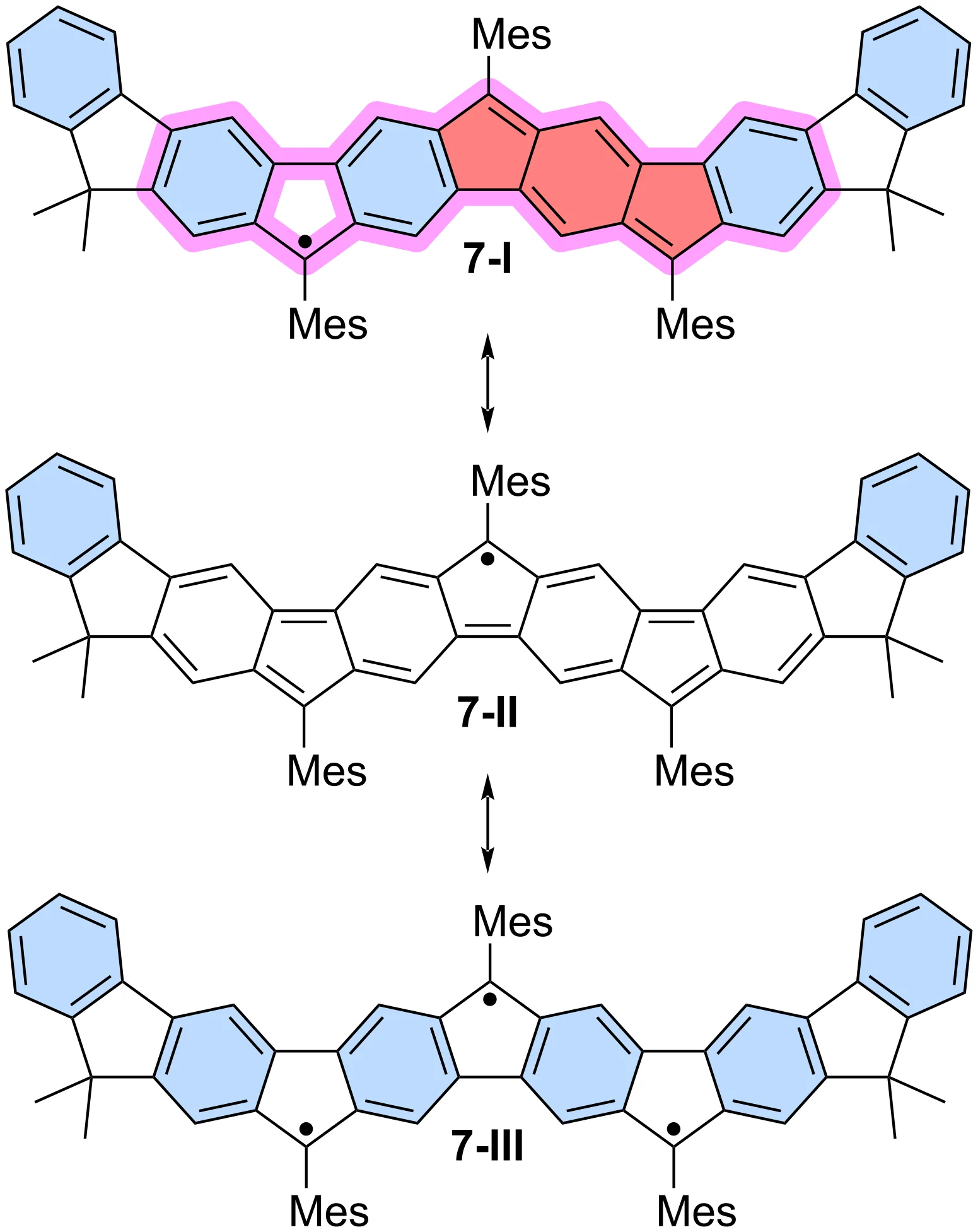
Resonance formulas of radical **7**. Fully intact benzene rings are highlighted in blue; the indacene unit is highlighted in red. The fully conjugated central core is marked in magenta.

The calculated EPR data indicate that the spin density is delocalized over the undecacylic framework, which accounts for the absence of resolved hyperfine structure. Nevertheless, pronounced Mulliken atomic spin densities (Figure 3) are calculated for the mesityl-substituted carbon atoms. Densities at the respective outer carbons C-16 and C-19 show the highest values of 0.40, respectively, whereas position C-7 takes a slightly smaller value of −0.28. Calculation of the natural orbital occupation numbers (NOONs) [47] and application of the Yamaguchi scheme [48–49] led to a *y* value of 0.26, indicating only marginal contribution of the species’ triradical character. Therefore, resonance structure **7-I** (cf. Scheme 4) and its mirror image should thus have the highest relevance in the ensemble of possible resonance structures, which is in good agreement with prior results from our group [45–46].

**Figure 4:**
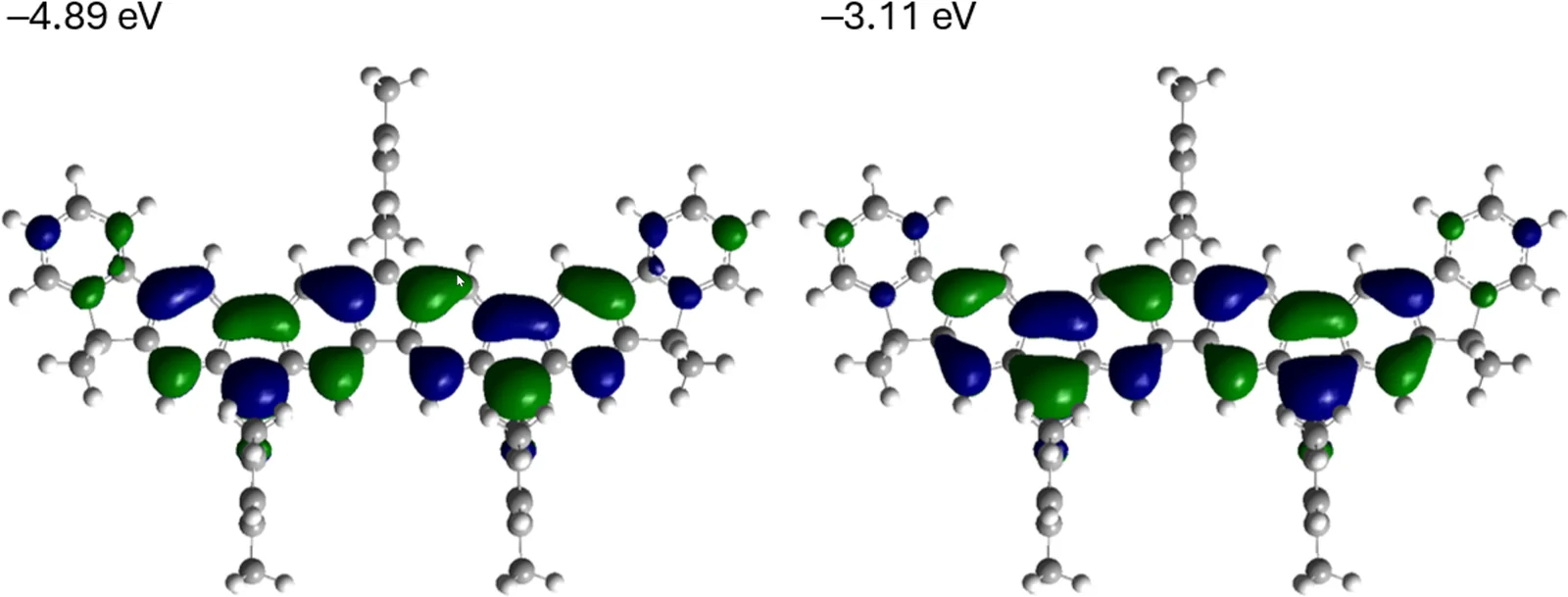
Calculated SOMOs of α (left) and β electrons (right) for radical **7** (also termed SOMO and SUMO in the literature [50]; isovalue: 0.02 electrons^1/2^ bohr^−3/2^; calculated at the upbe0/def2-TZVP/GD3BJ level).

The SOMOs of **7** (also termed SOMO and SUMO in the literature [50], Figure 4) feature a nodal plane through the center of the molecule; their energies are −4.89 and −3.11 eV, respectively, for the α and the β spin.

**Figure 5:**
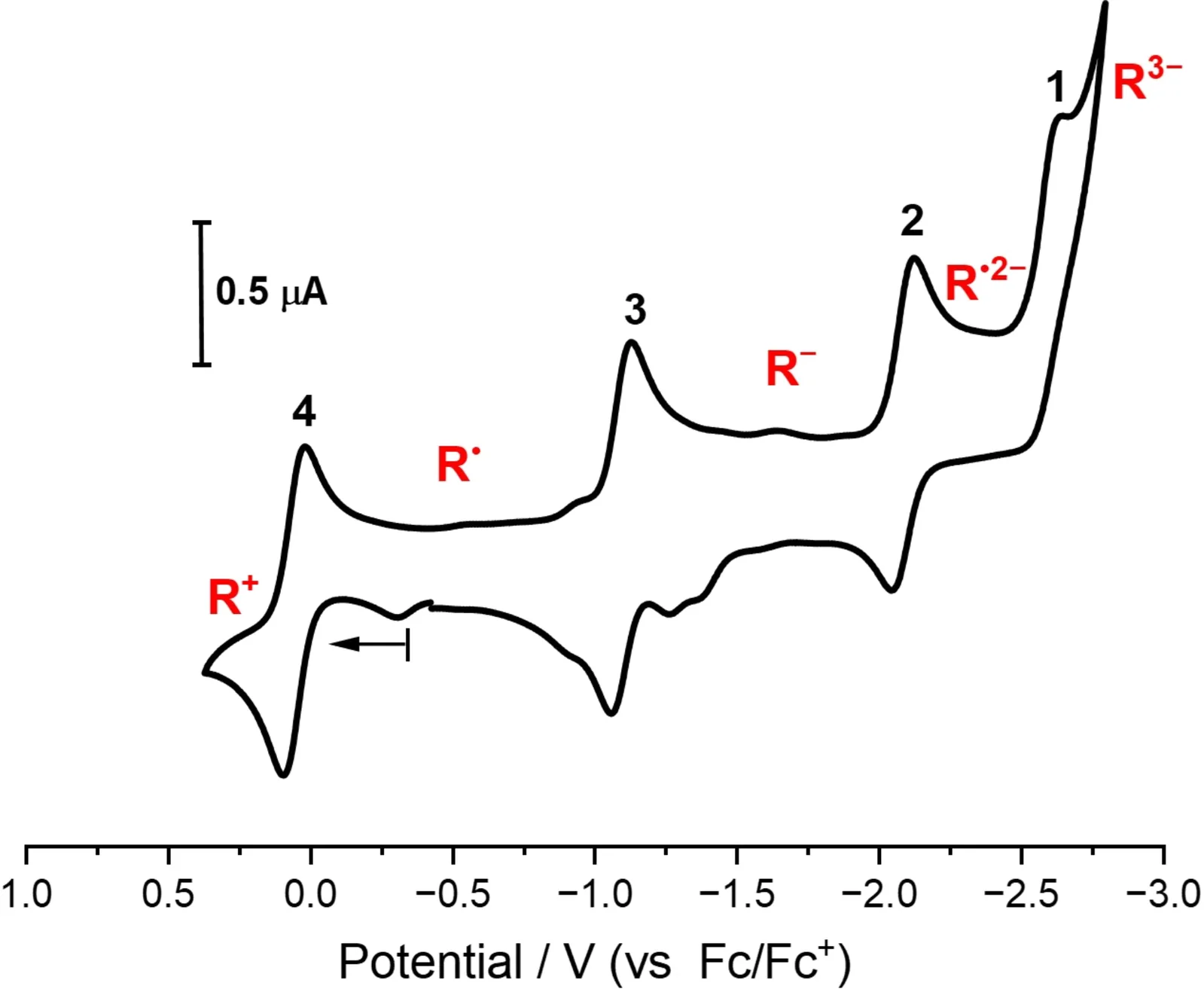
Cyclic voltammogram of **7** recorded in THF at room temperature (vs Fc/Fc^+^*, v* (THF) = 100 mV s^−1^; Pt/[NBu_4_][PF_6_]/Ag, internal standard: Fc*).

The electrochemical characteristics of **7** were evaluated by cyclic voltammetry, where the redox potentials were determined vs the ferrocene/ferrocenium couple, Fc/Fc^+^ using decamethylferrocene Fc* as internal standard (Figure 5) [51]. In tetrahydrofuran we observed one irreversible and three quasi-reversible redox processes centered at 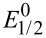 (THF) values of −2.58, −2.08, −1.09, and +0.06 V (vs Fc/Fc^+^). These values are well in line with the results from previous investigations in our group [45]. They allow the assignment of these potentials to specific redox processes, where the first three values indicate reductions to the tri-, radical di- and monoanion, respectively, and the last value most likely refers to the oxidation to the monocation [45].

**Figure 6:**
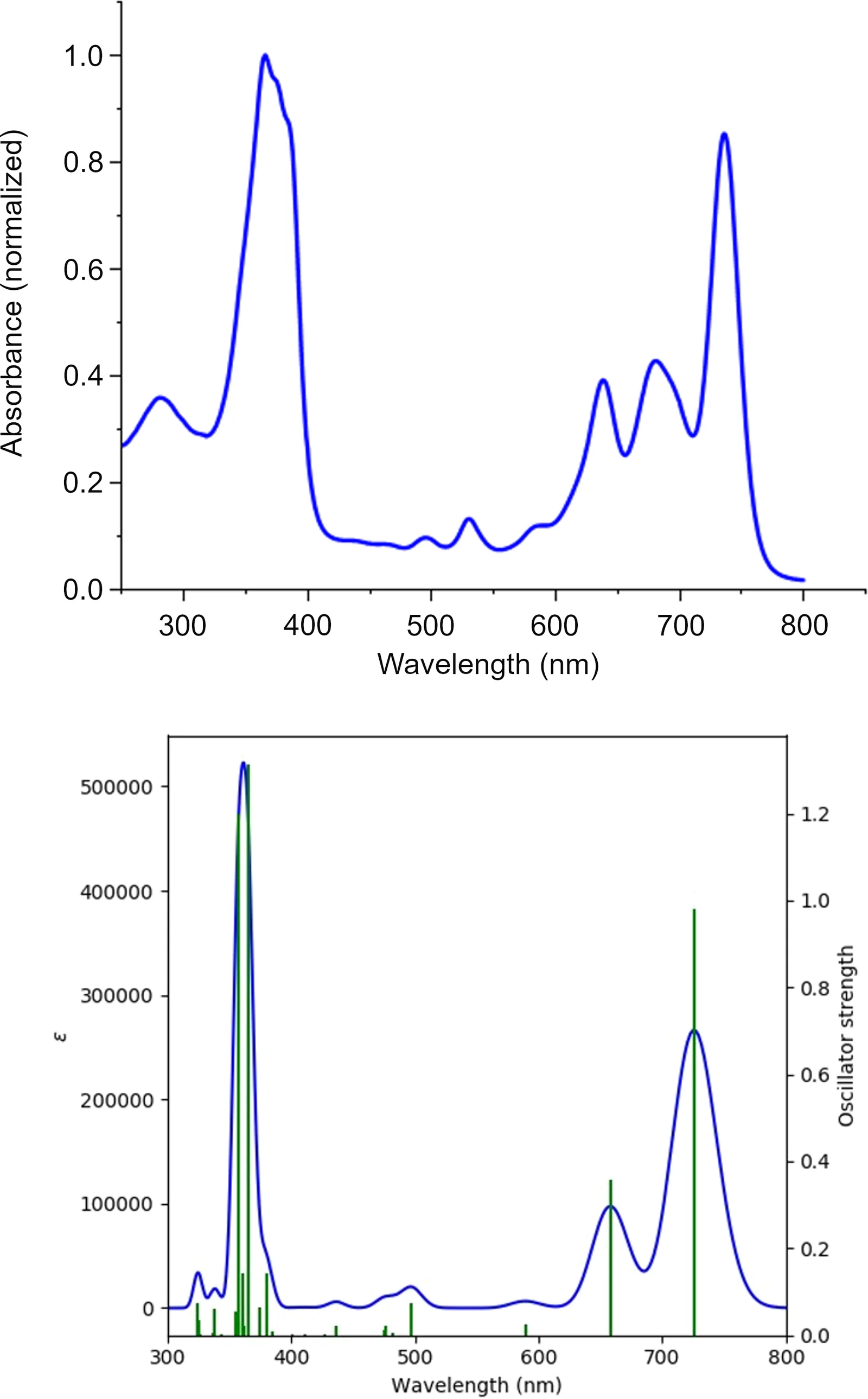
UV–vis spectrum of radical **7** in CH_2_Cl_2_: measured (top), calculated (bottom).

A UV–vis spectrum of radical **7** was recorded in CH_2_Cl_2_ (Figure 6, top). It displays a series of intense bands across a broad spectral range, extending from the ultraviolet into the visible region. The absorption spectrum is in good agreement with a calculated UV–vis spectrum of **7**, obtained by time-dependent (TD) DFT calculation [upbe0/def2tzvp/GD3BJ level] with a simulated solvent field of methylene chloride (Figure 6, bottom). A substantial peak with lowest energy is observed at 740 nm in the experimental spectrum. According to the TD calculation it is mostly due to a SOMO → LUMO transition. This low-energy transition is well in line with the relatively small negative reduction potential determined by cyclic voltammetry. Further significant absorptions in the visible range follow at 680 and 640 nm. The peak of greatest intensity has several shoulders and is observed between 320 and 410 nm with an absorption maximum at 367 nm. An enlarged and expanded version of Figure 6 is given in Supporting Information File 1 together with significant transitions. No fluorescence activity was observed for this compound, consistent with previous reports on related systems [44,46]. This is presumably attributable to the extremely short excited-state lifetimes reported for indenofluorenes, likely caused by rapid non-radiative decay via conical intersection [52].

Obviously, an aromatic or antiaromatic character is an outstanding feature of a fully conjugated cyclic or polycyclic system. Especially an antiaromatic contribution in these systems is often correlated with useful optoelectronic properties [34]. Aromaticity is most simply estimated by the rule of Clar [53–55], later extended by Glidewell and Lloyd [56], which describes the tendency of polyaromatic systems to maximize the number of fully aromatic benzene rings in their Lewis structures. This approach not only explains the relative thermodynamic stability of isomeric structures but also enables the identification of dominant resonance contributors within the resonance ensemble.

A detailed analysis of the possible resonance structures of radical **7** (Scheme 4) reveals clear correlations with previously described conclusions [45]. Among these, structures **7-I** and its mirror image (monoradical species) and **7-III** (triradical species) are particularly significant, preserving five and six intact benzene units, respectively. In contrast, structure **7-II** (monoradical species) only retains intact units at the terminal rings and should therefore be the least significant of the depicted resonance formulas. The terminal benzene rings do not significantly contribute to the electronic delocalization of the system, due to the interruption of conjugation by the dimethyl-substituted carbons in the outer cyclopentadiene units. Under this assumption, the presented compound would formally be equivalent to the linear heptacyclic system **4** (cf. Figure 1). In conclusion, the incorporation of building block **12** enabled the expansion of the molecular framework, while having only a limited effect on the intrinsic electronic properties of the radical system.

**Figure 7:**
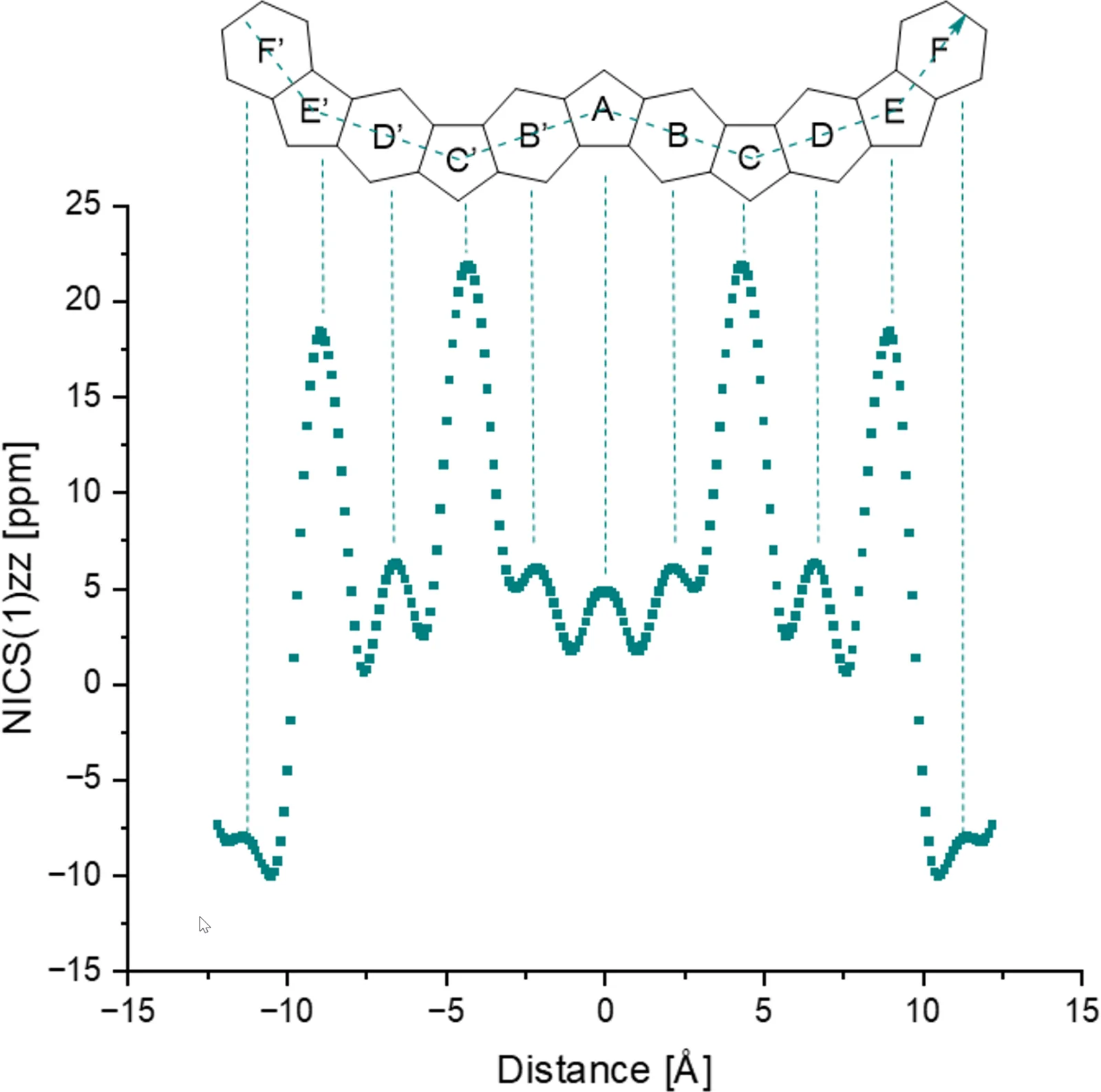
NICS(1)_πzz_-XY-scan of radical **7** (calculated without mesityl and methyl groups).

Aromatic properties are most conveniently investigated by computing NICS (nucleus-independent chemical shift) values to simulate a response of an aromatic system to an external magnetic field. These are commonly calculated at a 1 Å distance from the center of each ring [NICS_zz_(1.0) values] [43,57–60] and allow for a quantification of the local aromaticity of individual rings: Negative values indicate aromatic character and positive values correspond to antiaromaticity. NICS-XY-scans are constructed by calculation of NICS values (typically again at a 1 Å distance to the ring system) along a path through the core of the compound passing all ring centers. This approach is especially suitable for polyaromatic systems with condensed rings showing more than one ring current [61] and thus allows for a differentiated estimation of the aromaticity pattern in polycycles. The shielding behavior of the undecacyclic system was investigated by employing a NICS-XY-scan (Figure 7). The results indicate that all five-membered rings exhibit antiaromatic character, which is particularly pronounced in rings E’, C’, C, and E. The inner six-membered rings D’, B’, B, and D display a borderline behavior, as they are neither distinctly aromatic nor antiaromatic. This is likely a consequence of the antiaromatic indacene subunits embedded in the molecular framework (Scheme 4, highlighted in red) within the framework [36–37,62], particularly of the C’, B’, A fragment and its mirror image. In contrast, pronounced aromatic character is observed exclusively in the outer six-membered rings (F and F’), whereas the remainder of the framework predominantly shows antiaromatic character. The outmost rings are not part of the fully conjugated core, but could be considered as phenyl substituents attached to rings D and D’, which accounts for the fully retained aromaticity.

**Figure 8:**
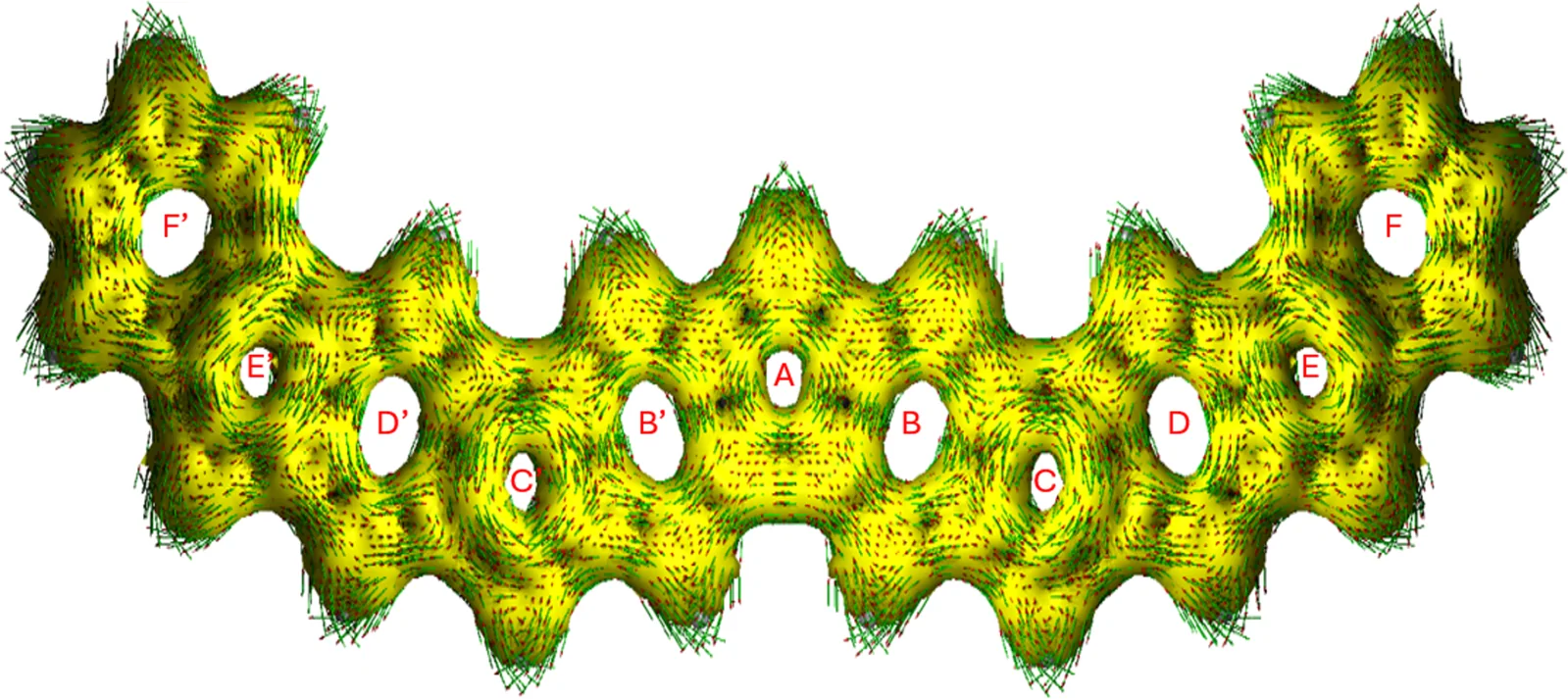
ACID isosurface plot of **7** (calculated without mesityl and methyl groups; isosurface value 0.02).

Aromaticity can further be quantified by the investigation of ring currents in the π system. The anisotropy of the induced current density can be calculated and visualized with Herges’ ACID (anisotropy of the induced current density) method (Figure 8) [63–64]. The results suggest antiaromatic behavior for the five-membered rings. For rings B’, B, D’, and D, again a combination of aromatic and antiaromatic character is observed, which is in agreement with the corresponding NICS-XY-scan. In contrast, clear aromatic behavior is exclusively observed in the terminal rings F’ and F.

## Conclusion

In conclusion, we have synthesized a π-extended open-shell CP-PAH composed of six benzene and five cyclopentadiene rings, representing a further example of a stable carbon-centered radical. The compound was obtained via cyclization of an *o,o’*-substituted difluorenylfluorene, followed by reestablishment of the conjugation. The radical is best described with the unpaired electron predominantly localized on the outer cyclopentadiene units, thereby maximizing the number of fully aromatic benzene rings. It exhibits vanishingly small triradical character as well as a small SOMO–LUMO gap. Overall, the synthesis of radical **7** provides a fundamental basis for further investigation of π-extended radical systems. It not only demonstrates the feasibility of accessing larger, structurally related species with notable stability, but also provides a valuable basis for the development of more complex and intriguing molecular systems.

## Supporting Information

File 1 Experimental procedures, NMR and further spectra for new compounds and details of DFT calculations.

## Data Availability

All data that supports the findings of this study is available in the published article and/or the supporting information of this article.
