# Supplementary material for: An undecacyclic carbon-centered hydrocarbon radical: synthesis and investigation
Source: Beilstein J Org Chem. 2026 Aug 25;22:1196–204. doi: 10.3762/bjoc.22.95 (PMC13550852; doi:10.3762/bjoc.22.95)
Supplement: Supplementary file 1 [file Beilstein_J_Org_Chem-22-1196-s001.pdf]

BEILSTEIN JOURNAL OF ORGANIC CHEMISTRY

Supporting Information

for

An undecacyclic carbon-centered hydrocarbon radical: synthesis and investigation

Ali S. Acan, Johannes Werner and Joachim Podlech

Beilstein J. Org. Chem. **2026,** *22,* 1196–1204. doi:10.3762/bjoc.22.95

Experimental procedures, NMR and further spectra for new compounds and details of DFT calculations

License and Terms: This is a supporting information file under the terms of the Creative Commons Attribution License (https://creativecommons.org/licenses/by/4.0). Please note that the reuse, redistribution and reproduction in particular requires that the author(s) and source are credited and that individual graphics may be subject to special legal provisions.
The license is subject to the *Beilstein Journal of Organic Chemistry* terms and conditions: (https://www.beilstein-journals.org/bjoc/terms)

Content

1. Experimental procedures S2
2. NMR spectra of all compounds and mass spectra S7
3. UV–vis spectrum S15
4. EPR spectra S16
5. Cyclic voltammetry (CV) S17
6. Computational details S18
7. Archive entry for a single-point energy calculation on a minimum structure of radical **7** S18
8. Frequency analyses and visualized IR spectra S20
9. SOMOs of α and β electrons of radical **7** S22
10. Calculated spin densities of radical **7** S23
11. TD calculations S24
12. Calculated UV–vis spectra of radical **7** S26
13. Calculated EPR data for radical **7** S27
14. Triradical character S30
15. NICS-XY-scan of radical **7** (calculated without methyl and mesityl groups) S31
16. ACID Plot of radical **7** (calculated without methyl and mesityl groups) S34
17. References S35

1. Experimental procedures

Compound **8** was obtained commercially and compound **13** was prepared following a published procedure [1]. Technical grade solvents (CH_2Cl_2, *n*-hexane, and *n*-pentane) were distilled prior to use. THF was dried over sodium, CH_2Cl_2 was dried over CaH_2, and both were distilled prior to use. Anhydrous toluene (99.8%) was commercially obtained and used without further purification. Flash column chromatography was performed using Merck SiO_2 60 (230–400 mesh), thin-layer chromatography was performed on commercially available Merck F_{254} pre-coated sheets. ^1H and ^{13}C NMR spectra were recorded on a Bruker Avance 400 spectrometer. Chemical shifts are given in ppm and are referenced by using the residual solvent signals as internal standards [2]. IR spectra were recorded on a Bruker Alpha FT-IR spectrometer using ATR technique and mass spectra were recorded with a Finnigan MAT-95 mass spectrometer and a Thermo Fisher Scientific Q Exactive Orbitrap mass spectrometer. CW-EPR spectra were recorded on a Bruker EMXplus X-band spectrometer (microwave frequency: 9.42 GHz) and were calibrated against DPPH (g = 2.0036) [3]. The spectra were simulated using the MATLAB/EasySpin software package [4].

3-Bromo-9,9-dimethyl-9*H*-fluoren-2-amine (9)

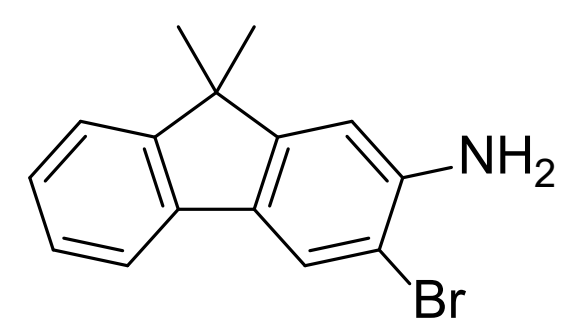

Following a published procedure [5], NBS (893 mg, 5.02 mmol, 1.05 equiv) was added portionwise under an argon atmosphere to a cooled solution (0 °C) of 9,9-dimethylfluoren-2-amine (**8**; 1.00 g, 4.78 mmol, 1.00 equiv) in 40 mL CH_2Cl_2 and allowed to warm to rt. After 2 h, the mixture was quenched with $NaHCO_3$, the phases were separated and the aqueous phase was extracted with CH_2Cl_2. The combined organic layers were dried over Na_2SO_4, filtered, and concentrated under reduced pressure. The obtained residue was purified by column chromatography (*n*-hexane/EtOAc 9:1) to yield **9** (1.31 g, 4.56 mmol, 95%) as a beige solid. The obtained NMR data is in agreement with the literature [5].

R_f = 0.32 (*n*-hexane/EtOAc 9:1).

^1H NMR (400 MHz, $CDCl_3$): δ (ppm) 7.8 (s, 1 H, H-Ar), 7.5 (dt, J = 7.6, 0.9 Hz, 1 H, H-Ar), 7.4–7.3 (m, 1 H, H-Ar), 7.3 (td, J = 7.4, 1.3 Hz, 1 H, H-Ar), 7.2 (td, J = 7.4, 1.3 Hz, 1 H, H-Ar), 6.8 (s, 1 H, H-Ar), 4.2 (s, 2 H, NH_2), 1.4 (s, 6 H, CH_3).

3-Bromo-2-iodo-9,9-dimethyl-9*H*-fluorene (10)

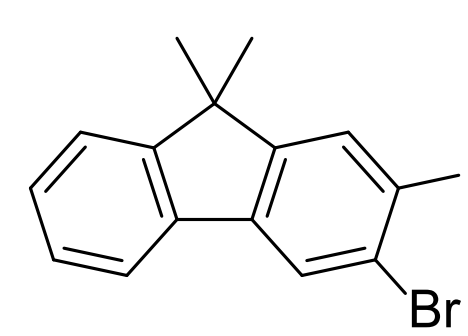

Following a published procedure [5], 3-bromo-9,9-dimethylfluoren-2-amine (**9**; 1.00 g, 3.47 mmol, 1.00 equiv) was suspended in conc. HCl (10 mL) at 0 °C and $NaNO_2$ (527 mg, 7.63 mmol, 2.20 equiv) in H_2O (10 mL) was added portionwise over a period of 10 min. The mixture was stirred vigorously for 2 h at 0 °C and subsequently KI (3.46 g, 20.8 mmol, 6.00 equiv) in H_2O (20 mL) was added at 0 °C and stirring continued overnight (18 h) while allowing the mixture to warm to rt. Solid sodium disulfite was added until the color vanished followed by the addition of CH_2Cl_2. The phases were separated and the aqueous phase was extracted with CH_2Cl_2. The combined organic layers were washed with saturated aqueous $NaHCO_3$ solution and brine, dried over Na_2SO_4, and concentrated under reduced pressure. The obtained crude was purified by column chromatography (silica gel, *n*-hexane/CH_2Cl_2 9:1) to yield **10** (844 mg, 2.12 mmol, 61%) as a white solid. The obtained NMR data is in agreement with the literature [5].

R_f = 0.50 (*n*-hexane/CH_2Cl_2 9:1).

^{1}H NMR (400 MHz, $CDCl_3$): δ (ppm) 8.0 (s, 1 H, H-Ar), 7.9 (s, 1 H, H-Ar), 7.7–7.6 (m, 1 H, H-Ar), 7.4–7.4 (m, 1 H, H-Ar), 7.4–7.3 (m, 2 H, H-Ar), 1.5 (s, 6 H, CH_3).

3-Bromo-9,9-dimethyl-9*H*-fluorene-2-carbaldehyde (11)

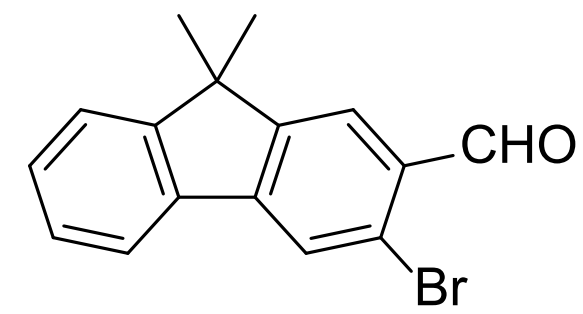

Following a published method [6], iPrMgCl LiCl (2.00 M; 61.9 mg, 301 µL, 601 µmol, 1.20 equiv) was added under an argon atmosphere to a cooled solution (−78 °) of 3-bromo-2-iodo-9,9-dimethylfluorene (**10**; 200 mg, 501 µmol, 1.00 equiv) in anhydrous THF (30 mL) and stirred for 15 min. Next, DMF (80.6 mg, 84.8 µL, 1.10 mmol, 2.20 equiv) was added and stirring continued for 1 h while allowing the mixture to warm to rt. NH_4Cl was added, the phases were separated, and the aqueous phase was extracted with CH_2Cl_2. The combined organic layers were dried over Na_2SO_4, filtered, and concentrated under reduced pressure. The obtained crude was purified by column chromatography (silica gel, *n*-hexane/CH_2Cl_2 2:1) to yield **11** (106 mg, 0.35 mmol, 70%) as a light-yellow solid.

R_f = 0.55 (*n*-hexane/CH_2Cl_2 1:2).

^{1}H NMR (400 MHz, $CDCl_3$): δ (ppm) 10.42 (s, 1 H, CHO), 7.98 (d, *J* = 20.3 Hz, 2 H, H-Ar), 7.78–7.71 (m, 1 H, H-Ar), 7.50–7.37 (m, 3 H, H-Ar), 1.50 (s, 6 H, CH_3).

^{13}C NMR (100 MHz, $CDCl_3$): δ (ppm) 192.2 (CHO), 155.3 (C), 153.3 (C), 147.0 (C), 136.6 (C), 131.9 (C), 129.8 (CH), 127.7 (CH), 126.9 (C), 125.2 (CH), 123.9 (CH), 123.2 (CH), 121.6 (CH), 47.2 (C), 26.9 (CH3).

IR (ATR) $\tilde{\nu}$ (cm^{-1}) = 2958 (w), 2863 (w), 2849 (w), 1680 (vs), 1596 (vs), 1560 (w), 1472 (m), 1456 (w), 1438 (m), 1391 (s), 1378 (s), 1361 (w), 1295 (w), 1266 (m), 1227 (m), 1217 (m), 1169 (vs), 1082 (m), 1030 (m), 1016 (m), 956 (s), 909 (m), 899 (m), 880 (s), 866 (m), 779 (m), 755 (vs), 731 (vs), 713 (m), 622 (w), 618 (w), 567 (m), 551 (w), 500 (m), 479 (w), 429 (vs), 402 (m), 378 (w).

MS (ESI): *m/z* (%): 303.0 (1) $[M+3]^+$, 302.9 (2) $[M+2]^+$, 301.0 (4) $[M+1]^+$, 300.0 (1) $[M]^+$, HRMS (ESI): *m/z* calcd for $C_{16}H_{13}BrO$: 300.0150 $[M]^+$; found: 300.0102.

9,9-Dimethyl-3-(4,4,5,5-tetramethyl-1,3,2-dioxaborolan-2-yl)-9*H*-fluorene-2-carbaldehyde (12)

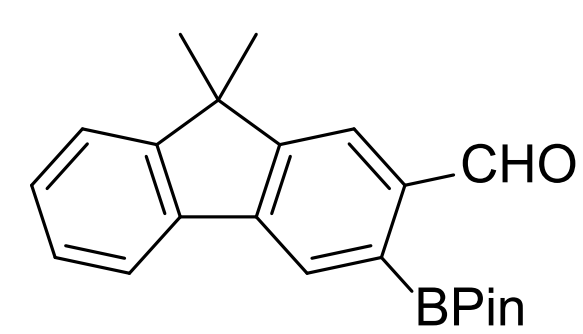

$PdCl_2$(dppf) (99.6 mg, 136 µmol, 0.100 equiv) was added under an argon atmosphere to a degassed (ultrasonication, 10 min) solution of 3-bromo-9,9-dimethylfluorene-2-carbaldehyde (**11**; 410 mg, 1.36 mmol, 1.00 equiv), KOAc (401 mg, 4.08 mmol, 3.00 equiv), and B_2Pin_2 (449 mg, 1.77 mmol, 1.30 equiv) in 1,4-dioxane (20 mL) and the mixture was heated to 80 °C for 16 h. After cooling to rt, the solvent was removed under reduced pressure. EtOAc (50 mL) was added and the solution filtered (Celite), concentrated at reduced pressure, and purified by column chromatography (silica gel, *n*-hexane/EtOAc 9:1) to yield **12** (280 mg, 0.80 mmol, 59%) as a white solid.

R_f = 0.26 (*n*-hexane/EtOAc 9:1).

^{1}H NMR (400 MHz, $CDCl_3$): *δ* (ppm) 10.66 (s, 1 H, CHO), 8.23 (s, 1 H, H-Ar), 8.09 (s, 1 H, H-Ar), 7.90–7.82 (m, 1 H, H-Ar), 7.49–7.45 (m, 1 H, H-Ar), 7.41–7.37 (m, 2 H, H-Ar), 1.51 (s, 6 H, CH_3), 1.43 (s, 12 H, CH_3).

^{13}C NMR (100 MHz, $CDCl_3$): *δ* (ppm) 194.8 (CHO), 156.5 (C), 154.9 (C), 144.2 (C), 140.8 (C), 137.9 (C), 128.9 (CH), 127.4 (CH), 127.4 (CH), 122.9 (CH), 121.9 (CH), 121.5 (CH), 84.6 (C), 47.3 (C), 26.9 (CH_3), 25.1 (CH_3). One signal was not observed due to the quadrupole moment of boron.

IR (ATR) $\tilde{\nu}$ (cm^{-1}) = 2962 (w), 2924 (w), 1677 (vs), 1599 (s), 1475 (w), 1460 (w), 1445 (w), 1412 (m), 1401 (w), 1381 (s), 1371 (s), 1354 (vs), 1320 (vs), 1292 (s), 1262 (vs), 1234 (m), 1211 (m), 1157 (s), 1140 (vs), 1122 (m), 1111 (s), 1091 (vs), 1048 (vs), 1020 (s), 967 (m), 958 (m), 924 (w), 908 (s), 858 (s), 830 (s), 800 (s), 788 (vs), 764 (vs), 748 (s), 738 (vs), 713 (s), 698 (vs), 669 (m), 647 (w), 612 (w), 578 (w), 569 (s), 555 (w), 484 (w), 433 (s), 426 (m), 392 (m), 382 (m).

MS (ESI): *m/z* (%): 350.2 (23) $[M+2]^+$, 349.1 (100) $[M+1]^+$, 348.2 (14) $[M]^+$, HRMS (ESI): *m/z* calcd for $C_{22}H_{25}BO_3$: 348.1897 $[M]^+$; found: 348.1946.

9'-Mesityl-9,9,9'',9''-tetramethyl-9*H*,9'*H*,9''*H*-[3,2':7',3''-terfluorene]-2,2''-dicarbaldehyde (14)

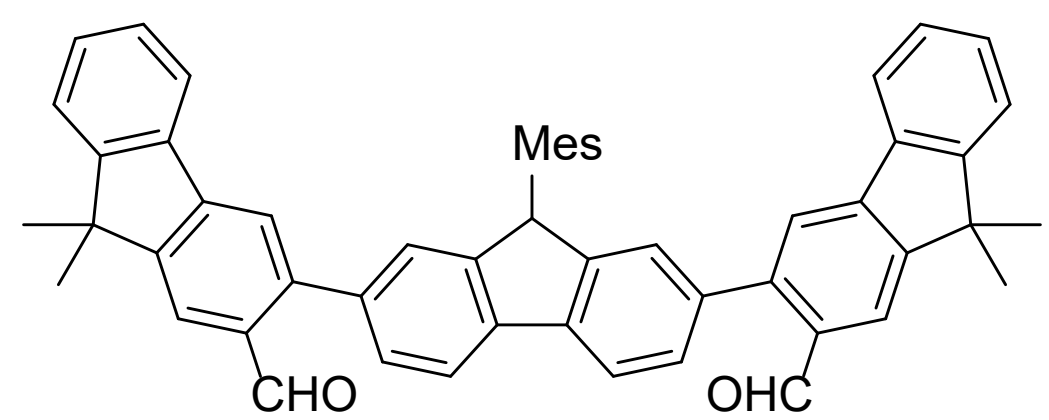

$PdCl_2$(dppf) (39.7 mg, 54.3 µmol, 0.150 equiv) was added under an argon atmosphere to a degassed (ultrasonication, 10 min) mixture of dibromofluorene **13** (160 mg, 362 µmol, 1.00 equiv), boronate **12** (277 mg, 796 µmol, 2.20 equiv), and Cs_2CO_3 (589 mg, 1.81 mmol, 5.00 equiv) in toluene/H_2O 1:1 (10 mL). The mixture was heated to 105 °C for 16 h, cooled to rt, and the aqueous layer was extracted with CH_2Cl_2 (3 × 100 mL). The combined organic layers were dried (Na_2SO_4), concentrated at reduced pressure, and purified by column chromatography (silica gel, *n*-hexane/EtOAc 9:1) to yield **14** (136 mg, 188 µmol, 52%) as a beige solid.

R_f = 0.26 (*n*-hexane/EtOAc 9:1).

^{1}H NMR (400 MHz, $CDCl_3$): *δ* (ppm) 9.99 (s, 2 H, CHO), 8.10 (s, 2 H, H-Ar), 8.02 (d, *J* = 7.8 Hz, 2 H, H-Ar), 7.80 (dd, *J* = 6.9, 1.6 Hz, 2 H, H-Ar), 7.76 (s, 2 H, H-Ar), 7.51 (ddd, *J* = 14.9, 7.4, 1.7 Hz, 4 H, H-Ar), 7.44–7.37 (m, 4 H, H-Ar), 7.34 (s, 2 H, H-Ar), 6.96 (s, 1 H, H-Ar), 6.68 (s, 1 H, H-Ar), 5.69 (s, 1 H, CH), 2.69 (s, 3 H, CH_3), 2.23 (s, 3 H, CH_3), 1.55 (s, 12 H, CH_3), 1.26 (s, 3 H, CH_3).

^{13}C NMR (100 MHz, $CDCl_3$): *δ* (ppm) 192.4 (CHO), 155.4 (C), 153.1 (C), 148.1 (C), 146.4 (C), 144.8 (C), 140.3 (C), 138.0 (C), 137.8 (C), 137.7 (C), 137.6 (C), 136.8 (C), 133.0 (C), 130.9 (C), 129.5 (CH), 129.2 (CH), 127.5 (CH), 126.0 (CH), 123.1 (CH), 122.2 (CH), 121.7 (CH), 121.5 (CH), 120.3 (CH), 50.1 (CH), 47.2 (C), 27.1 (CH_3), 27.1 (CH_3), 22.0 (CH_3), 20.9 (CH_3), 19.1 (CH_3).

IR (ATR) $\tilde{\nu}$ (cm^{-1}) = 2961 (w), 2919 (w), 1677 (s), 1608 (m), 1458 (w), 1443 (w), 1395 (w), 1259 (vs), 1159 (w), 1153 (w), 1146 (w), 1078 (vs), 1014 (vs), 963 (w), 907 (w), 891 (w), 866 (w), 858 (w), 795 (vs), 758 (s), 742 (vs), 727 (m), 707 (m), 671 (m), 633 (w), 615 (w), 601 (w), 569 (w), 557 (w), 548 (w), 500 (w), 482 (w), 432 (m), 402 (s), 387 (s), 382 (s).

MS (ESI): *m/z* (%): 725.3 (15) $[M+1]^+$, 724.4 (1) $[M]^+$, 723.4 (14) $[M-1]^+$, HRMS (ESI): *m/z* calcd for $C_{54}H_{44}O_2$: 724.3341 $[M]^+$; found: 724.3290.

(9'-Mesityl-9,9,9'',9''-tetramethyl-9*H*,9'*H*,9''*H*-[3,2':7',3''-terfluorene]-2,2''-diyl)bis(mesityl-methanol) (15)

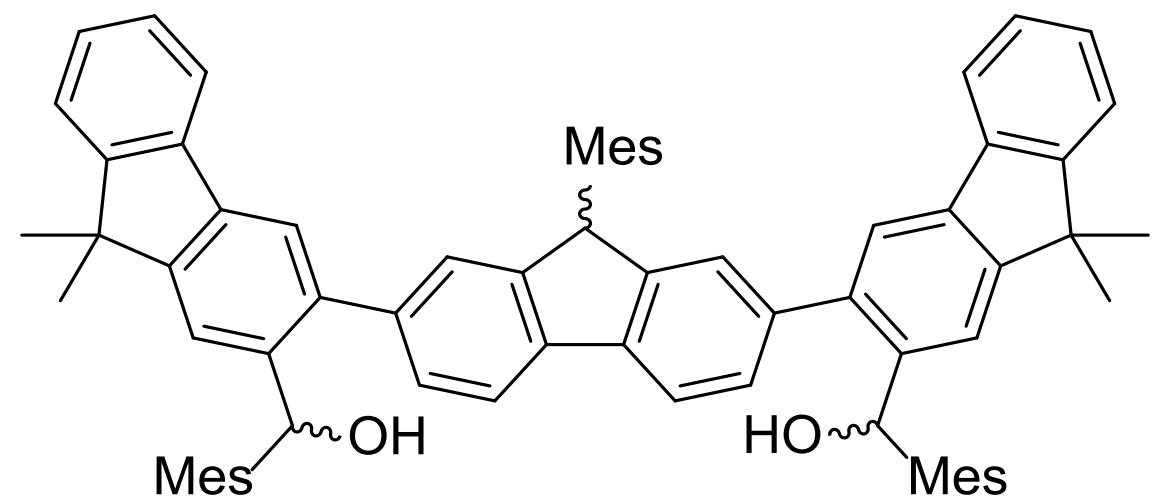

Following a published method [7], MesMgBr (1.00 M in THF; 151 mg, 676 µL, 676 µmol, 5.00 equiv) was added dropwise under an argon atmosphere to a cooled (0 °C) solution of biscarbaldehyde **14** (98.0 mg, 135 µmol, 1.00 equiv) in anhydrous THF (15 mL). After the addition was completed, the cooling bath was removed and the mixture stirred for 15 min at rt. Saturated aqueous NH_4Cl solution (20 mL) was added, stirring continued for 10 min, and the mixture extracted with CH_2Cl_2 (3 × 100 mL). The combined organic layers were dried (Na_2SO_4) and concentrated at reduced pressure.

Radical precursor 16[1]

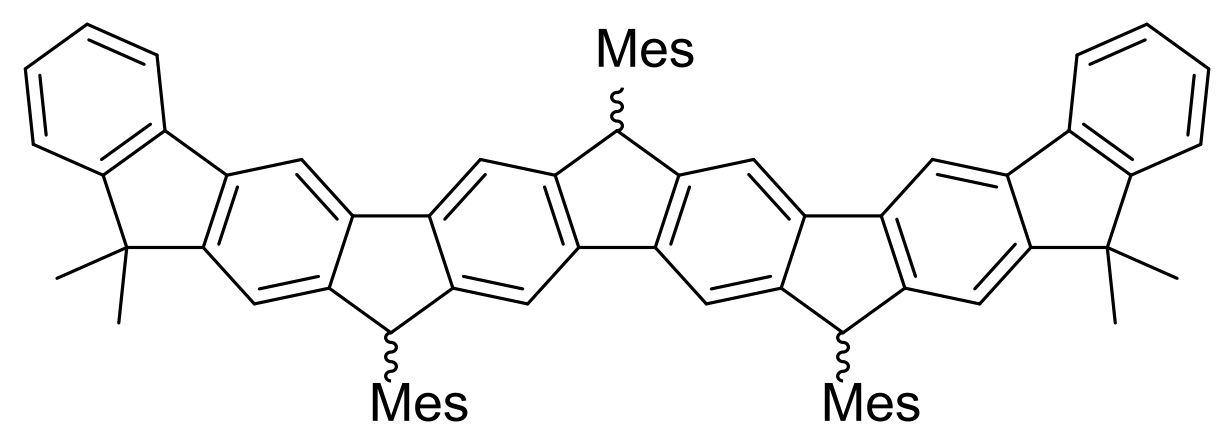

Following a published method [7], the remnant from the previous step (**15**) was dissolved in anhydrous CH_2Cl_2 (20 mL) and the solution was cooled to 0 °C. $BF_3·OEt_2$ (210 mg, 938 µL, 938 µmol, 5.00 equiv) was added under an argon atmosphere and the mixture was stirred for 1 h at rt. Saturated aqueous NH_4Cl solution (20 mL) was added, stirring continued for 10 min, and the mixture extracted with CH_2Cl_2 (3 × 100 mL). The combined organic layers were dried (Na_2SO_4), concentrated at reduced pressure, and purified by column chromatography (silica gel, *n*-pentane/CH_2Cl_2 4:1) to yield **16** (127 mg, 137 µmol, 73%) as a pink solid. The product was obtained as a mixture of diastereoisomers, thus resulting in additional hardly discriminable signals in the spectra.

R_f = 0.31 (*n*-pentane/CH_2Cl_2 4:1).

^{1}H NMR (400 MHz, $CDCl_3$): *δ* (ppm) 8.1 (d, *J* = 9.6 Hz, 2 H, H-Ar), 7.8 (d, *J* = 7.3 Hz, 2 H, H-Ar), 7.7 (s, 2 H, H-Ar), 7.5 (d, *J* = 8.0 Hz, 2 H, H-Ar), 7.4 (d, *J* = 7.2 Hz, 2 H, H-Ar), 7.3 (s, 2 H, H-Ar), 7.3 (d, *J* = 7.2 Hz, 2 H, H-Ar), 7.2 (s, 3 H, H-Ar), 7.1 (s, 2 H, H-Ar), 6.7 (d, *J* = 17.9 Hz, 3 H, H-Ar), 5.7 (s, 1 H, CH), 5.6 (d, *J* = 10.1 Hz, 2 H, CH), 2.8 (d, *J* = 54.4 Hz, 9 H, CH_3), 2.4–2.3 (m, 9 H, CH_3), 1.5 (d, *J* = 5.2 Hz, 6 H, CH_3), 1.4 (d, *J* = 4.8 Hz, 6 H, CH_3), 1.1 (s, 9 H, CH_3).

^{13}C NMR (100 MHz, $CDCl_3$): *δ* (ppm) 154.0 (C), 153.6 (C), 147.8 (C), 146.9 (C), 146.6 (C), 140.7 (C), 140.5 (C), 140.3 (C), 139.5 (C), 138.4 (C), 137.9 (C), 136.3 (C), 134.7 (C), 130.9 (CH), 129.0 (CH), 127.1 (CH), 122.7 (CH), 119.9 (CH), 118.4 (CH), 115.9 (CH), 115.6 (CH), 111.5 (CH), 49.6 (CH), 46.8 (C), 27.5 (CH_3), 22.8 (CH_3), 22.1 (CH_3), 21.1 (CH_3), 18.7 (CH_3), 18.6 (CH_3).

IR (ATR) $\tilde{\nu}$ (cm^{-1}) = 2953 (s), 2919 (vs), 2851 (vs), 1723 (vw), 1609 (w), 1479 (m), 1456 (vs), 1431 (s), 1412 (m), 1377 (m), 1358 (w), 1288 (vs), 1261 (w), 1239 (w), 1221 (m), 1153 (w), 1084 (w),

[1] The IUPAC name of this compound might be: 14,16,19,21-tetrahydro-7,16,19-trimesityl-14,14,21,21-tetramethyl-7*H*-fluoreno[2'',3'':1,2;6'',7'':1',2']dicyclopenta[4,5-*b*:4',3'-*b*']difluoren.

1028 (m), 1016 (m), 936 (w), 887 (m), 880 (m), 849 (vs), 807 (m), 792 (s), 776 (s), 755 (vs), 744 (vs), 722 (vs), 616 (w), 571 (m), 564 (m), 480 (m), 429 (m), 399 (m), 385 (w), 375 (w).

MS (ESI): *m/z* (%): 931.5 (31) $[M+3]^+$, 930.5 (56) $[M+2]^+$, 929.5 (83) $[M+1]^+$, 928.4 (41) $[M]^+$, HRMS (ESI): *m/z* calcd for $C_{72}H_{64}$: 928.5008 $[M]^+$; found: 928.4968.

Radical 7[2]

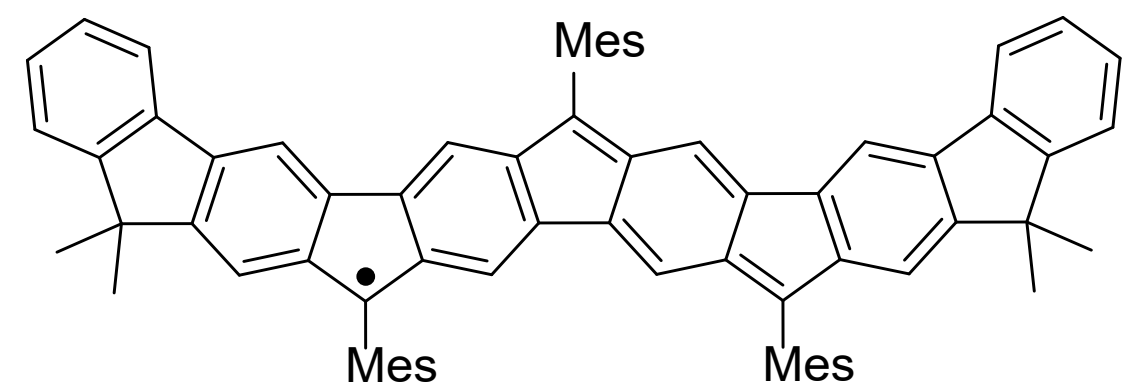

Following a published method [8], precursor **16** (125 mg, 135 µmol, 1.00 equiv) and *t*-BuOK (211 mg, 1.88 mmol, 14.0 equiv) were dissolved in anhydrous THF (15 mL) under an argon atmosphere and the mixture was heated for 16 h to 50 °C. The mixture was allowed to cool to rt and *p*-chloranil (2,3,5,6-tetrachlorocyclohexa-2,5-diene-1,4-dione; 149 mg, 605 µmol, 4.50 equiv) was added. After stirring for 10 min, the mixture was concentrated at reduced pressure. The obtained remnant was purified by column chromatography (silica gel, *n*-pentane/CH_2Cl_2 4:1) to yield radical **7** (101 mg, 109 µmol, 81%) as a dark green solid.

R_f = 0.29 (*n*-pentane/CH_2Cl_2 4:1).

IR (ATR, cm^{-1}): $\tilde{v}$ = 2953 (m), 2917 (vs), 2853 (s), 1672 (w), 1568 (w), 1513 (w), 1455 (m), 1436 (m), 1429 (m), 1374 (m), 1358 (m), 1340 (m), 1316 (w), 1290 (m), 1259 (m), 1232 (w), 1215 (w), 1198 (w), 1177 (w), 1154 (w), 1129 (s), 1098 (m), 1085 (m), 1064 (s), 1021 (s), 970 (w), 882 (vs), 863 (m), 849 (s), 803 (s), 781 (s), 749 (vs), 737 (s), 722 (s), 710 (s), 688 (m), 657 (w), 571 (m), 557 (m), 469 (w), 419 (w), 399 (m).

MS (ESI): *m/z* (%): 928.4 (8) $[M+3]^+$, 927.4 (30) $[M+2]^+$, 926.4 (78) $[M+1]^+$, 925.4 (100) $[M]^+$, HRMS (ESI): *m/z* calcd for $C_{72}H_{61}$: 925.4773 $[M]^+$; found: 925.4787.

2 The IUPAC name of this compound might be: 14,21-dihydro-7,16,19-trimesityl-14,14,21,21-tetramethylfluoreno[2'',3'':1,2;6'',7'':1',2']dicyclopenta[4,5-*b*:4',3'-*b*']difluoren-16-yl radical.

2. NMR spectra of all compounds and mass spectra

^{1}H NMR spectrum of 3-bromo-9,9-dimethyl-9*H*-fluoren-2-amine (**9**, 400 MHz, $CDCl_3$)

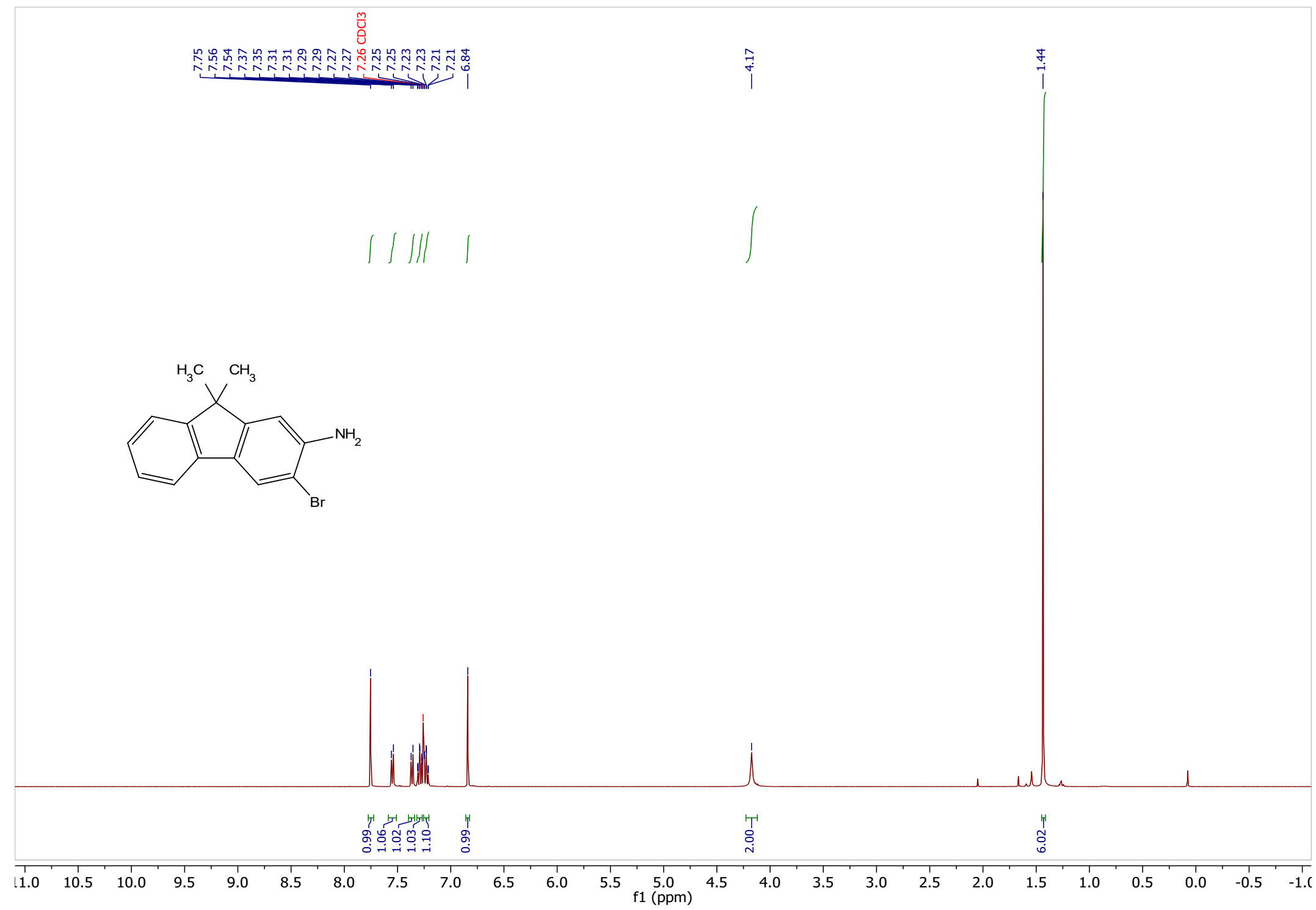

^{1}H NMR spectrum of 3-bromo-2-iodo-9,9-dimethyl-9*H*-fluorene (**10**, 400 MHz, $CDCl_3$)

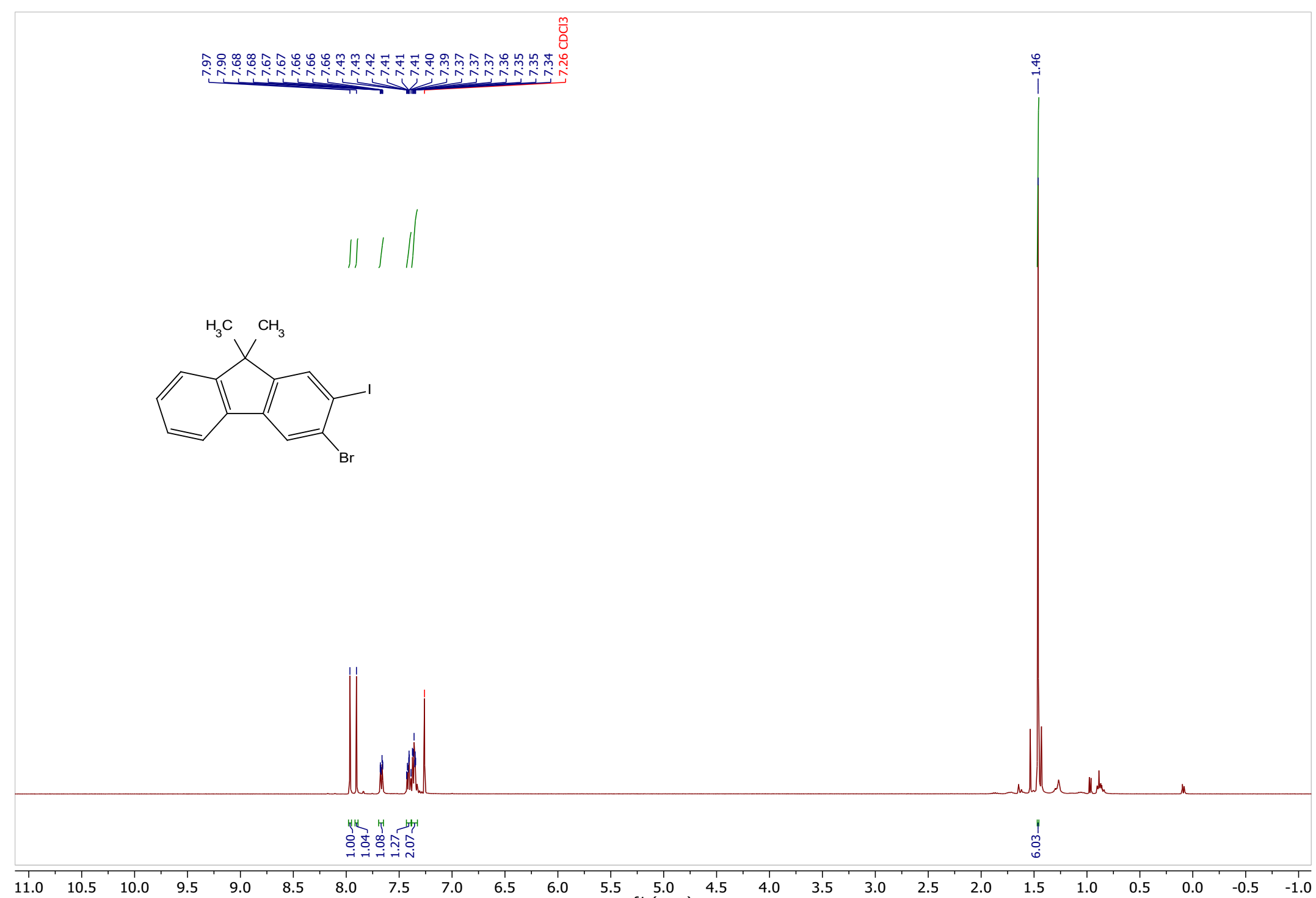

^{1}H NMR spectrum of 3-bromo-9,9-dimethyl-9*H*-fluorene-2-carbaldehyde (**11**, 400 MHz, $CDCl_3$)

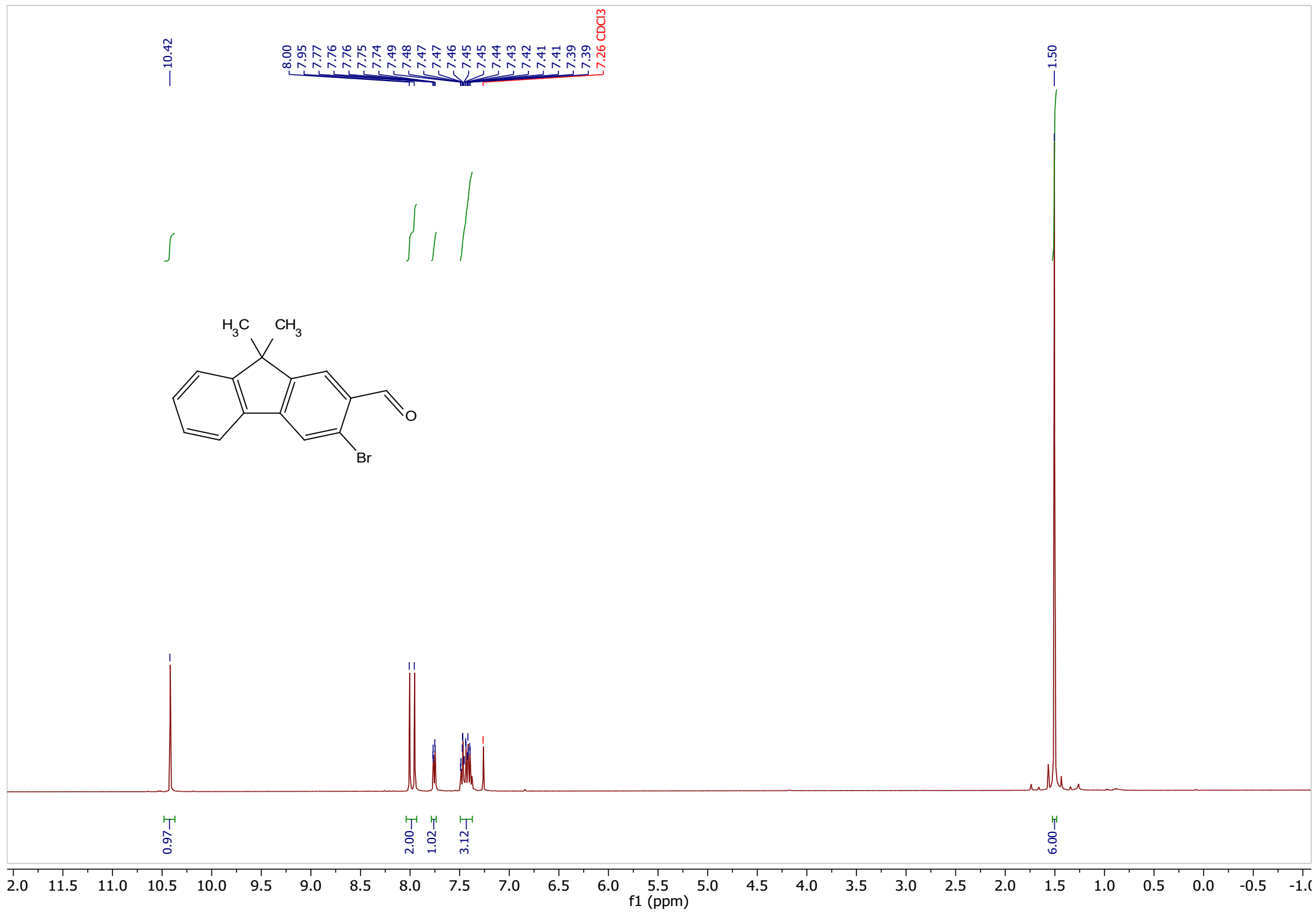

^{13}C NMR spectrum of 3-bromo-9,9-dimethyl-9*H*-fluorene-2-carbaldehyde (**11**, 100 MHz, $CDCl_3$)

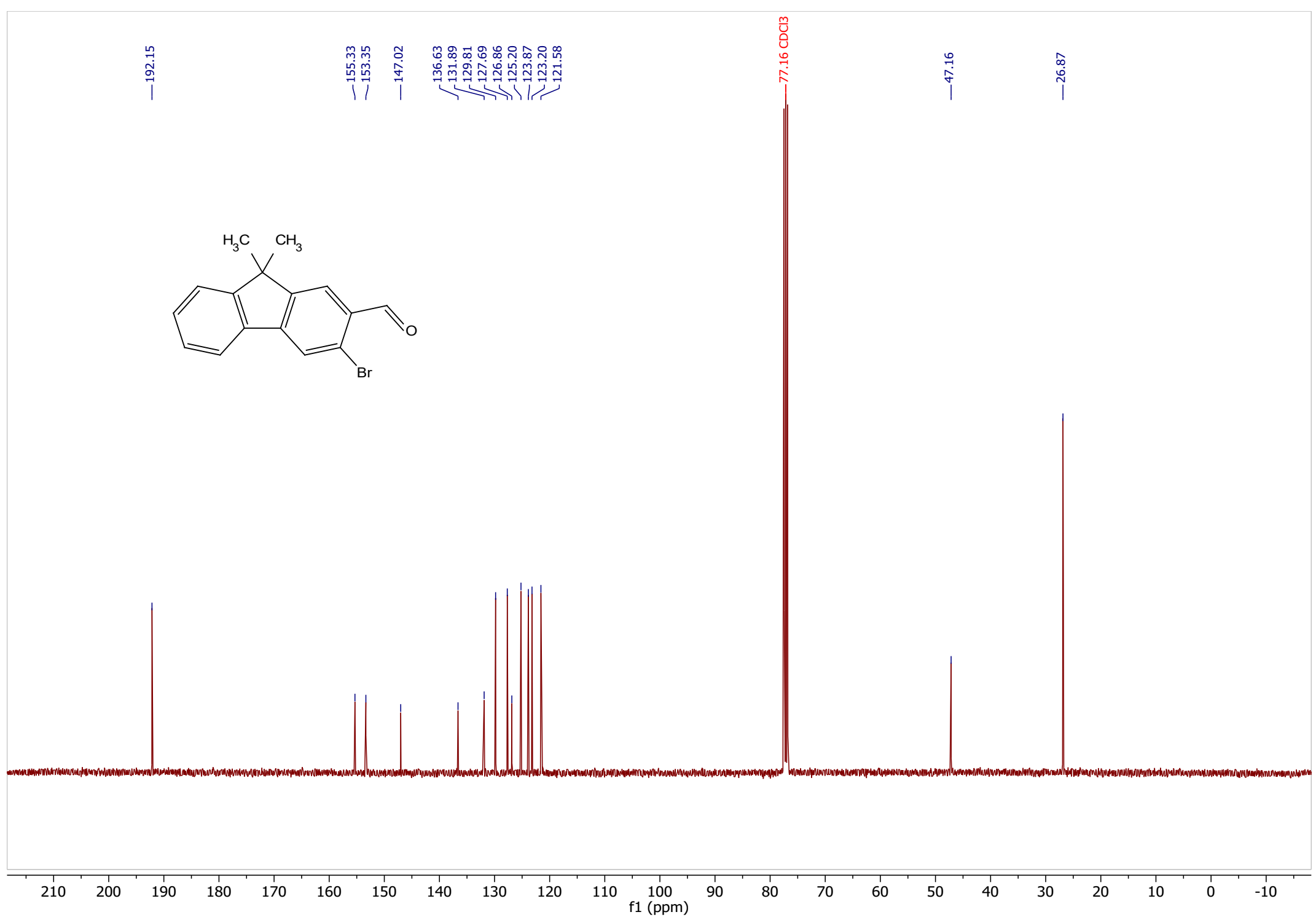

^{1}H NMR spectrum of 9,9-dimethyl-3-(4,4,5,5-tetramethyl-1,3,2-dioxaborolan-2-yl)-9*H*-fluorene-2-carbaldehyde (**12**, 400 MHz, $CDCl_3$)

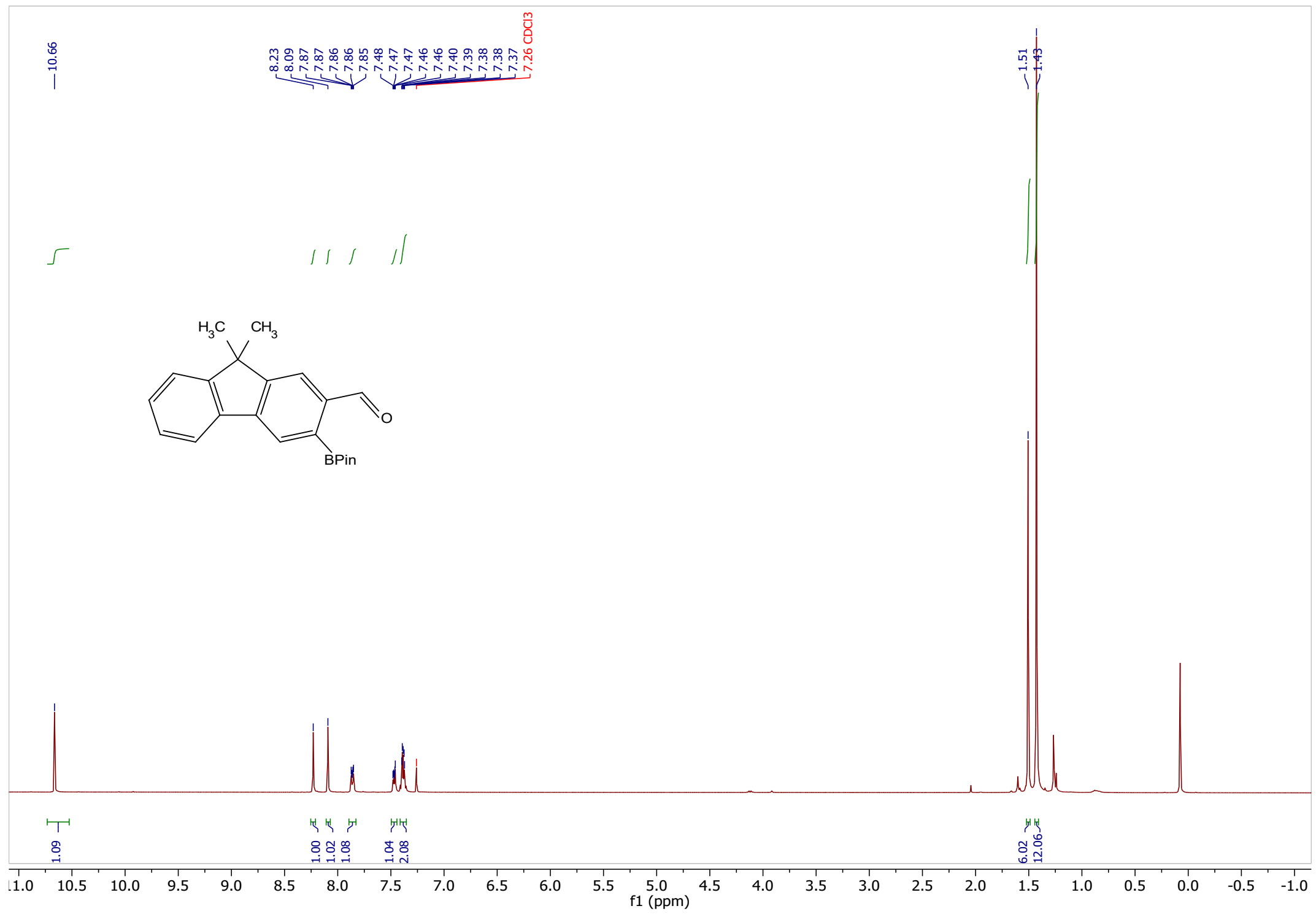

^{13}C NMR spectrum of 9,9-dimethyl-3-(4,4,5,5-tetramethyl-1,3,2-dioxaborolan-2-yl)-9*H*-fluorene-2-carbaldehyde (**12**, 100 MHz, $CDCl_3$)

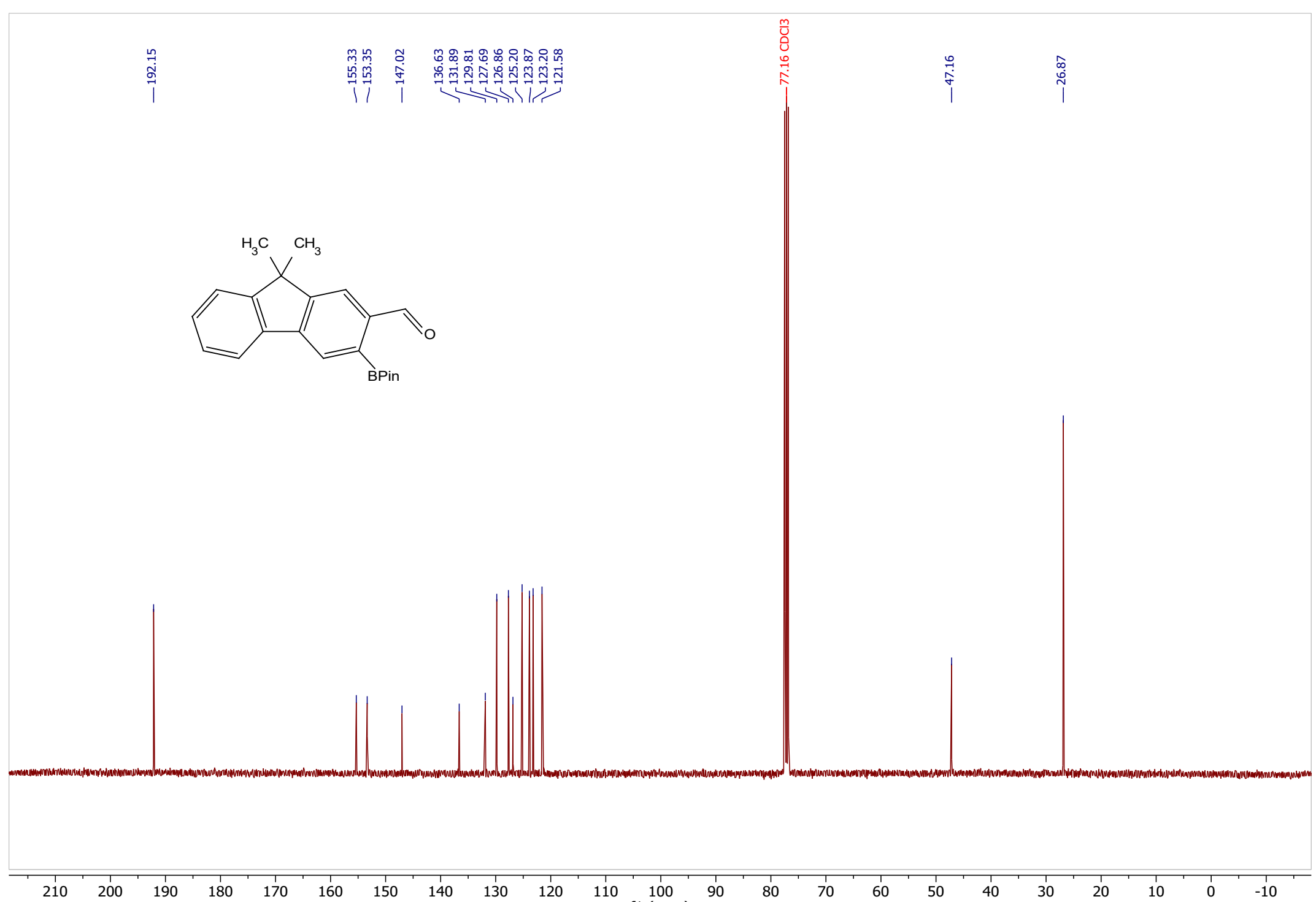

^{1}H NMR spectrum of 9’-mesityl-9,9,9’’,9’’-tetramethyl-9*H*,9’H,9’’*H*-[3,2’:7’,3’’-terfluorene]-2,2’’-dicarbaldehyde (**14**, 400 MHz, $CDCl_3$)

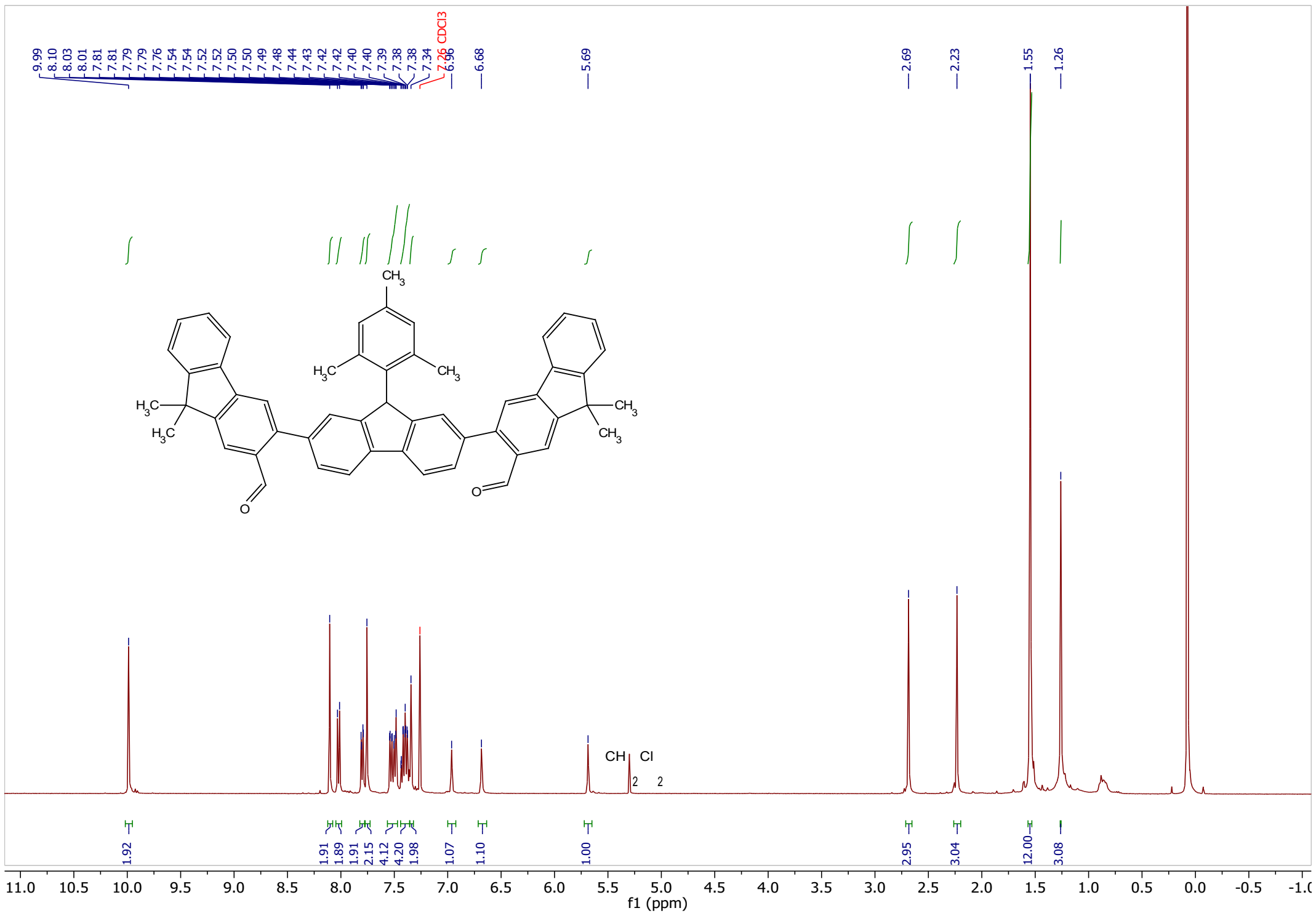

^{13}C NMR spectrum of 9’-mesityl-9,9,9’’,9’’-tetramethyl-9*H*,9’H,9’’*H*-[3,2’:7’,3’’-terfluorene]-2,2’’-dicarbaldehyde (**14**, 100 MHz, $CDCl_3$)

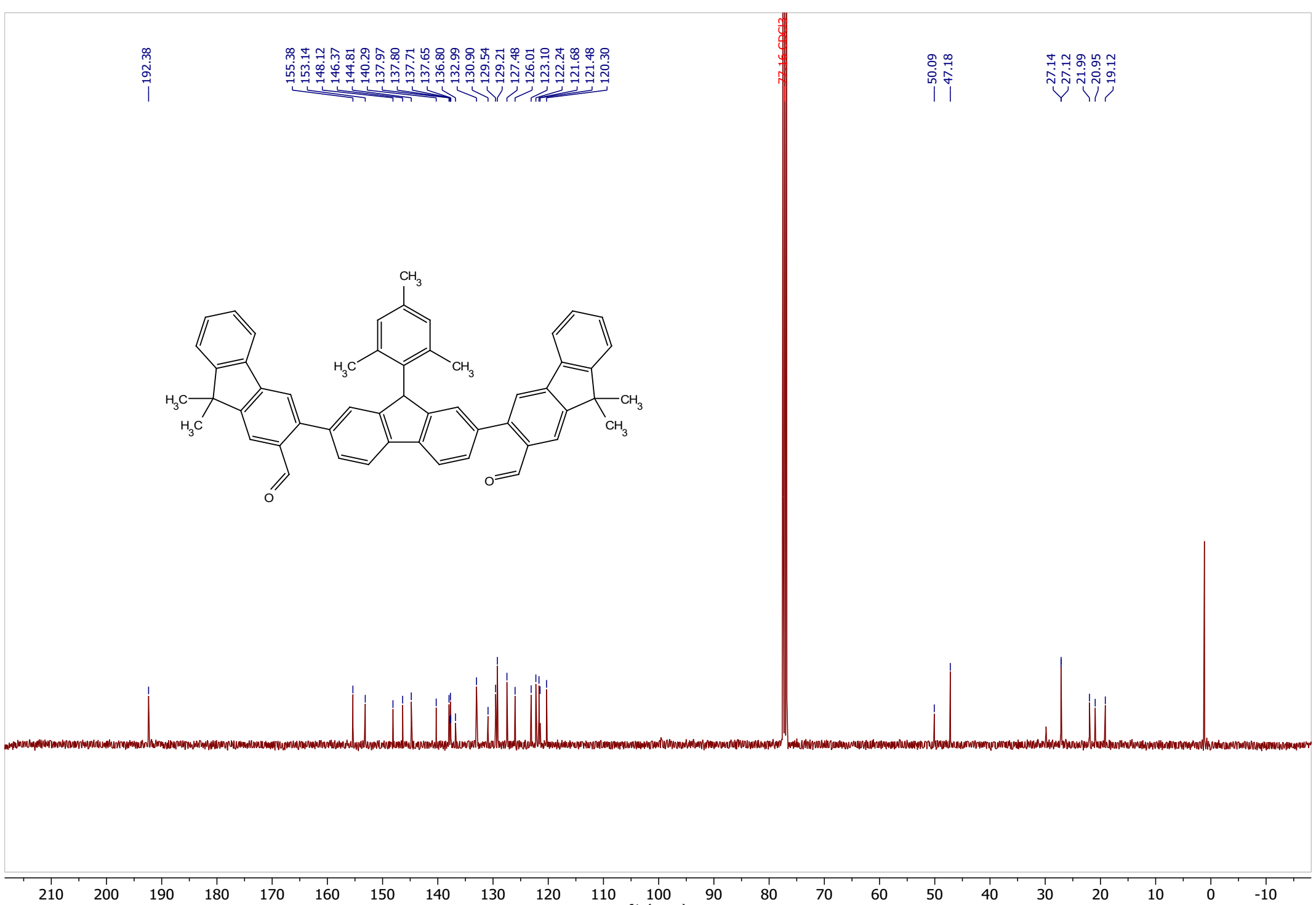

^{1}H NMR spectrum of radical precursor **16** (400 MHz, $CDCl_3$)

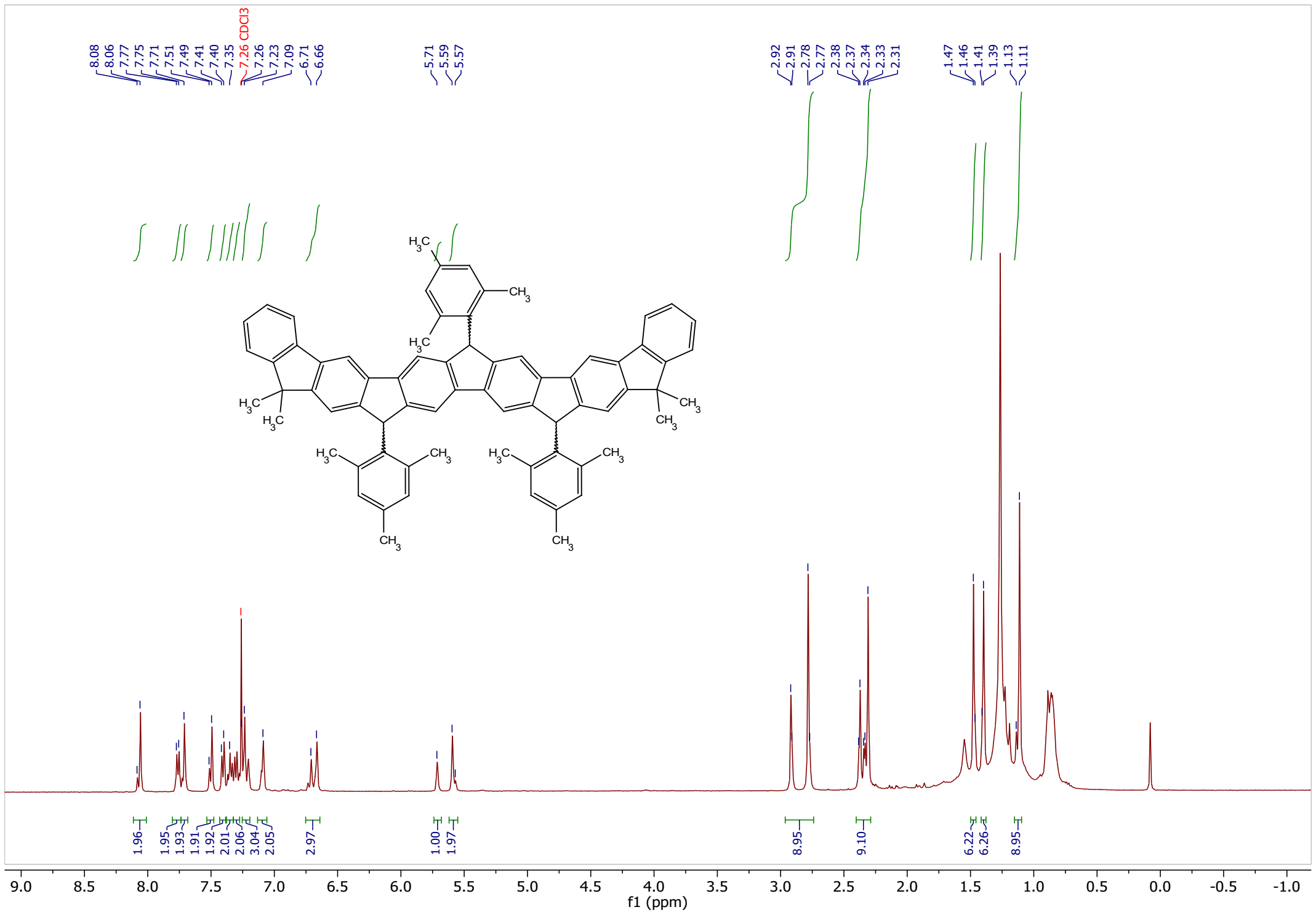

^{13}C NMR spectrum of radical precursor **16** (100 MHz, $CDCl_3$)

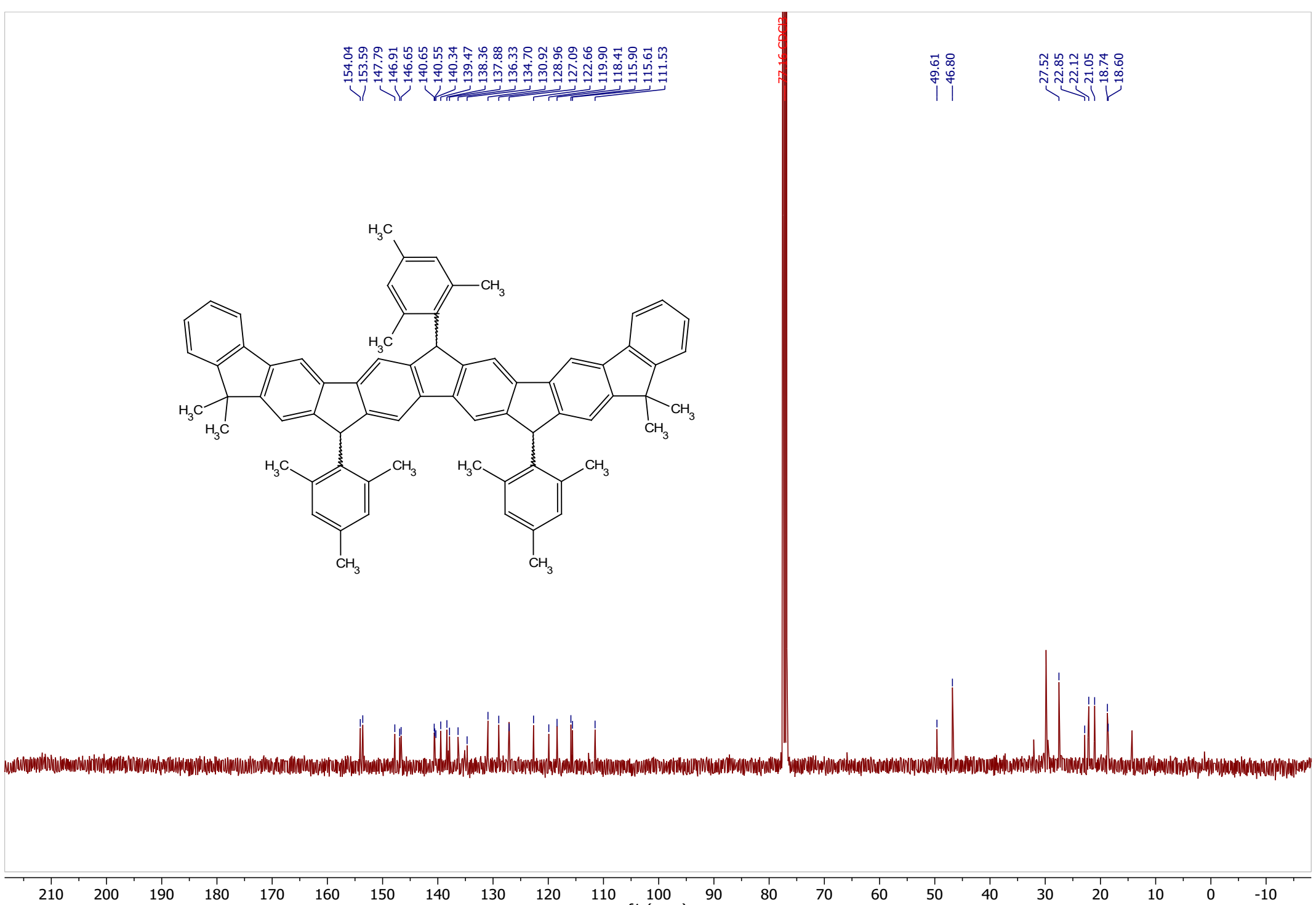

Mass spectrum of 3-bromo-9,9-dimethyl-9*H*-fluorene-2-carbaldehyde (**11**, ESI)

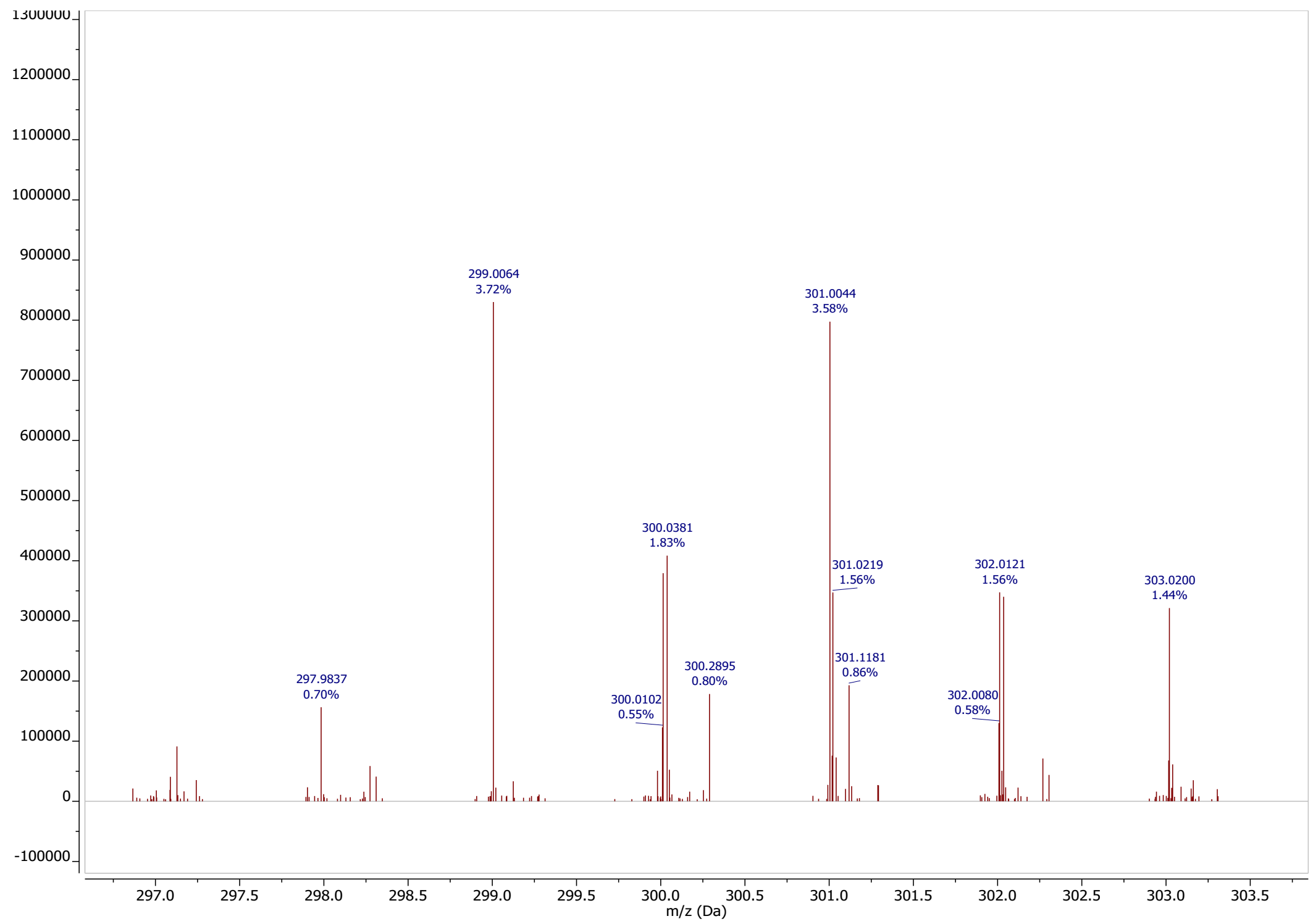

Mass spectrum of 9,9-dimethyl-3-(4,4,5,5-tetramethyl-1,3,2-dioxaborolan-2-yl)-9*H*-fluorene-2-carbaldehyde (**12**, ESI)

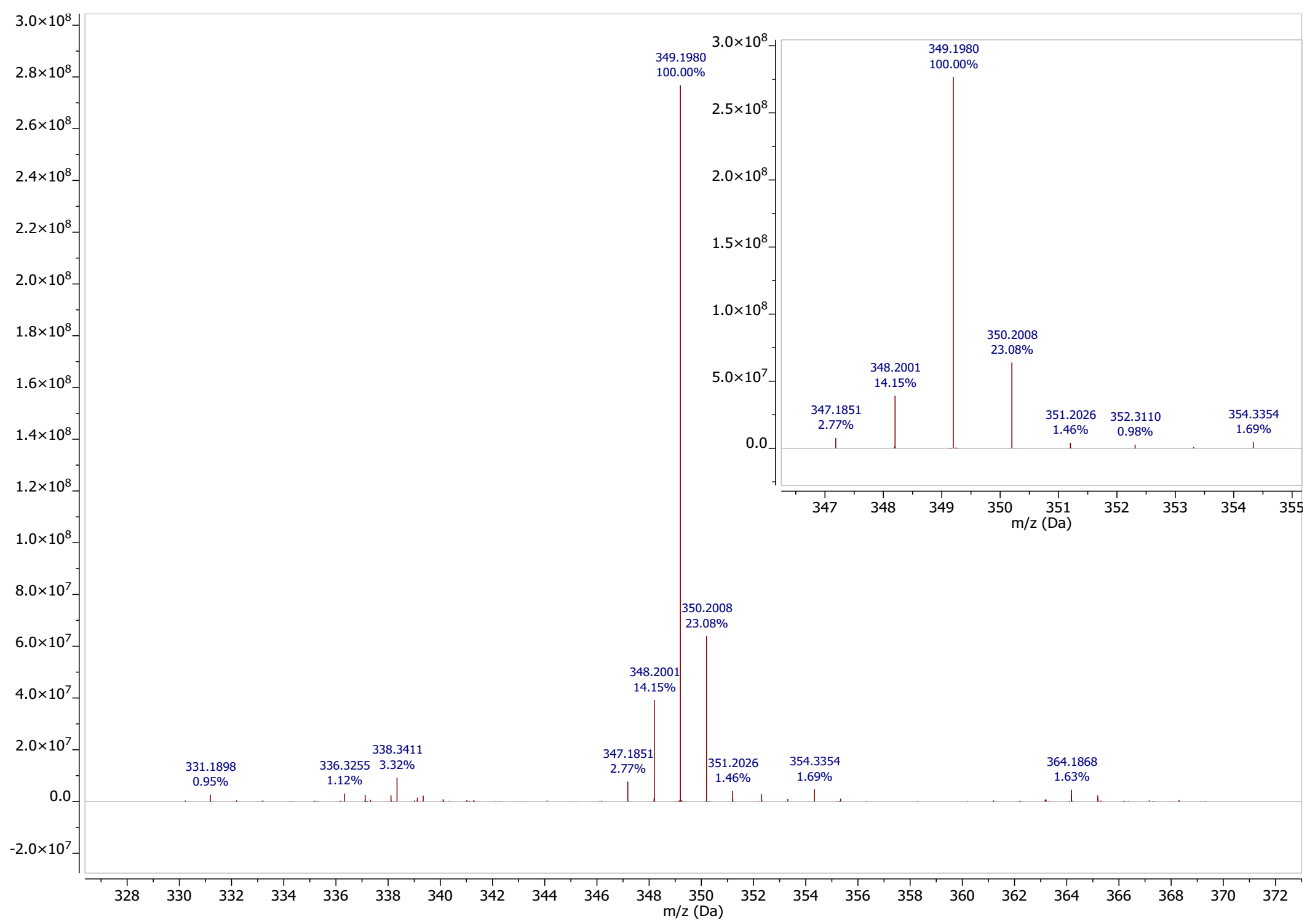

Mass spectrum of 9’-mesityl-9,9,9’’,9’’-tetramethyl-9*H*,9’H,9’’*H*-[3,2’:7’,3’’-terfluorene]-2,2’’-dicarbaldehyde (**14**, ESI)

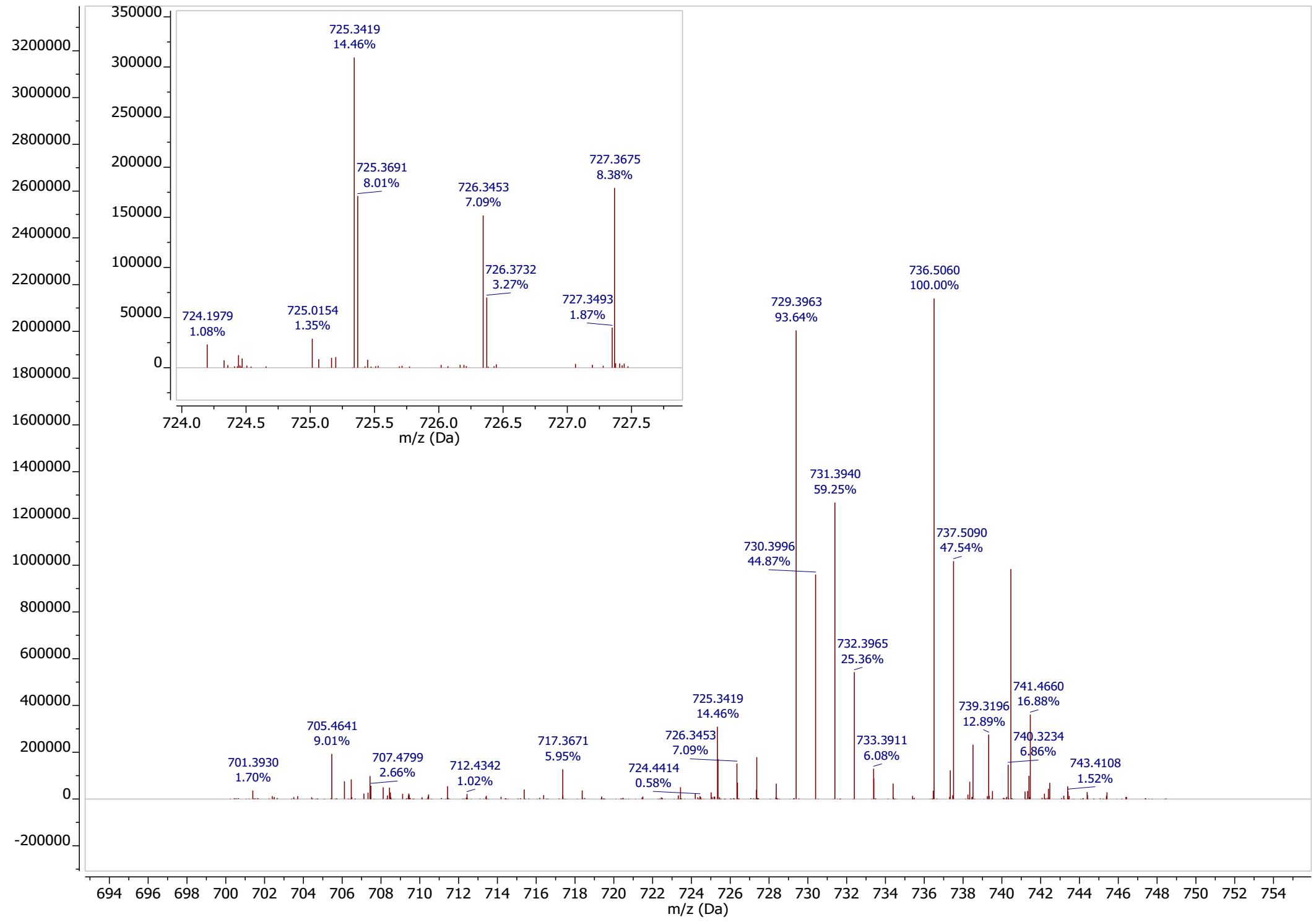

Mass spectrum of radical precursor **16** (ESI)

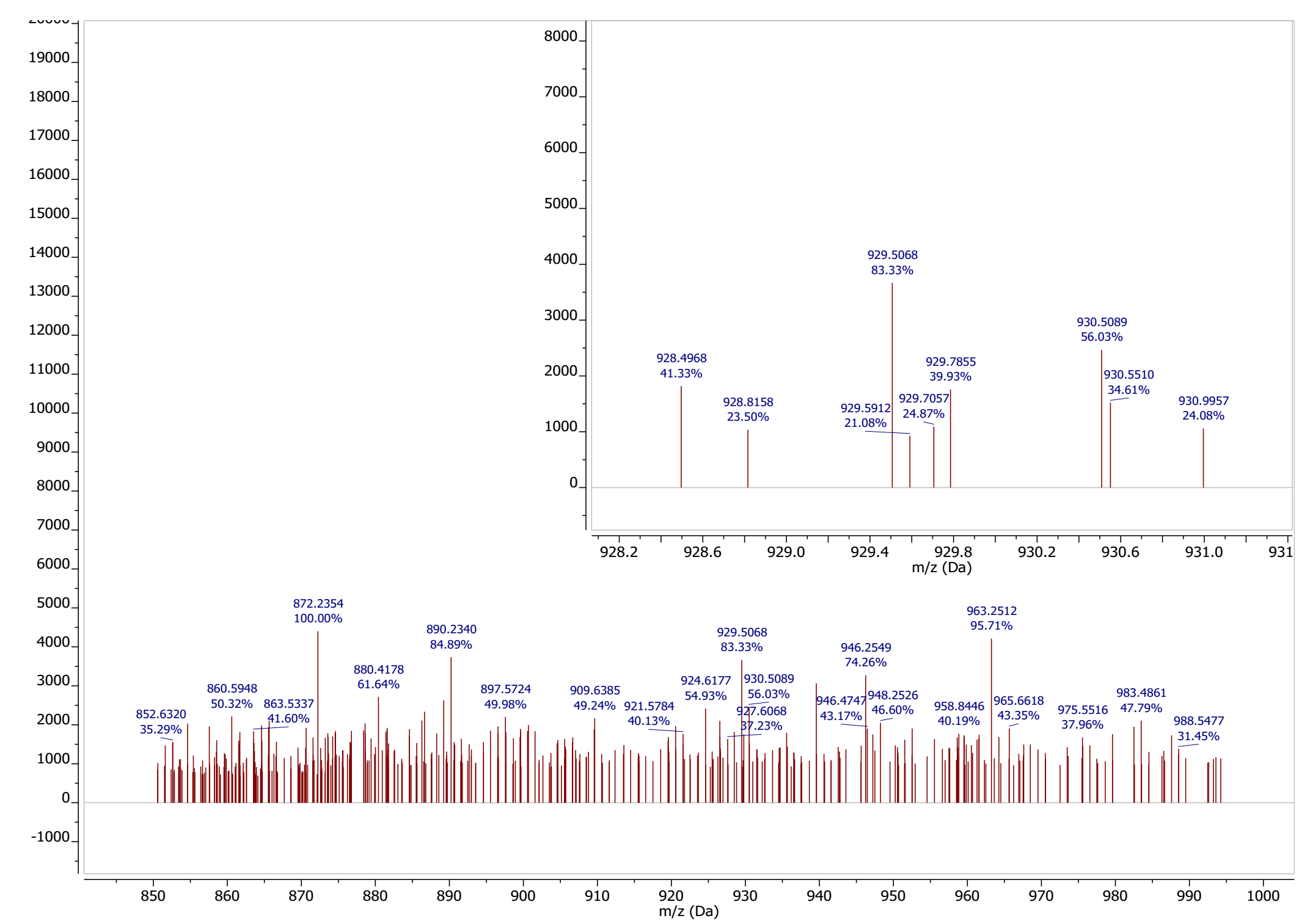

Mass spectrum of radical **7** (ESI)

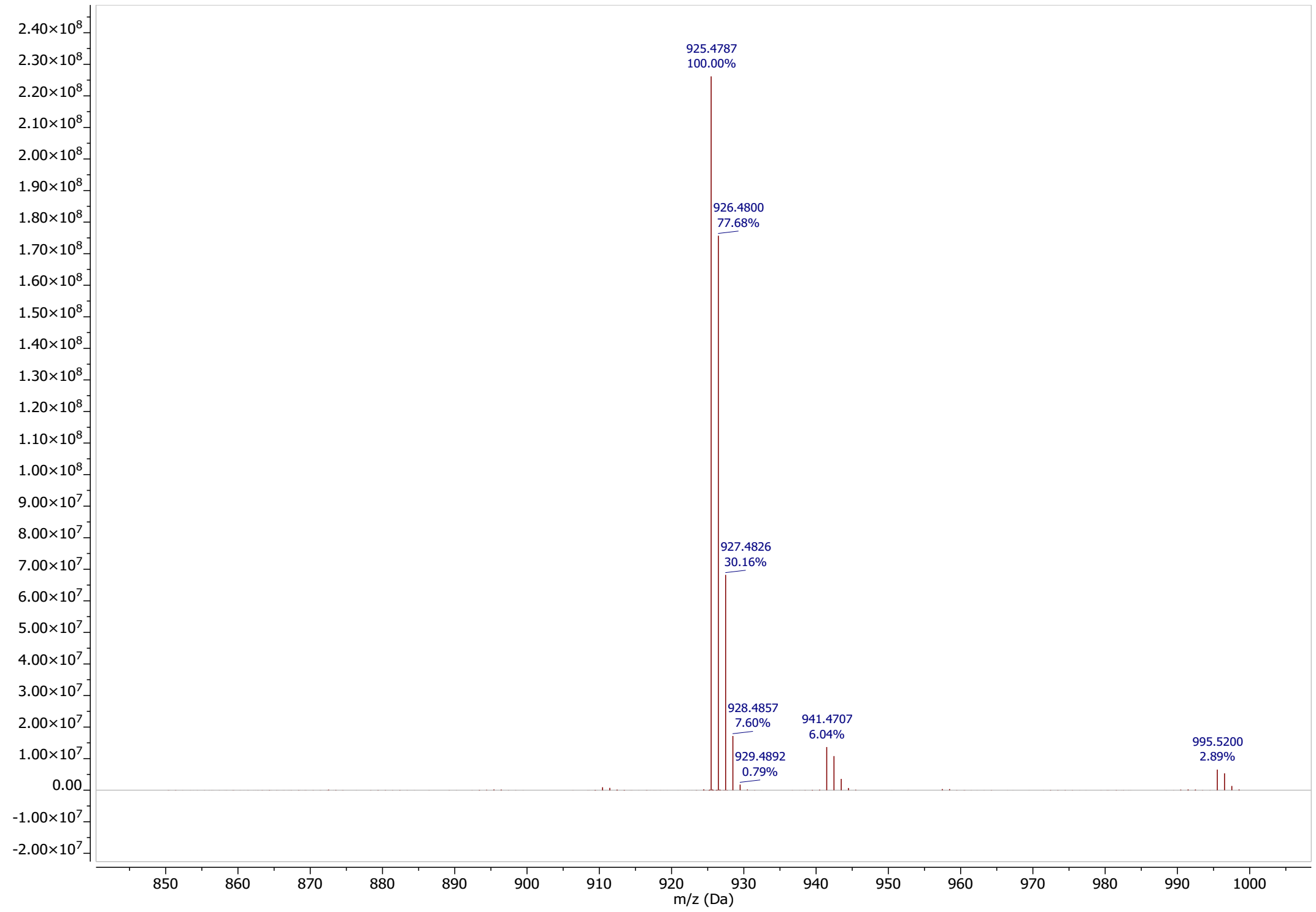

3. UV–vis spectrum

UV–vis spectrum of radical **7** recorded in CH_2Cl_2.

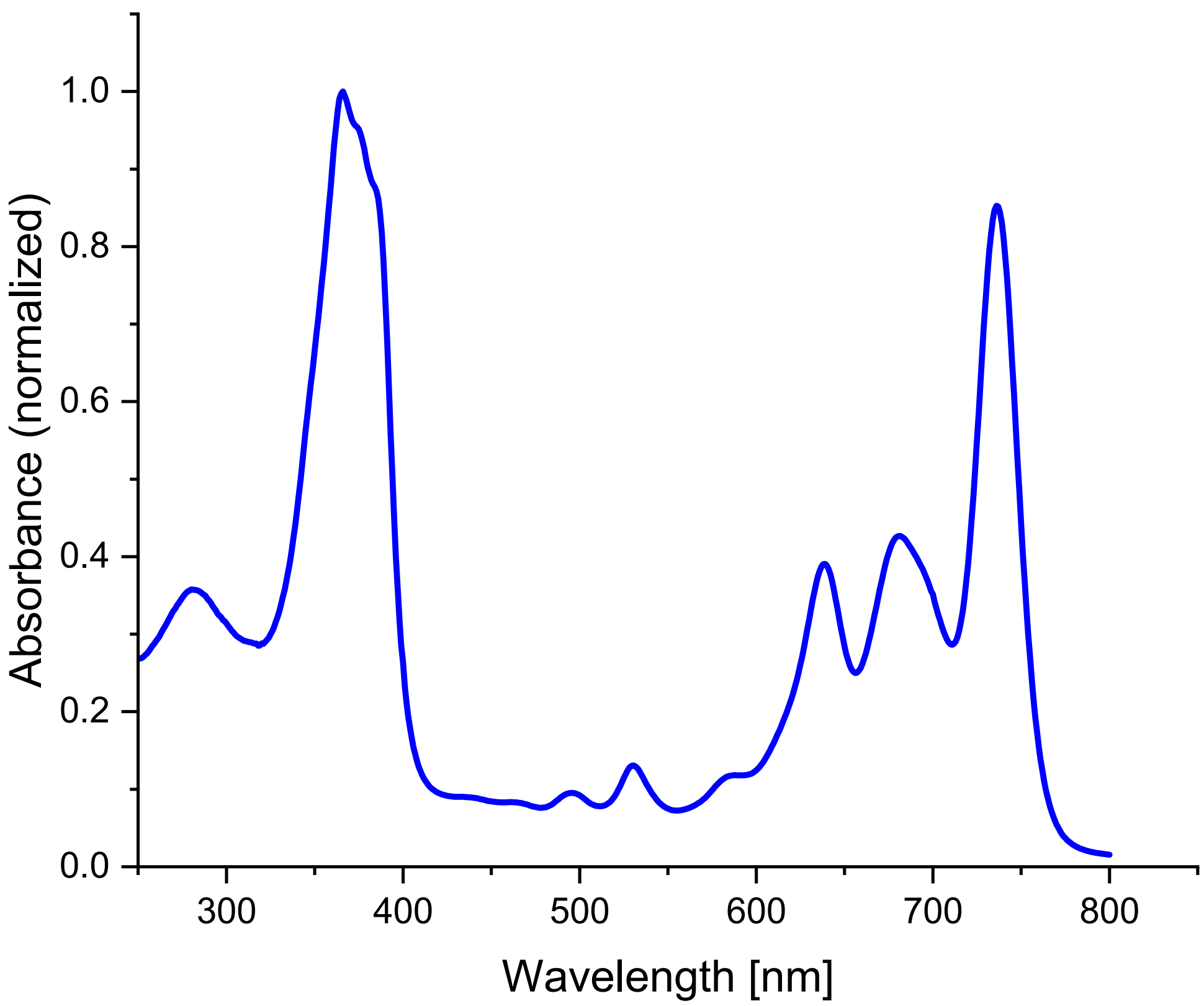

4. EPR spectra

A CW X-band [9.4214 GHz] EPR spectrum of radical **7** was measured (black) in toluene (1 mM) at ambient temperature showing a single resonance signal centered at g = 2.0038. The spectrum was referenced against a DPPH sample measured before with a g-factor of 2.0036. A simulated spectrum is given in red (S = ½, no hyperfine couplings, lwpp = 0.59). Further details: 1.00 G modulation amplitude, 100 kHz modulation frequency, 10 scans, 2048 points per measurement, 2.00 mW microwave power, 5.00 ms conversion time, 1.28 ms time constant.

Even with reduced modulation amplitude (0.20 G or 0.02 G) no discernible hyperfine coupling was observed.

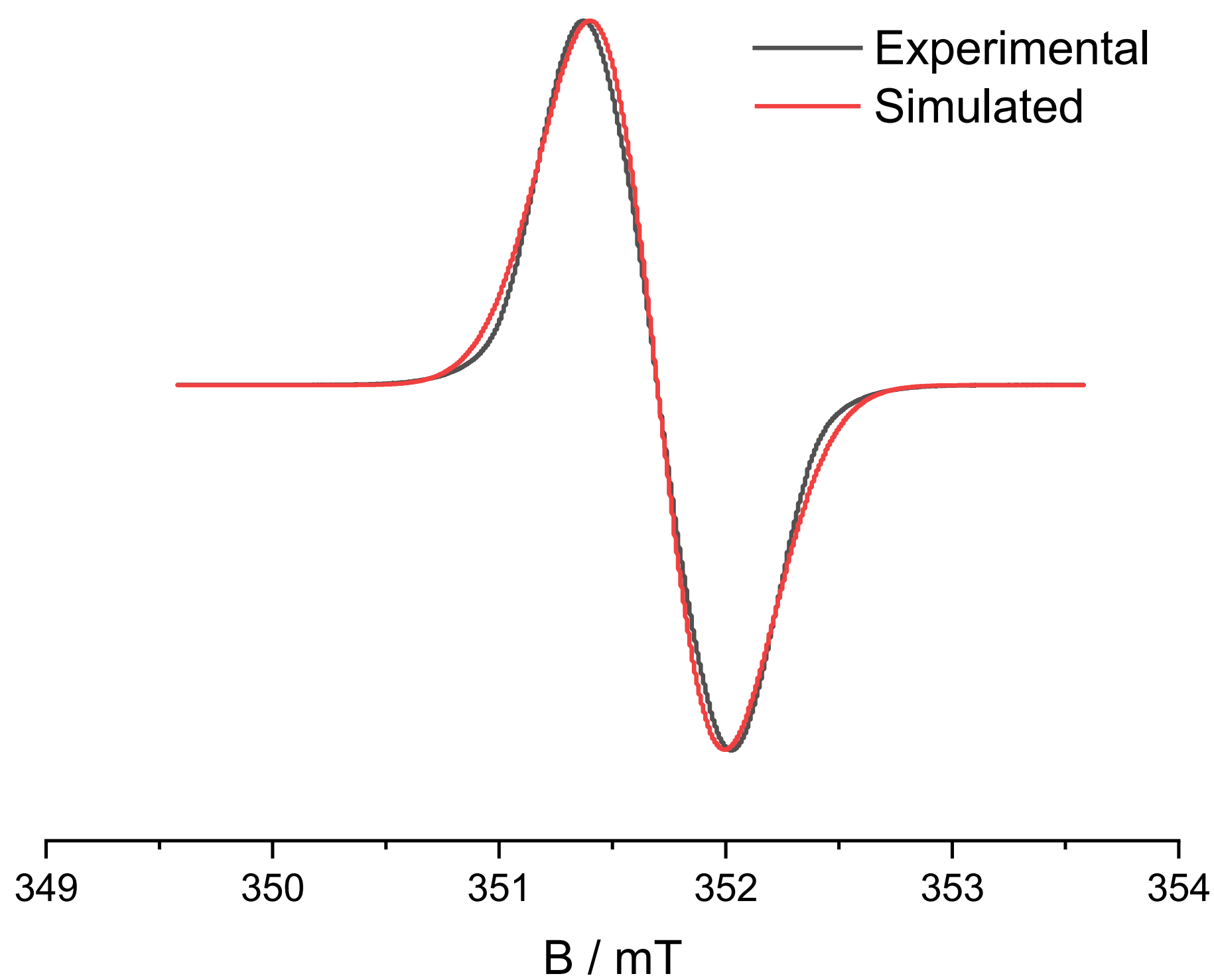

5. Cyclic voltammetry (CV)

Half-wave potentials of radical **7** in THF vs. Fc/Fc⁺ (internal standard; Fc*, decamethylferrocene); conditions: Pt/[NBu_4][PF_6]/Ag, *v* = 100 mV/s.

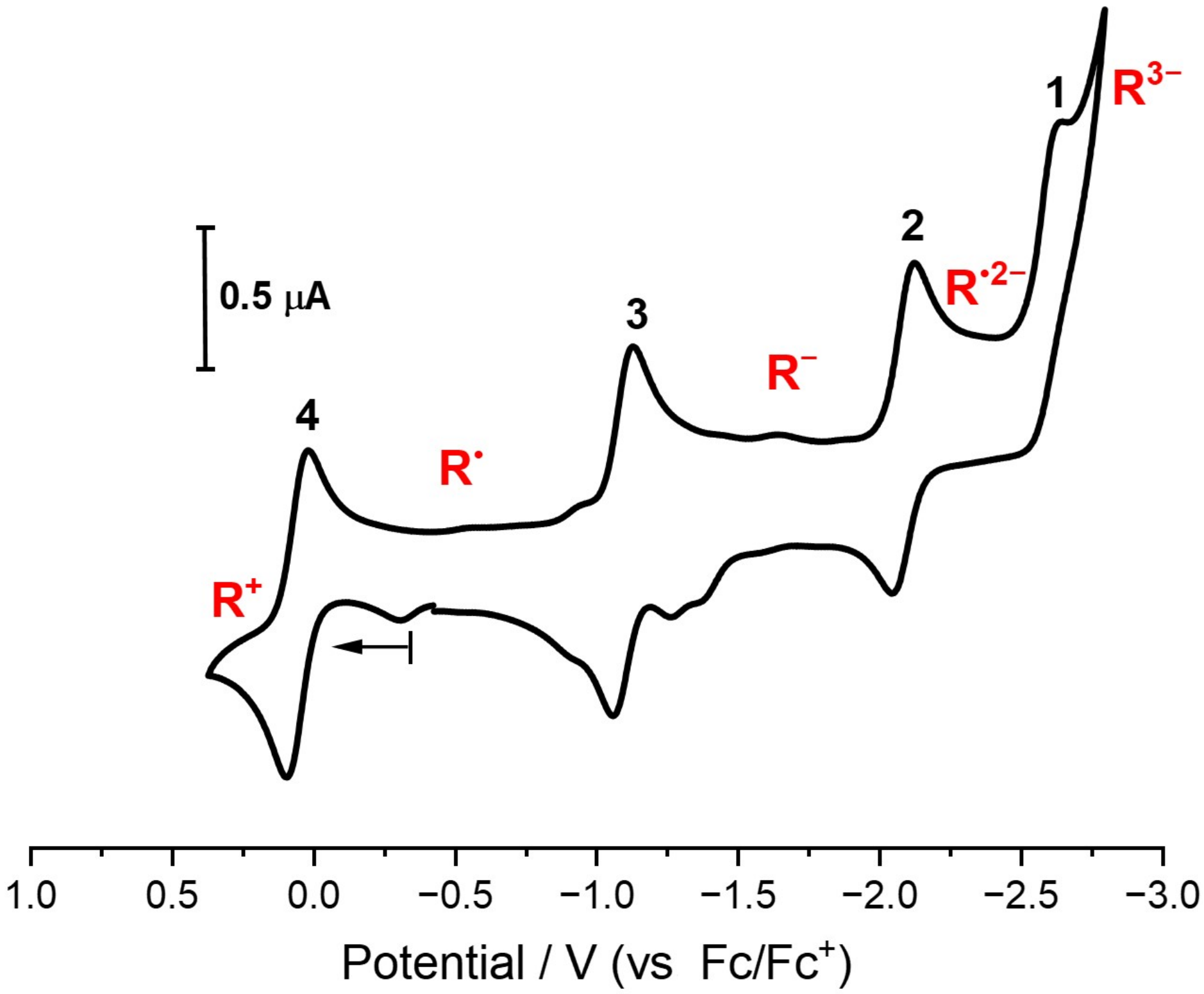

6. Computational details

Quantum-chemical calculations were performed using the methods recommended by Bursch et al. [9]. Radical **7** was optimized at the upbe0 [10-12]/def2-TZVP [13,14] level with Grimme's dispersion correction at the D3 level [15] and with Becke–Johnson damping (GD3BJ) [16] by using the Gaussian16 [17] software package. Except for the frequency analysis, all calculations were performed using the thus obtained geometries; all calculations were performed at this level (unless otherwise noted). Optimization and frequency analysis [18-20] at the uM06 [21]/6-311++g(d,p) [22-27] level verified that the structure is at a local minimum (no imaginary frequencies). IR spectra of the radicals were visualized with GaussSum [28] [full width at half maximum (FWHM) of 10 cm^{-1}] using a scaling factor of 0.9619 [29]. A UV–vis/NIR spectrum of the radical was calculated with a time-dependent DFT calculation [td=(nstates=50)] [30-32] applying a modelled solvent field of methylene chloride with the cpcm-scrf method [33-35] and visualized with GaussSum [full width at half maximum (FWHM) of 800 cm^{-1}]. Hyperfine coupling constants (EPR) and the *g* value were calculated with the keywords nmr=giao [36-39] and prop=epr [40-42]. Frontier orbitals and spin densities were determined by single-point energy calculation and visualized with GaussView [43]. NICS(1)$_{\pi zz}$ values [44] of **7** were calculated at the uB3LYP [45-47]/6-311g(d,p)level with the keyword nmr=giao. For the calculation of NICS(1)$_{\pi zz}$ values the program py.Aroma was used [48,49]. Output files for ACID calculations [50] were obtained using the keyword nmr=csgt. ACID calculations were performed with AICD 3.04 at the upbe0/def2-TZVP level. An ACID plot was visualized with the image rendering software POVRay [51].

7. Archive entry for a single-point energy calculation on a minimum structure of radical 7

Archive entry for radical **7**:


```
1\1\GINC-N1705\FOpt\UM06\6-311++G(d,p)\C72H61(2)\KA_LM9425\13-May-2026
\0\\# opt=tight uM06/6-311++g(d,p) geom=connectivity\\Title Card Requi
red\\0,2\C,0.0083485045,-0.0143920277,-0.0073818775\C,0.0090057013,0.0
054527053,1.433664413\C,1.1816338858,0.0139704562,2.1407389371\C,2.388
6795506,0.0071839366,1.4122728666\C,2.391308871,-0.0025313901,-0.02646
59997\C,1.2200967463,-0.0156498771,-0.7351290807\H,1.2010359983,0.0227
950745,3.2297233215\H,1.203174473,-0.0286090876,-1.8241579524\C,3.7223
535826,0.0046203687,1.8748355534\C,4.5815867701,-0.0037798862,0.754863
2523\C,5.9903555608,-0.0086512355,0.6992046471\C,6.5805695621,-0.00727
40485,-0.5363826974\C,5.7882938589,0.0038854678,-1.7402184262\C,4.3760
588292,0.003455397,-1.6813406763\C,3.7878906881,-0.0030597131,-0.44515
03513\H,6.5732974082,-0.010679438,1.6192902154\H,3.7911203868,0.009857
6117,-2.6001477447\C,7.9796844076,-0.0039996337,-0.9466425864\C,6.6430
079642,0.0211078914,-2.8505645159\C,-1.3850253038,0.0012964485,1.86082
15189\C,-1.3162097341,-0.0373509709,-0.4642608483\C,8.0003525166,0.013
8545603,-2.368563837\C,-2.1845245071,-0.0254537474,0.6849105109\C,-1.9
666800492,0.0152096473,3.1126344841\C,-3.5775658293,-0.039615831,0.761
8784091\C,9.1540835118,-0.0122398921,-0.2211946917\C,9.205959296,0.024
7016454,-3.0707840733\C,-3.3611012976,0.0023635557,3.1807087511\C,-4.1
536572661,-0.0248943263,2.0195832196\C,10.3559448712,-0.0027007117,-0.
9315779178\C,10.3789571964,0.0157475929,-2.3373744095\C,-4.2447041065,
0.0120402861,4.3387742068\C,-5.6266063857,-0.034577958,2.3756526441\C,
11.7309315194,-0.0081747846,-0.4506004676\C,11.8048416106,0.0235271291
,-2.8504429604\C,-5.5712106217,-0.0092140692,3.8890248421\C,12.5911867
104,0.0069154408,-1.5560775169\C,-3.9600453056,0.0372554962,5.69913934
71\C,-6.6169523755,-0.0053731807,4.7932992251\C,12.2416381559,-0.02492
29644,0.8421346163\C,13.9619414613,0.0053458535,-1.3762560669\H,-1.361
1700851,0.034985457,4.01636057\H,-4.1788340097,-0.0615237749,-0.146835
0915\H,9.1456391411,-0.0252869098,0.8666970583\H,9.2081282882,0.039684
6385,-4.1605425519\C,-5.0139887786,0.0409989053,6.6030172299\C,-6.3320
```

```
109426,0.0199016395,6.1561844482\C,13.619020824,-0.0264319654,1.017102
7118\C,14.4738046138,-0.0114513106,-0.0812671948\H,-2.9323913015,0.053
8255517,6.0526616355\H,-4.8098962566,0.0605992971,7.669351059\H,-7.143
6048729,0.0232592615,6.8775276572\H,-7.6496231586,-0.0217677098,4.4510
969373\H,11.5779924453,-0.0367094323,1.7028068066\H,14.0352483455,-0.0
394686435,2.0199429645\H,15.5483897269,-0.0129596914,0.0746135189\H,14
.6360089644,0.0169805383,-2.2302333457\C,-1.7605716105,-0.0886328709,-
1.8683109455\C,-1.9987362429,1.0980752345,-2.5737052342\C,-1.945516088
8,-1.3301795358,-2.4919197178\C,-2.420097338,1.0223773128,-3.897436042
4\C,-2.3672827826,-1.3626689172,-3.8165151588\C,-2.6126537404,-0.19771
02844,-4.5352444194\H,-2.6011129606,1.9452177103,-4.4469173678\H,-2.50
69134099,-2.3274752454,-4.3024628104\C,4.1460608386,0.010628549,3.2877
229774\C,4.3562251353,1.2289773498,3.947797584\C,4.3429086947,-1.20133
08407,3.9626021304\C,4.7604821073,1.213050424,5.2784054499\C,4.7471244
733,-1.1734354276,5.2935042727\C,4.958134197,0.0227449901,5.9694720995
\H,4.927880179,2.1598957614,5.7901302123\H,4.903814291,-2.115730438,5.
8167315438\C,6.2414555891,0.0632895525,-4.2677416554\C,6.0554797705,-1
.1278903407,-4.9811144956\C,6.0611482847,1.3009876436,-4.900423201 9\C,
5.6874463142,-1.0606000342,-6.3211561699\C,5.6935421145,1.3250563062,-
6.2411625804\C,5.498417481,0.1553772776,-6.9676957339\H,5.5463673228,-
1.9868809945,-6.8764999693\H,5.5573339748,2.2868764798,-6.7339700814\C
,-1.7920844593,2.4270784082,-1.9121737768\C,-1.684057476,-2.6019753627
,-1.7429626764\C,-3.098194807,-0.2582489379,-5.9511318728\H,-2.3943931
799,2.5183900614,-1.0008647565\H,-2.0617271436,3.2476405679,-2.5822176
22\H,-0.7469501043,2.5641752251,-1.6107871669\H,-0.635000462,-2.674199
9662,-1.433006763\H,-1.9167442178,-3.475922402,-2.3569937429\H,-2.2846
890631,-2.6580335494,-0.8276730443\H,-4.1917272128,-0.3298682559,-5.98
98406954\H,-2.811335695,0.6360791022,-6.5120000696\H,-2.6983849141,-1.
1318940411,-6.4746141427\C,5.362109286,0.0312798679,7.4119708194\C,4.1
523383623,2.5284298941,3.2289327624\C,4.1260355889,-2.5074008334,3.259
6804638\H,4.3585257656,3.3780198195,3.8850932705\H,4.806487194,2.60820
80625,2.3529625309\H,3.1242676654,2.6245568918,2.860868152\H,5.9920953
55,0.8945570585,7.6464337906\H,5.9124814098,-0.8753832262,7.6797467369
\H,4.483923546,0.083987618,8.0664960886\H,4.3175851118,-3.3508965481,3
.9280306819\H,3.0990070513,-2.5951150961,2.8867409754\H,4.7842454541,-
2.6071762634,2.3887709191\C,6.2625045828,2.5776535681,-4.1412626535\C,
6.2503445275,-2.4526461802,-4.3075921982\C,5.0708247613,0.2067753771,-
8.4024699688\H,5.5598394497,2.6584128026,-3.303678383\H,6.1199767394,3
.4473971532,-4.787935754\H,7.2691585504,2.6333557043,-3.7108846023\H,7
.2554610066,-2.5399264085,-3.8789180378\H,6.1069785416,-3.277559471,-5
.0104091542\H,5.5448409397,-2.5859954257,-3.4791111343\H,3.9793146519,
0.2703741559,-8.485789702\H,5.3867025171,-0.6875360117,-8.9475107956\H
,5.4852263715,1.0814328803,-8.9128496863\C,12.097621886,-1.2196944288,
-3.6906487314\C,12.0958133918,1.2890580242,-3.657315048\C,-6.336629744
7,1.2036840961,1.8280790612\C,-6.310141587,-1.3048171165,1.8691621084\
H,11.8914722058,2.1859053282,-3.0633399265\H,11.4737596136,1.324721069
8,-4.5589913313\H,13.1457550949,1.316422525,-3.9709688176\H,11.8939636
815,-2.1322999486,-3.1209410396\H,13.1477392964,-1.2375228503,-4.00439
66098\H,11.4760902876,-1.2319388267,-4.5933048294\H,-7.3593808691,-1.3
331971565,2.1850706087\H,-6.2866871046,-1.3466258992,0.7741966642\H,-5
.8103164271,-2.1981505571,2.2577769262\H,-7.3862045528,1.2205292648,2.
1436942713\H,-5.855553392,2.1196115739,2.1865933518\H,-6.3142107655,1.
2097873845,0.7323109783\\Version=ES64L-G16RevC.01\State=2-A\HF=-2778.9
946952\S2=1.001916\S2-1=0.\S2A=0.776247\RMSD=2.787e-09\RMSF=1.229e-07\
Dipole=-0.1631958,0.0008029,-0.3985131\Quadrupole=2.298997,-15.0952983
,12.7963014,0.3834752,3.6914556,0.0132713\PG=C01 [X(C72H61)]\\@
```

8. Frequency analyses and visualized IR spectra

Frequency analyses of radical **7** were carried out at the uM06/6-311++g(d,p) level; no imaginary frequencies were detected; lowest frequencies at $\tilde{\upsilon}$ = 7.54, 11.8, 11.9, 21.5, 24.4, 25.1 cm^{-1}, etc. for radical **7**. These are attributable to the torsion of the undecacycle along the molecule axis and various twisting and bending modes of the core and the mesityl group, and to rotations of the methyl groups.

Extract from the frequency calculation output for radical **7**:

```
Low frequencies ---  -10.4920   -0.0011   -0.0005    0.0007    2.6424    3.9968
Low frequencies ---   10.1362   11.8336   13.7327 […]
Harmonic frequencies (cm**-1), IR intensities (KM/Mole), Raman scattering
activities (A**4/AMU), depolarization ratios for plane and unpolarized
incident light, reduced masses (AMU), force constants (mDyne/A),
and normal coordinates:
                   1                      2                      3
                   A                      A                      A
Frequencies --      7.5446                11.8276                11.9299
Red. masses --      4.9358                 5.1539                 5.2981
Frc consts  --      0.0002                 0.0004                 0.0004
IR Inten    --      0.0240                 0.0054                 0.1371
[…]
                   4                      5                      6
                   A                      A                      A
Frequencies --     21.5256                24.4743                25.1147
Red. masses --      4.8943                 3.8362                 3.7366
Frc consts  --      0.0013                 0.0014                 0.0014
IR Inten    --      0.0073                 0.0003                 0.0103
[…]
                   7                      8                      9
                   A                      A                      A
Frequencies --     25.6517                25.9871                29.0423
Red. masses --      3.7849                 4.4982                 4.3314
Frc consts  --      0.0015                 0.0018                 0.0022
IR Inten    --      0.0127                 0.0355                 0.0649
[…]
                  10                     11                     12
                   A                      A                      A
Frequencies --     29.2311                29.7521                34.0797
Red. masses --      4.1663                 3.8513                 4.3551
Frc consts  --      0.0021                 0.0020                 0.0030
IR Inten    --      0.0002                 0.0129                 0.0057
[…]
                  13                     14                     15
                   A                      A                      A
Frequencies --     35.5575                45.2871                48.5393
Red. masses --      5.1454                 1.0257                 1.0201
Frc consts  --      0.0038                 0.0012                 0.0014
IR Inten    --      0.0598                 0.1377                 0.1656
```

Calculated IR spectrum of radical **7** (top spectrum) visualized with GaussSum: scaling factor of 0.9619; FWHM: 10 cm^{-1}. Measured and aligned IR spectra (bottom spectrum) for comparison.

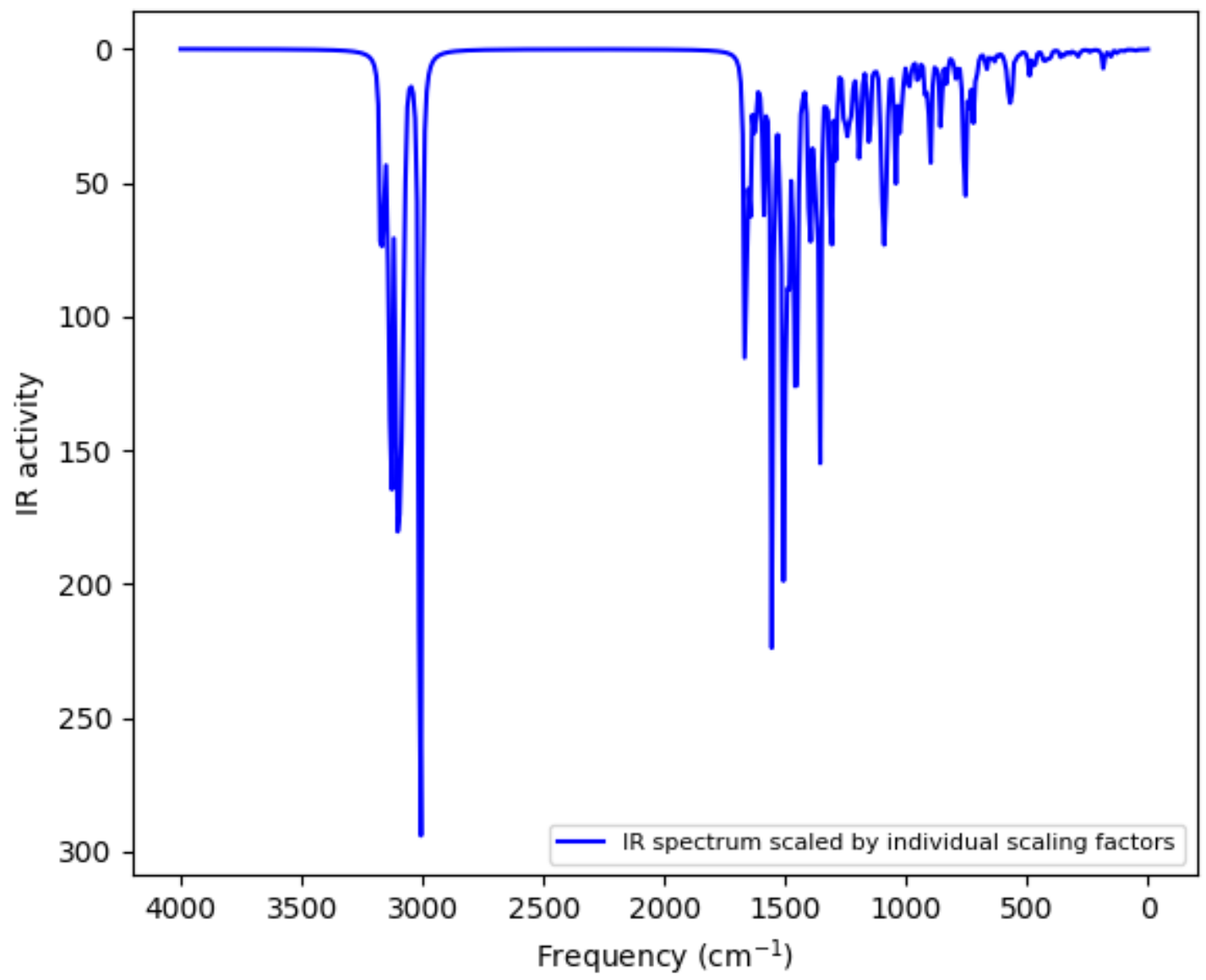

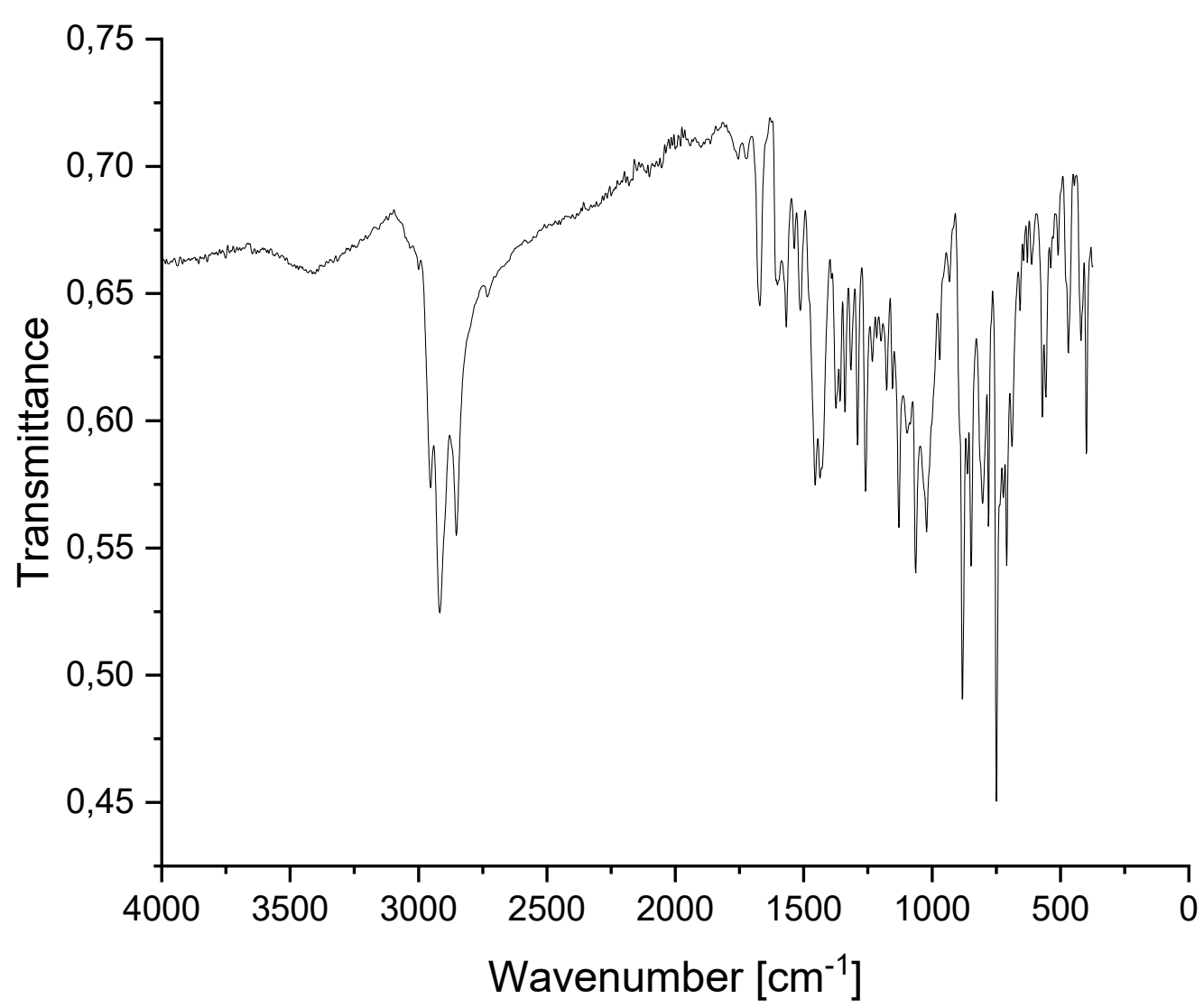

9. SOMOs of α and β electrons of radical 7

Magnified pictures of SOMOs of α (left) and β (right) electrons of radical **7**, calculated at the upbe0/def2tzvp (GD3BJ) level (isovalue: 0.02 electrons$^{1/2}$ bohr$^{-3/2}$).

–4.89 eV

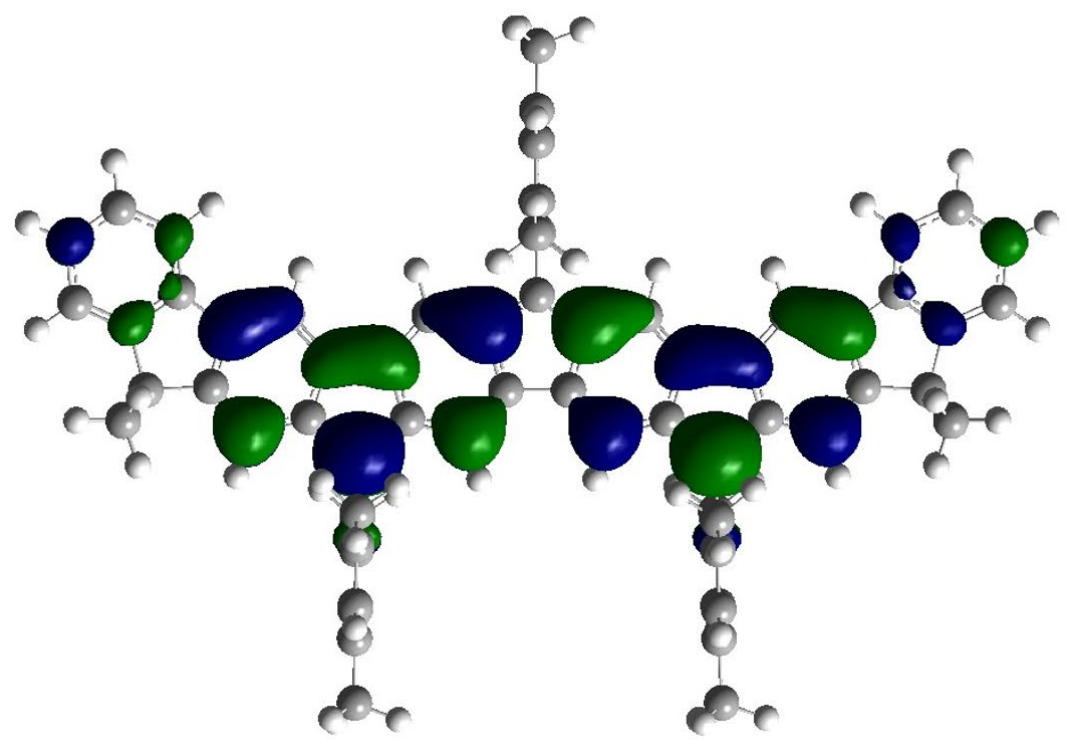

–3.11 eV

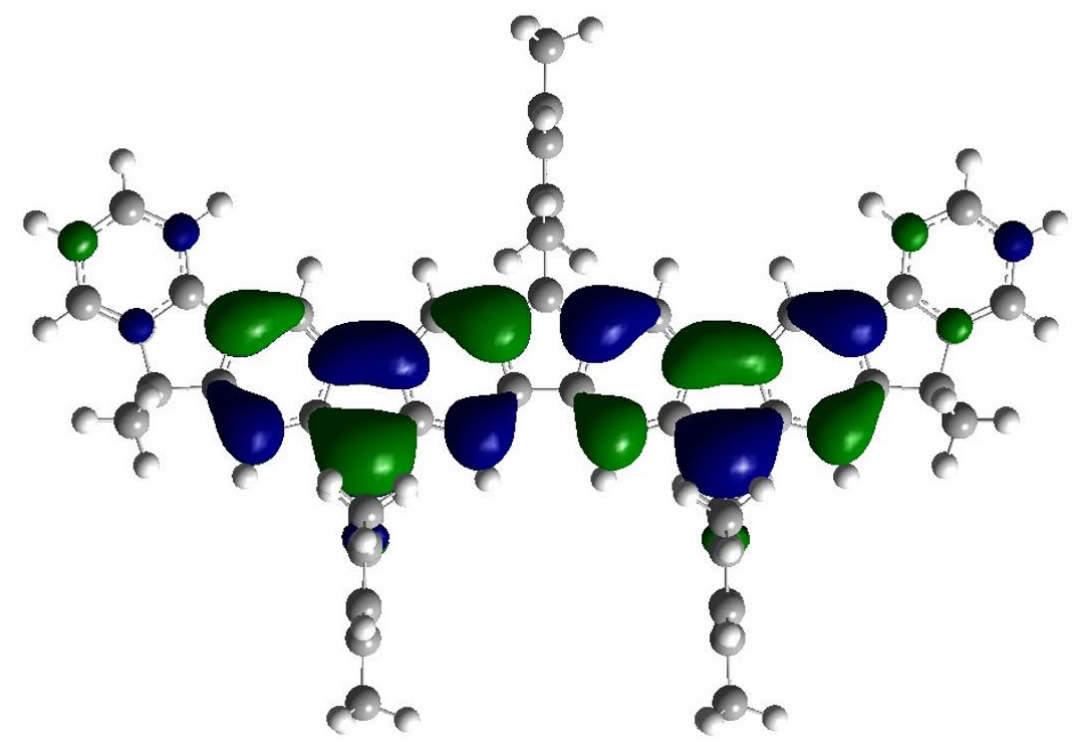

10. Calculated spin densities of radical 7

Calculated spin densities (magnified) of radical **7**; blue: α spin, green: β spin (isovalue: 0.004 electrons·bohr^{-3}), calculated at the upbe0/def2-TZVP/GD3BJ level.

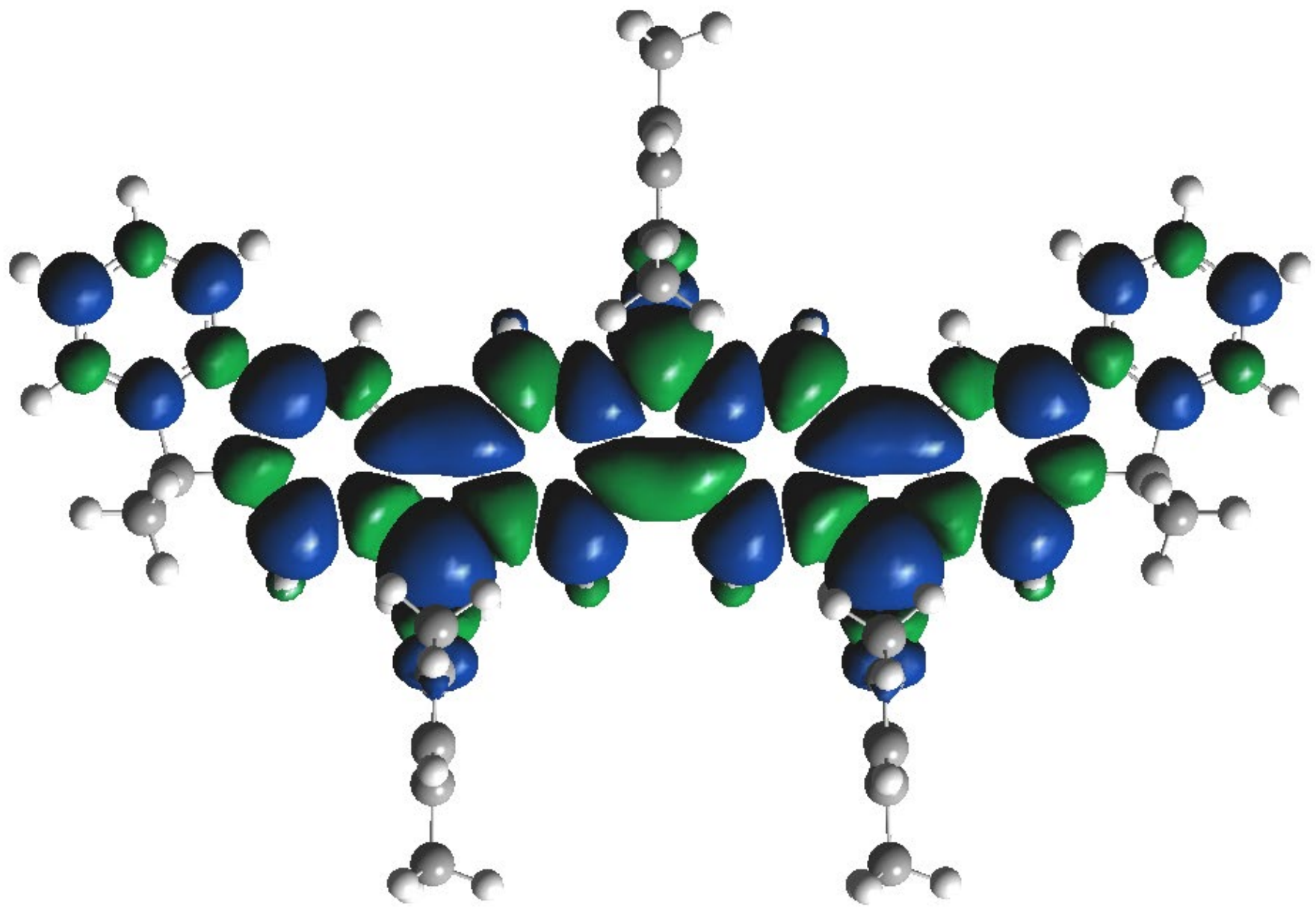

11. TD calculations

upbe0/def2tzvp (GD3BJ, CH_2Cl_2)

Excerpt of the output file of radical **7**, where only a selected number of transitions are given. Text after // was added as comment by the authors.

Radical **7**:

```
***********************************************************************
Excited states from <AA,BB:AA,BB> singles matrix:
***********************************************************************
Ground to excited state transition electric dipole moments (Au):
       state          X           Y           Z        Dip. S.      Osc.
         1         0.1634     -0.0001     -0.0029      0.0267      0.0008
         2        -0.0002      0.0270      0.0003      0.0007      0.0000
         3         4.8410      0.0004     -0.0047     23.4350      0.9808
         4        -0.0001      0.0134     -0.0000      0.0002      0.0000
         5        -2.7896     -0.0002      0.0005      7.7818      0.3592
         6         0.6864      0.0001      0.0085      0.4712      0.0243
         7        -1.0906     -0.0003     -0.0048      1.1894      0.0727
         8         0.0009     -0.3035     -0.0012      0.0921      0.0058
         9        -0.0005      0.5892     -0.0008      0.3472      0.0221
        10         0.4477      0.0011     -0.0068      0.2005      0.0128
[…]
Excitation energies and oscillator strengths:

Excited State   1:  2.021-A      1.2805 eV  968.25 nm  f=0.0008  <S**2>=0.771
247A -> 248A      -0.68696  // SOMO -> LUMO
245B -> 247B       0.69516  // HOMO-1 -> LUMO
246B -> 248B      -0.16040  // SOMO -> LUMO+1

Excited State   2:  2.301-A      1.3278 eV  933.76 nm  f=0.0000  <S**2>=1.074
245A -> 248A       0.10705  // HOMO-2 -> LUMO
244B -> 248B      -0.14099  // HOMO-2 -> LUMO+1
245B -> 248B      -0.12944  // HOMO-1 -> LUMO+1
246B -> 247B       0.96224  // SOMO -> LUMO

Excited State   3:  2.396-A      1.7083 eV  725.76 nm  f=0.9808  <S**2>=1.185
246A -> 248A       0.33661  // HOMO-1 -> LUMO
247A -> 248A       0.62551  // SOMO -> LUMO
244B -> 247B       0.21562  // HOMO-2 -> LUMO
245B -> 247B       0.56540  // HOMO-1 -> LUMO
246B -> 248B      -0.31705  // SOMO -> LUMO+1

Excited State   4:  3.224-A      1.8207 eV  680.98 nm  f=0.0000  <S**2>=2.348
244A -> 248A      -0.18439  // HOMO-3 -> LUMO
245A -> 248A       0.67632  // HOMO-2 -> LUMO
244B -> 248B       0.15196  // HOMO-2 -> LUMO+1
245B -> 248B      -0.64444  // HOMO-1 -> LUMO+1
246B -> 247B      -0.14255  // SOMO -> LUMO

Excited State   5:  3.051-A      1.8841 eV  658.07 nm  f=0.3592  <S**2>=2.077
246A -> 248A       0.72516  // HOMO-1 -> LUMO
247A -> 248A      -0.30027  // SOMO -> LUMO
247A -> 249A       0.15112  // SOMO -> LUMO+1
244B -> 247B       0.17445  // HOMO-2 -> LUMO
245B -> 247B      -0.36882  // HOMO-1 -> LUMO
246B -> 248B      -0.36998  // SOMO -> LUMO+1
```

```
Excited State   6:  2.247-A      2.1041 eV  589.24 nm  f=0.0243  <S**2>=1.012
246A -> 248A       0.54164  // HOMO-1 -> LUMO
246A -> 249A      -0.12087  // HOMO-1 -> LUMO+1
244B -> 247B      -0.48689  // HOMO-2 -> LUMO
245B -> 247B       0.16958  // HOMO-1 -> LUMO
246B -> 248B       0.59727  // SOMO -> LUMO+1

Excited State   7:  2.510-A      2.4937 eV  497.19 nm  f=0.0727  <S**2>=1.325
246A -> 248A       0.16028  // HOMO-1 -> LUMO
246A -> 249A       0.17081  // HOMO-1 -> LUMO+1
247A -> 248A      -0.14349  // SOMO -> LUMO
247A -> 249A      -0.30860  // SOMO -> LUMO+1
247A -> 251A       0.12952  // SOMO -> LUMO+3
243B -> 248B       0.16025  // HOMO-3 -> LUMO+1
244B -> 247B       0.65011  // HOMO-2 -> LUMO
246B -> 248B       0.50725  // SOMO -> LUMO+1
246B -> 249B      -0.13224  // SOMO -> LUMO+2

Excited State   8:  2.491-A      2.5707 eV  482.29 nm  f=0.0058  <S**2>=1.301
244A -> 248A      -0.18728  // HOMO-3 -> LUMO
245A -> 248A       0.30642  // HOMO-2 -> LUMO
245A -> 251A       0.13803  // HOMO-2 -> LUMO+3
246A -> 250A      -0.10410  // HOMO-1 -> LUMO+2
247A -> 250A       0.14420  // SOMO -> LUMO+2
243B -> 247B       0.69916  // HOMO-3 -> LUMO
244B -> 248B       0.25872  // HOMO-2 -> LUMO+1
245B -> 248B       0.37900  // HOMO-1 -> LUMO+1

Excited State   9:  2.539-A      2.6016 eV  476.56 nm  f=0.0221  <S**2>=1.361
244A -> 248A       0.35183  // HOMO-3 -> LUMO
245A -> 248A       0.61342  // HOMO-2 -> LUMO
247A -> 250A      -0.12555  // SOMO -> LUMO+2
243B -> 247B      -0.27108  // HOMO-3 -> LUMO
244B -> 248B      -0.18484  // HOMO-2 -> LUMO+1
245B -> 248B       0.52035  // HOMO-1 -> LUMO+1
245B -> 249B       0.13724  // HOMO-1 -> LUMO+2

Excited State  10:  2.423-A      2.6089 eV  475.23 nm  f=0.0128  <S**2>=1.217
245A -> 250A       0.13920  // HOMO-2 -> LUMO+2
246A -> 249A       0.13782  // HOMO-1 -> LUMO+1
247A -> 249A       0.89181  // SOMO -> LUMO+1
244B -> 247B       0.15394  // HOMO-2 -> LUMO
246B -> 249B      -0.12235  // SOMO -> LUMO+2
```

12. Calculated UV–vis spectra of radical 7

Calculated UV–vis spectrum of radical **7**, calculated at the upbedef2tzvp (GD3BJ, CH_2Cl_2) level; visualized with GaussSum: FWHM = 800 cm^{-1}.

UV–vis spectra of radical **7** with identical section and scaling allowing for comparison: measured (top) and calculated (bottom) spectra:

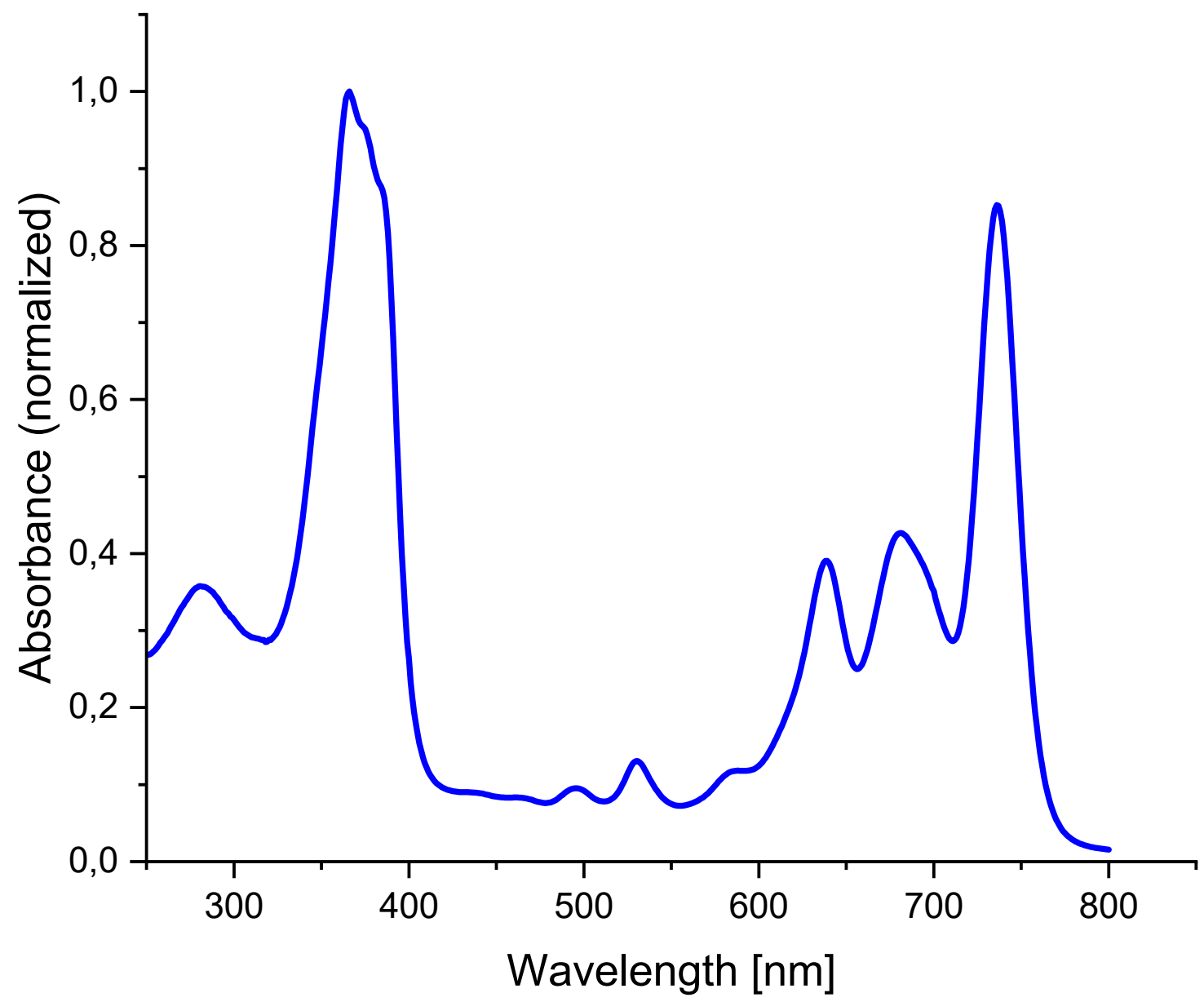

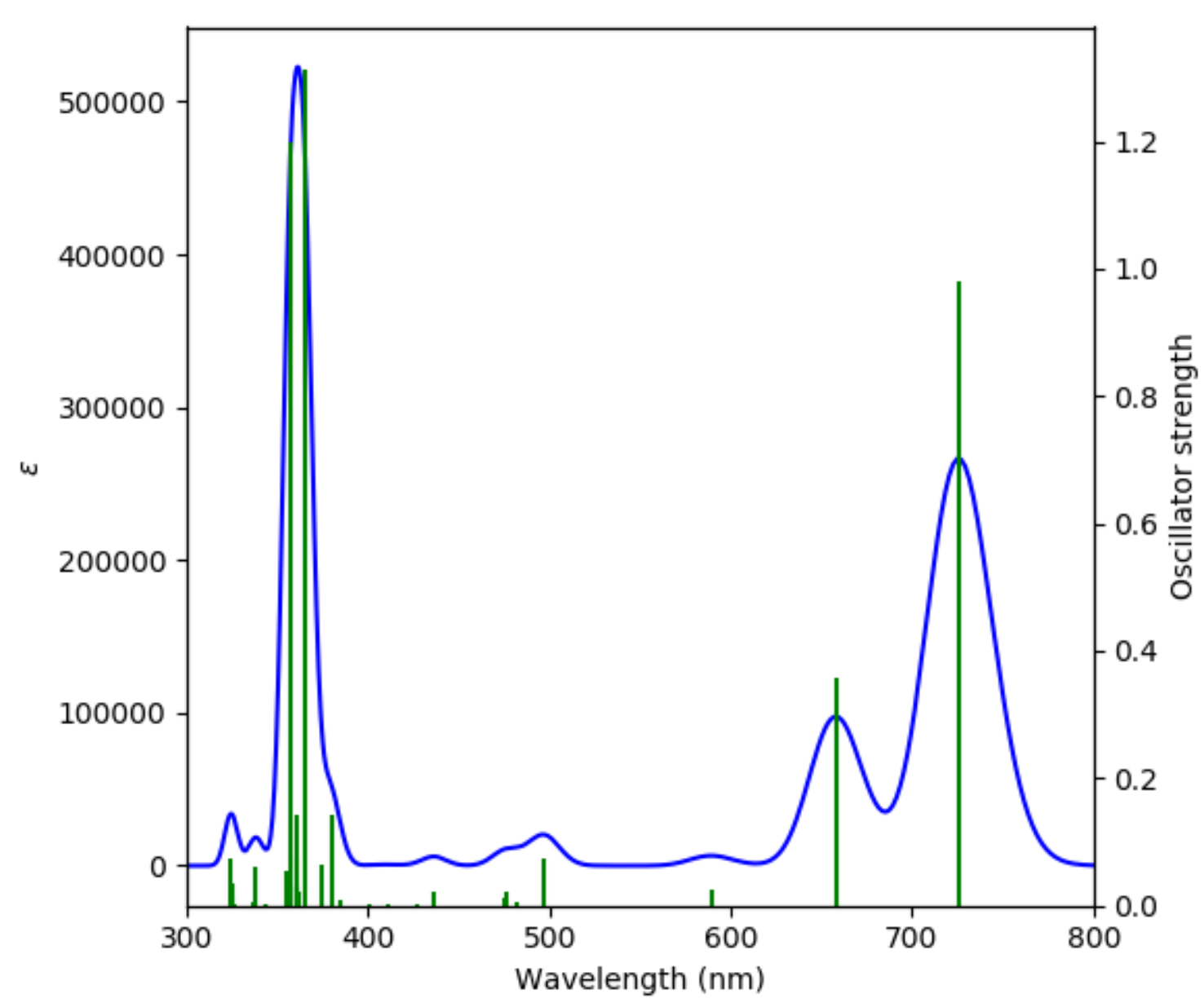

13. Calculated EPR data for radical 7

Calculated at the upbe0/def2tzvp (GD3BJ) level.

Radical **7**:

g value: $g_{xx} = 2.0023193 + 7.80 \cdot 10^{-6}$
$g_{yy} = 2.0023193 + 328.0 \cdot 10^{-6}$
$g_{zz} = 2.0023193 + 696.1 \cdot 10^{-6}$
$g_{iso} = (g_{xx} + g_{yy} + g_{zz}) / 3 = 2.0026$

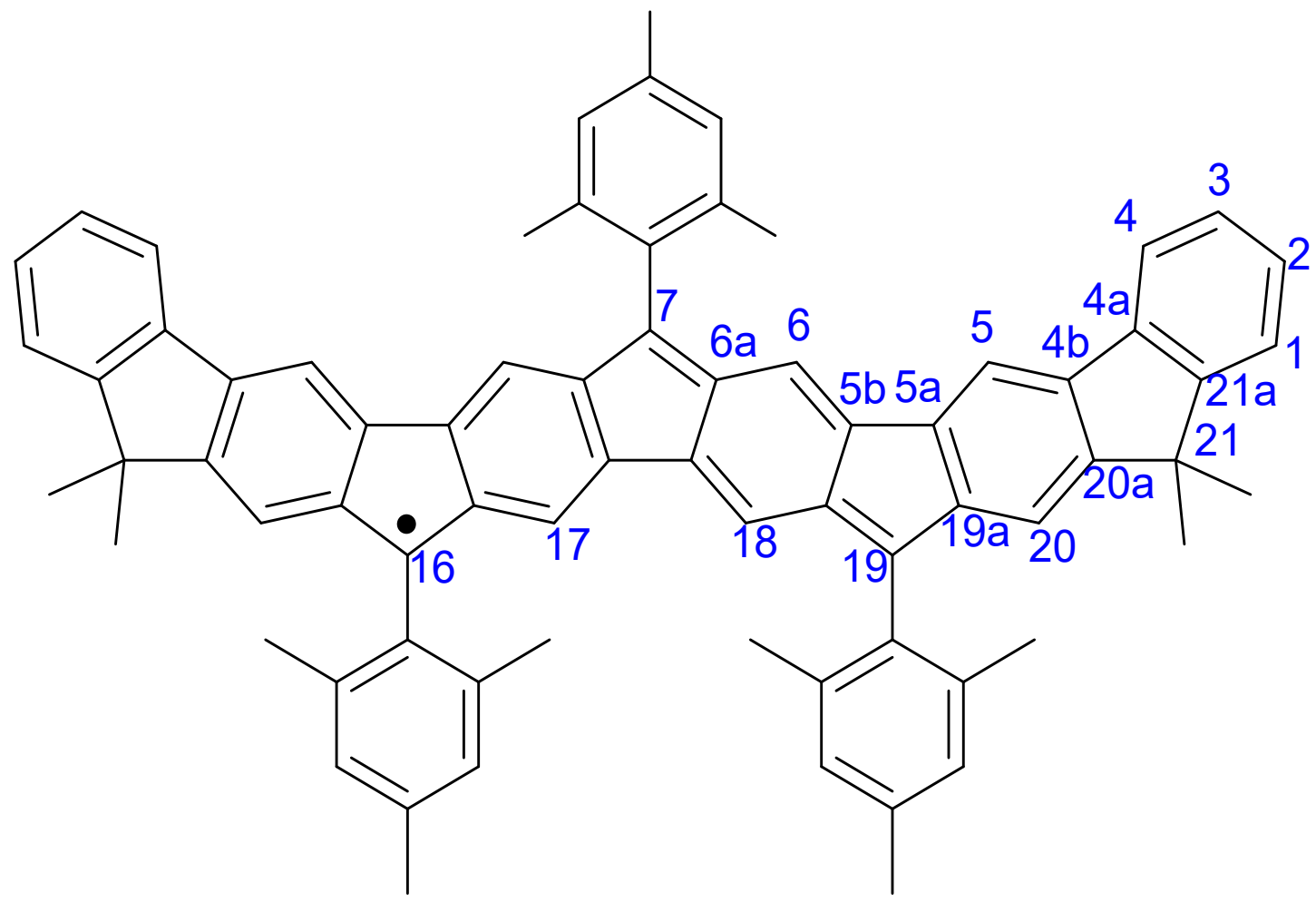

Output (excerpt; boldfacing and IUPAC numbering was given by the authors):

	Atom	Isotropic Fermi Contact Couplings a.u.	MegaHertz	Gauss	10(-4) cm-1	IUPAC
1	C(13)	-0.02344	-26.34946	-9.40214	-8.78923	
2	C(13)	0.01080	12.13950	4.33168	4.04930	
3	C(13)	-0.01330	-14.95073	-5.33479	-4.98703	
4	C(13)	0.02042	22.95121	8.18956	7.65570	
5	C(13)	-0.01009	-11.34109	-4.04678	-3.78298	
6	C(13)	0.01224	13.75462	4.90799	4.58805	//C-17
7	H(1)	0.00157	7.02490	2.50666	2.34325	
8	H(1)	-0.00196	-8.74115	-3.11906	-2.91573	
9	C(13)	-0.02492	**-28.01785**	-9.99747	-9.34575	//C-7
10	C(13)	0.02042	22.95673	8.19153	7.65754	//C-6a
11	C(13)	-0.01330	-14.95719	-5.33710	-4.98918	//C-6
12	C(13)	0.01080	12.14306	4.33295	4.05049	//C-5b
13	C(13)	-0.02344	-26.34720	-9.40134	-8.78848	
14	C(13)	0.01223	13.75015	4.90640	4.58656	//C-18
15	C(13)	-0.01009	-11.33945	-4.04620	-3.78243	
16	H(1)	0.00157	7.03052	2.50866	2.34513	
17	H(1)	-0.00195	-8.73791	-3.11790	-2.91465	
18	C(13)	0.00286	3.21241	1.14627	1.07154	//C-5a
19	C(13)	0.03036	**34.13581**	12.18051	11.38648	//C-19
20	C(13)	0.00286	3.21489	1.14715	1.07237	
21	C(13)	0.03037	**34.13635**	12.18070	11.38666	//C-16
22	C(13)	-0.01812	-20.37395	-7.26993	-6.79602	//C-19a
23	C(13)	-0.01812	-20.37490	-7.27027	-6.79633	
24	C(13)	-0.00612	-6.88271	-2.45592	-2.29583	

25	C(13)	0.00788	8.85652	3.16022	2.95422	
26	C(13)	-0.00612	-6.88037	-2.45509	-2.29504	//C-5
27	C(13)	0.00788	8.85670	3.16029	2.95428	//C-20
28	C(13)	0.00745	8.37106	2.98700	2.79229	
29	C(13)	-0.00792	-8.90024	-3.17583	-2.96880	
30	C(13)	0.00745	8.37088	2.98694	2.79222	//C-4b
31	C(13)	-0.00792	-8.90089	-3.17606	-2.96902	//C-20a
32	C(13)	-0.00503	-5.65246	-2.01694	-1.88546	
33	C(13)	0.00021	0.23953	0.08547	0.07990	
34	C(13)	-0.00503	-5.65219	-2.01684	-1.88537	//C-4a
35	C(13)	0.00021	0.23979	0.08556	0.07998	//C-21
36	C(13)	0.00215	2.41478	0.86165	0.80548	
37	C(13)	0.00215	2.41504	0.86175	0.80557	//C-21a
38	C(13)	0.00228	2.56710	0.91601	0.85629	
39	C(13)	-0.00220	-2.47772	-0.88411	-0.82648	
40	C(13)	0.00228	2.56697	0.91596	0.85625	//C-4
41	C(13)	-0.00220	-2.47810	-0.88425	-0.82660	//C-1
42	H(1)	0.00035	1.54401	0.55094	0.51503	
43	H(1)	-0.00163	-7.29362	-2.60254	-2.43289	
44	H(1)	0.00035	1.54303	0.55059	0.51470	
45	H(1)	-0.00163	-7.29459	-2.60289	-2.43321	
46	C(13)	-0.00226	-2.54202	-0.90706	-0.84793	
47	C(13)	0.00229	2.57327	0.91821	0.85835	
48	C(13)	-0.00226	-2.54218	-0.90711	-0.84798	//C-3
49	C(13)	0.00229	2.57360	0.91832	0.85846	//C-2
50	H(1)	-0.00042	-1.86117	-0.66411	-0.62082	
51	H(1)	0.00019	0.86978	0.31036	0.29013	
52	H(1)	-0.00047	-2.08337	-0.74340	-0.69494	
53	H(1)	0.00020	0.89550	0.31954	0.29871	
54	H(1)	-0.00042	-1.86132	-0.66416	-0.62087	
55	H(1)	0.00019	0.86983	0.31038	0.29015	
56	H(1)	-0.00047	-2.08363	-0.74349	-0.69503	
57	H(1)	0.00020	0.89566	0.31960	0.29876	
58	C(13)	-0.01319	-14.82606	-5.29031	-4.94544	
59	C(13)	0.01536	17.27275	6.16335	5.76157	
60	C(13)	0.01557	17.50121	6.24487	5.83777	
61	C(13)	0.00108	1.21915	0.43502	0.40666	
62	C(13)	0.00118	1.32403	0.47245	0.44165	
63	C(13)	0.00011	0.12170	0.04343	0.04059	
64	H(1)	0.00040	1.77174	0.63220	0.59099	
65	H(1)	0.00040	1.80210	0.64303	0.60112	
66	C(13)	0.00855	9.61306	3.43018	3.20657	
67	C(13)	-0.00997	-11.20908	-3.99968	-3.73895	
68	C(13)	-0.00996	-11.20172	-3.99705	-3.73649	
69	C(13)	-0.00067	-0.75846	-0.27064	-0.25300	
70	C(13)	-0.00067	-0.75517	-0.26946	-0.25190	
71	C(13)	-0.00011	-0.12197	-0.04352	-0.04068	
72	H(1)	-0.00025	-1.13979	-0.40671	-0.38019	
73	H(1)	-0.00025	-1.13704	-0.40573	-0.37928	
74	C(13)	-0.01319	-14.82412	-5.28962	-4.94480	
75	C(13)	0.01537	17.27465	6.16402	5.76220	
76	C(13)	0.01557	17.50282	6.24544	5.83831	
77	C(13)	0.00109	1.22209	0.43607	0.40765	
78	C(13)	0.00118	1.32851	0.47405	0.44314	
79	C(13)	0.00011	0.12166	0.04341	0.04058	
80	H(1)	0.00040	1.77213	0.63234	0.59112	
81	H(1)	0.00040	1.80301	0.64336	0.60142	
82	C(13)	-0.00001	-0.01302	-0.00464	-0.00434	
83	C(13)	-0.00004	-0.04968	-0.01773	-0.01657	
84	C(13)	-0.00011	-0.12291	-0.04386	-0.04100	

85	H(1)	0.00020	0.88627	0.31624	0.29563
86	H(1)	0.00014	0.64533	0.23027	0.21526
87	H(1)	0.00015	0.67028	0.23917	0.22358
88	H(1)	0.00015	0.69043	0.24636	0.23030
89	H(1)	0.00015	0.65131	0.23240	0.21725
90	H(1)	0.00019	0.86641	0.30916	0.28900
91	H(1)	0.00006	0.25253	0.09011	0.08423
92	H(1)	0.00002	0.08475	0.03024	0.02827
93	H(1)	0.00002	0.10661	0.03804	0.03556
94	C(13)	0.00009	0.09598	0.03425	0.03201
95	C(13)	0.00003	0.02914	0.01040	0.00972
96	C(13)	0.00003	0.02889	0.01031	0.00964
97	H(1)	-0.00010	-0.46576	-0.16619	-0.15536
98	H(1)	-0.00010	-0.45195	-0.16127	-0.15075
99	H(1)	-0.00010	-0.45231	-0.16140	-0.15088
100	H(1)	-0.00002	-0.08368	-0.02986	-0.02791
101	H(1)	-0.00002	-0.06958	-0.02483	-0.02321
102	H(1)	-0.00005	-0.22047	-0.07867	-0.07354
103	H(1)	-0.00010	-0.46525	-0.16601	-0.15519
104	H(1)	-0.00010	-0.45652	-0.16290	-0.15228
105	H(1)	-0.00010	-0.44849	-0.16003	-0.14960
106	C(13)	-0.00004	-0.04909	-0.01752	-0.01637
107	C(13)	-0.00001	-0.01495	-0.00533	-0.00499
108	C(13)	-0.00011	-0.12322	-0.04397	-0.04110
109	H(1)	0.00015	0.68810	0.24553	0.22953
110	H(1)	0.00015	0.65439	0.23350	0.21828
111	H(1)	0.00020	0.87607	0.31260	0.29223
112	H(1)	0.00020	0.87788	0.31325	0.29283
113	H(1)	0.00014	0.64605	0.23053	0.21550
114	H(1)	0.00015	0.67972	0.24254	0.22673
115	H(1)	0.00006	0.25274	0.09018	0.08430
116	H(1)	0.00002	0.08719	0.03111	0.02908
117	H(1)	0.00002	0.10515	0.03752	0.03507
118	C(13)	0.00062	0.70006	0.24980	0.23351
119	C(13)	0.00062	0.70152	0.25032	0.23400
120	C(13)	0.00062	0.70163	0.25036	0.23404
121	C(13)	0.00063	0.70276	0.25076	0.23441
122	H(1)	-0.00000	-0.01446	-0.00516	-0.00482
123	H(1)	0.00003	0.13558	0.04838	0.04523
124	H(1)	-0.00008	-0.37386	-0.13340	-0.12471
125	H(1)	-0.00000	-0.01449	-0.00517	-0.00483
126	H(1)	-0.00008	-0.37337	-0.13323	-0.12454
127	H(1)	0.00003	0.13561	0.04839	0.04523
128	H(1)	-0.00008	-0.37340	-0.13324	-0.12455
129	H(1)	0.00003	0.13553	0.04836	0.04521
130	H(1)	-0.00000	-0.01446	-0.00516	-0.00482
131	H(1)	-0.00008	-0.37326	-0.13319	-0.12451
132	H(1)	-0.00000	-0.01455	-0.00519	-0.00485
133	H(1)	0.00003	0.13555	0.04837	0.04521

14. Triradical character

Structure optimized at the uM06/6-31g(d,p) level:

Input for radical **7**:

```
%mem=324gb
%nprocshared=48
#p uhf/def2tzvp guess=mix scf=(qc,tight) pop=no stable

Title

0 2
// Coordinates for the uM06/6-311++g(d,p)-optimized structure
```

Output (excerpt):

```
Natural Orbital Coefficients:
                        246       247       248       249       250
     Eigenvalues --     1.44093   1.00000   0.55907   0.28013   0.20296
```

Triradical character *y* was calculated using Yamaguchi's scheme.

$$n_{HONO} = 1.44093;\ n_{LUNO} = 0.55907$$

$$T = \frac{n_{HONO} - n_{LUNO}}{2}$$

$$y = 1 - \frac{2T}{1 + T^2}$$

$$y = 0.26$$

15. NICS-XY-scan of radical 7 (calculated without methyl and mesityl groups)

uB3LYP/6-311(d,p) nmr=giao integral=(grid=ultrafine) cphf=(grid=fine)

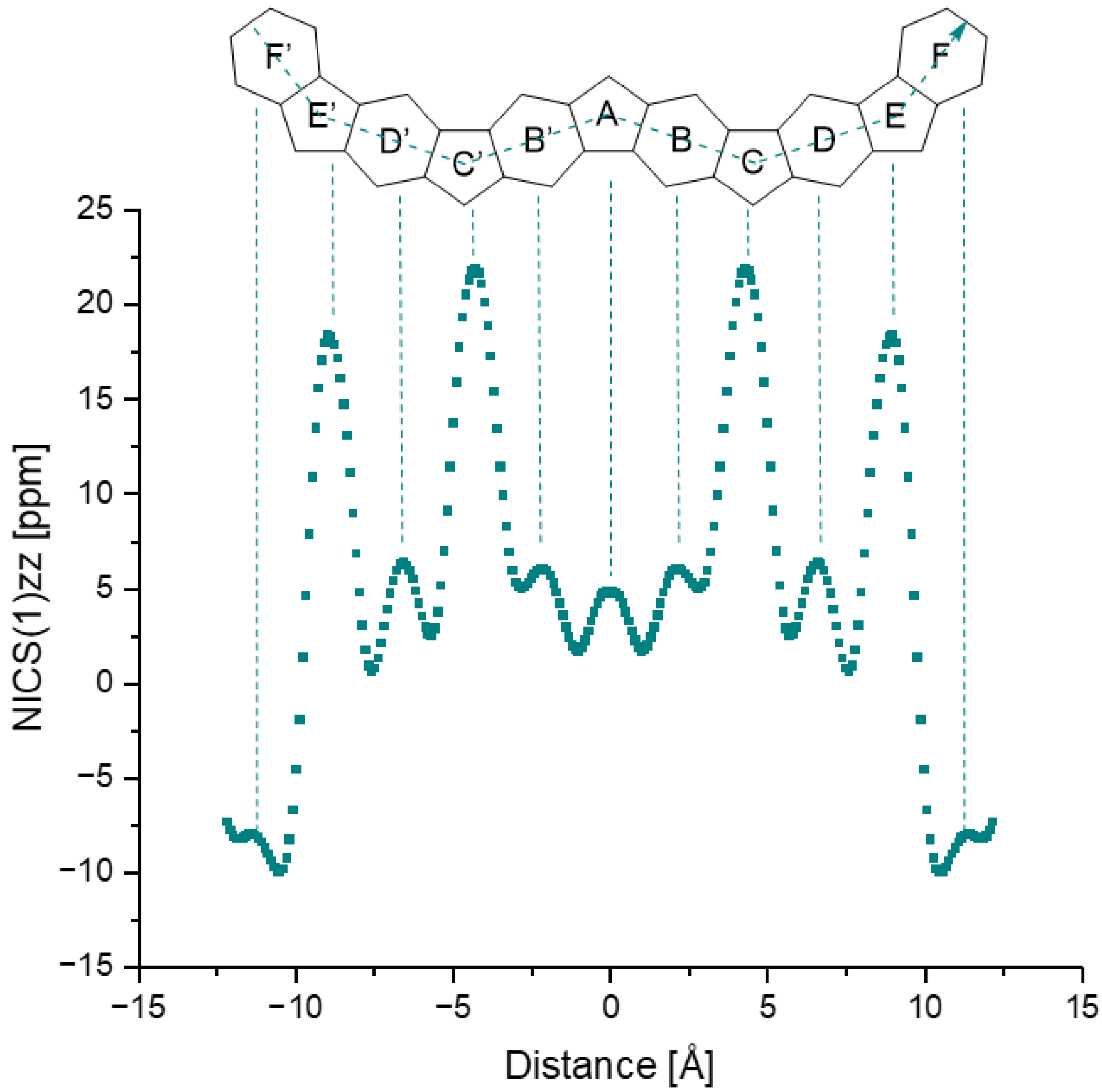

r	ZZ	Sigma-ZZ	Del-ZZ
0.00	-20.8470	-13.5079	-7.3391
0.10	-20.9039	-13.1224	-7.7815
0.20	-20.1202	-12.0580	-8.0622
0.30	-18.6551	-10.4637	-8.1914
0.40	-16.7417	-8.5391	-8.2026
0.50	-14.6338	-6.4929	-8.1409
0.60	-12.5616	-4.5063	-8.0553
0.70	-10.7024	-2.7147	-7.9877
0.80	-9.1733	-1.2026	-7.9707
0.90	-8.0367	-0.0113	-8.0254
1.00	-7.3138	0.8479	-8.1617
1.10	-7.0000	1.3787	-8.3787
1.20	-7.0889	1.5916	-8.6805
1.30	-7.5460	1.4733	-9.0193
1.40	-8.3344	1.0400	-9.3744
1.50	-9.4105	0.2894	-9.6999
1.60	-10.7098	-0.7750	-9.9348
1.70	-12.1343	-2.1330	-10.0013

1.80	-13.5412	-3.7344	-9.8068
1.90	-14.7376	-5.4886	-9.2490
2.00	-15.4900	-7.2603	-8.2297
2.10	-15.5505	-8.8764	-6.6741
2.20	-14.7024	-10.1476	-4.5548
2.30	-12.8139	-10.9007	-1.9132
2.41	-9.6295	-10.9971	1.3676
2.51	-5.7506	-10.3876	4.6370
2.61	-1.2792	-9.1618	7.8826
2.71	3.4295	-7.4582	10.8877
2.81	8.0137	-5.4650	13.4787
2.91	12.1604	-3.3860	15.5464
3.01	15.6329	-1.4138	17.0467
3.11	18.2741	0.2901	17.9840
3.21	19.9941	1.5997	18.3944
3.31	20.7500	2.4252	18.3248
3.41	20.5724	2.7088	17.8636
3.51	19.5915	2.4265	17.1650
3.61	17.7166	1.5984	16.1182
3.71	15.0205	0.2827	14.7378
3.81	11.6219	-1.4304	13.0523
3.91	7.6963	-3.4158	11.1121
4.01	3.4901	-5.5142	9.0043
4.11	-0.6833	-7.5365	6.8532
4.21	-4.4667	-9.2806	4.8139
4.31	-7.5053	-10.5591	3.0538
4.40	-9.4102	-11.2051	1.7949
4.50	-10.3213	-11.2534	0.9321
4.60	-10.0430	-10.6487	0.6057
4.70	-8.7243	-9.4911	0.7668
4.80	-6.6295	-7.9391	1.3096
4.90	-4.0738	-6.1759	2.1021
5.00	-1.3656	-4.3766	3.0110
5.10	1.2358	-2.6866	3.9224
5.20	3.5391	-1.2111	4.7502
5.30	5.4172	-0.0174	5.4346
5.40	6.7973	0.8576	5.9397
5.50	7.6419	1.3957	6.2462
5.60	7.9359	1.5885	6.3474
5.70	7.6715	1.4267	6.2448
5.80	6.8606	0.9082	5.9524
5.90	5.5234	0.0298	5.4936
6.00	3.7017	-1.2012	4.9029
6.10	1.4755	-2.7581	4.2336
6.20	-1.0234	-4.5818	3.5584
6.30	-3.5987	-6.5707	2.9720
6.40	-5.9904	-8.5785	2.5881
6.50	-7.8944	-10.4222	2.5278
6.60	-9.0045	-11.9041	2.8996
6.70	-9.0729	-12.8466	3.7737
6.80	-7.9826	-13.1291	5.1465
6.90	-5.7466	-12.7188	6.9722
7.00	-2.5479	-11.6695	9.1216
7.10	1.3111	-10.1186	11.4297
7.20	5.4726	-8.2551	13.7277
7.30	9.5857	-6.2844	15.8701
7.40	13.3497	-4.3992	17.7489
7.50	16.5367	-2.7630	19.2997
7.60	18.9886	-1.5032	20.4918
7.70	20.6029	-0.7121	21.3150
7.79	21.3125	-0.4489	21.7614
7.89	21.1753	-0.7211	21.8964
7.99	20.1416	-1.5286	21.6702
8.09	18.2702	-2.8120	21.0822
8.19	15.6607	-4.4748	20.1355

8.29	12.4636	-6.3804	18.8440
8.39	8.8893	-8.3554	17.2447
8.49	5.2090	-10.1979	15.4069
8.59	1.7343	-11.6974	13.4317
8.69	-1.2181	-12.6667	11.4486
8.78	-3.0507	-12.9722	9.9215
8.88	-4.4413	-12.7089	8.2676
8.98	-4.8407	-11.7753	6.9346
9.08	-4.3253	-10.2898	5.9645
9.18	-3.0784	-8.4282	5.3498
9.28	-1.3417	-6.3875	5.0458
9.38	0.6341	-4.3513	4.9854
9.48	2.6270	-2.4669	5.0939
9.58	4.4639	-0.8373	5.3012
9.68	6.0227	0.4766	5.5461
9.78	7.2251	1.4461	5.7790
9.88	8.0236	2.0627	5.9609
9.98	8.3954	2.3318	6.0636
10.08	8.2890	2.2329	6.0561
10.18	7.7184	1.7880	5.9304
10.28	6.6791	0.9997	5.6794
10.38	5.1819	-0.1226	5.3045
10.48	3.2641	-1.5528	4.8169
10.58	1.0041	-3.2366	4.2407
10.68	-1.4665	-5.0809	3.6144
10.78	-3.9590	-6.9491	2.9901
10.88	-6.2373	-8.6683	2.4310
10.98	-8.0471	-10.0492	2.0021
11.08	-9.1605	-10.9182	1.7577
11.18	-9.4281	-11.1547	1.7266
11.28	-8.8212	-10.7287	1.9075
11.38	-7.4108	-9.6752	2.2644
11.48	-5.3844	-8.1235	2.7391
11.58	-2.9930	-6.2559	3.2629
11.68	-0.5031	-4.2747	3.7716
11.78	1.8412	-2.3719	4.2131
11.88	3.8401	-0.7108	4.5509
11.98	5.3436	0.5821	4.7615
12.08	6.2481	1.4193	4.8288
12.18	6.4917	1.7485	4.7432

16. ACID Plot of radical 7 (calculated without methyl and mesityl groups)

nmr=csgt upbe0/def2tzvp (GD3BJ) iop(10/93=2); isosurface value of 0.02

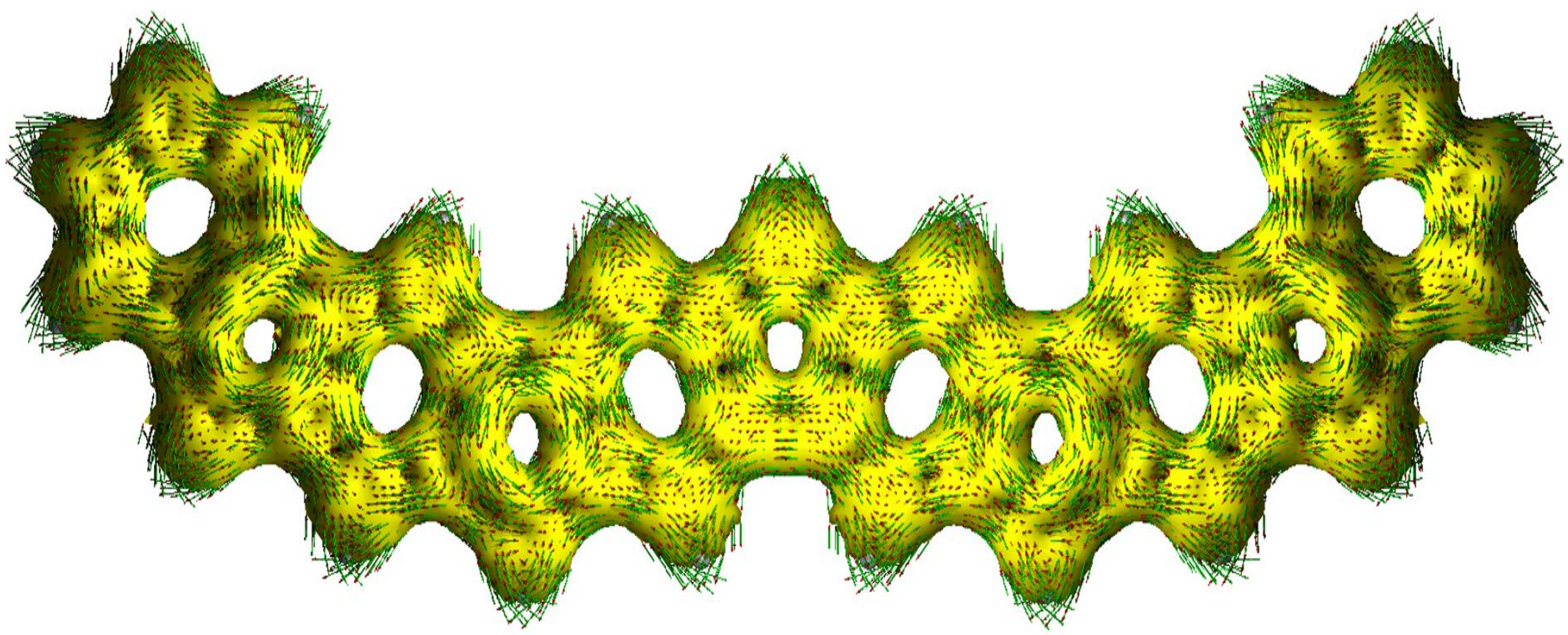

17. References

[1] Acan, A. S.; Wenzel, J. O.; Breher, F.; Podlech, J., *Chem. Eur. J.* **2025**, *31*, e202403670, doi: 10.1002/chem.202403670.

[2] Fulmer, G. R.; Miller, A. J. M.; Sherden, N. H.; Gottlieb, H. E.; Nudelman, A.; Stoltz, B. M.; Bercaw, J. E.; Goldberg, K. I., *Organometallics* **2010**, *29*, 2176-2179, doi: 10.1021/om100106e.

[3] van Gerven, L.; Talpe, J.; van Itterbeek, A., *Physica* **1967**, *33*, 207-211, doi: 10.1016/0031-8914(67)90273-X.

[4] Stoll, S.; Schweiger, A., *J. Magn. Reson.* **2006**, *178*, 42-55, doi: 10.1016/j.jmr.2005.08.013.

[5] Wei, J.-J.; Yang, Y.-J.; Liu, X.-Y.; Li, R.; Li, S.-a., *Chem. Eur. J.* **2022**, *28*, e202200756, doi: 10.1002/chem.202200756.

[6] Majewski, M. A.; Lis, T.; Cybińska, J.; Stępień, M., *Chem. Commun.* **2015**, *51*, 15094-15097, doi: 10.1039/C5CC06046J.

[7] Sharma, H.; Sharma, P. K.; Das, S., *Chem. Commun.* **2020**, *56*, 11319-11322, doi: 10.1039/D0CC04418K.

[8] Yang, X.; Zhang, D.; Liao, Y.; Zhao, D., *J. Org. Chem.* **2020**, *85*, 5761-5770, doi: 10.1021/acs.joc.9b03077.

[9] Bursch, M.; Mewes, J.-M.; Hansen, A.; Grimme, S., *Angew. Chem. Int. Ed.* **2022**, *61*, e202205735, doi: 10.1002/anie.202205735.

[10] Perdew, J. P.; Burke, K.; Ernzerhof, M., *Phys. Rev. Lett.* **1996**, *77*, 3865-3868, doi: 10.1103/PhysRevLett.77.3865.

[11] Perdew, J. P.; Burke, K.; Ernzerhof, M., *Phys. Rev. Lett.* **1997**, *78*, 1396, doi: 10.1103/PhysRevLett.78.1396.

[12] Adamo, C.; Barone, V., *J. Chem. Phys.* **1999**, *110*, 6158-6170, doi: 10.1063/1.478522.

[13] Weigend, F.; Ahlrichs, R., *Phys. Chem. Chem. Phys.* **2005**, *7*, 3297-3305, doi: 10.1039/b508541a.

[14] Weigend, F., *Phys. Chem. Chem. Phys.* **2006**, *8*, 1057-1065, doi: 10.1039/b515623h.

[15] Grimme, S.; Antony, J.; Ehrlich, S.; Krieg, H., *J. Chem. Phys.* **2010**, *132*, 154104, doi: 10.1063/1.3382344.

[16] Grimme, S.; Ehrlich, S.; Goerigk, L., *J. Comput. Chem.* **2011**, *32*, 1456-1465, doi: 10.1002/jcc.21759.

[17] Frisch, M. J.; Trucks, G. W.; Schlegel, H. B.; Scuseria, G. E.; Robb, M. A.; Cheeseman, J. R.; Scalmani, G.; Barone, V.; Petersson, G. A.; Nakatsuji, H.; Li, X.; Caricato, M.; Marenich, A. V.; Bloino, J.; Janesko, B. G.; Gomperts, R.; Mennucci, B.; Hratchian, H. P.; Ortiz, J. V.; Izmaylov, A. F.; Sonnenberg, J. L.; Williams; Ding, F.; Lipparini, F.; Egidi, F.; Goings, J.; Peng, B.; Petrone, A.; Henderson, T.; Ranasinghe, D.; Zakrzewski, V. G.; Gao, J.; Rega, N.; Zheng, G.; Liang, W.; Hada, M.; Ehara, M.; Toyota, K.; Fukuda, R.; Hasegawa, J.; Ishida, M.; Nakajima, T.; Honda, Y.; Kitao, O.; Nakai, H.; Vreven, T.; Throssell, K.; Montgomery Jr., J. A.; Peralta, J. E.; Ogliaro, F.; Bearpark, M. J.; Heyd, J. J.; Brothers, E. N.; Kudin, K. N.; Staroverov, V. N.; Keith, T. A.; Kobayashi, R.; Normand, J.; Raghavachari, K.; Rendell, A. P.; Burant, J. C.; Iyengar, S. S.; Tomasi, J.; Cossi, M.; Millam, J. M.; Klene, M.; Adamo, C.; Cammi, R.; Ochterski, J. W.; Martin, R. L.; Morokuma, K.; Farkas, O.; Foresman, J. B.; Fox, D. J. *Gaussian 16, Revision C.01*; Gaussian, Inc.: Wallingford, CT, 2016.

[18] Johnson, B. G.; Frisch, M. J., *Chem. Phys. Lett.* **1993**, *216*, 133-140, doi: 10.1016/0009-2614(93)E1238-C.

[19] Johnson, B. G.; Fisch, M. J., *J. Chem. Phys.* **1994**, *100*, 7429-7442, doi: 10.1063/1.466887.

[20] Stratmann, R. E.; Burant, J. C.; Scuseria, G. E.; Frisch, M. J., *J. Chem. Phys.* **1997**, *106*, 10175-10183, doi: 10.1063/1.474047.

[21] Zhao, Y.; Truhlar, D. G., *Theor. Chem. Acc.* **2008**, *120*, 215-241, doi: 10.1007/s00214-007-0310-x.

[22] Wachters, A. J. H., *J. Chem. Phys.* **1970**, *52*, 1033-1036, doi: 10.1063/1.1673095.

[23] Hay, P. J., *J. Chem. Phys.* **1977**, *66*, 4377-4384, doi: 10.1063/1.433731.

[24] McLean, A. D.; Chandler, G. S., *J. Chem. Phys.* **1980**, *72*, 5639-5648, doi: 10.1063/1.438980.

[25] Krishnan, R.; Binkley, J. S.; Seeger, R.; Pople, J. A., *J. Chem. Phys.* **1980**, *72*, 650-654, doi: 10.1063/1.438955.

[26] Clark, T.; Chandrasekhar, J.; Spitznagel, G. W.; von R. Schleyer, P., *J. Comput. Chem.* **1983**, *4*, 294-301.

[27] Raghavachari, K.; Trucks, G. W., *J. Chem. Phys.* **1989**, *91*, 1062-1065, doi: 10.1063/1.457230.

[28] O'Boyle, N. M.; Tenderholt, A. L.; Langner, K. M., *J. Comput. Chem.* **2008**, *29*, 839-845, doi: 10.1002/jcc.20823.

[29] Andersson, M. P.; Uvdal, P., *J. Phys. Chem. A* **2005**, *109*, 2937-2941, doi: 10.1021/jp045733a.

[30] Stratmann, R. E.; Scuseria, G. E.; Frisch, M. J., *J. Chem. Phys.* **1998**, *109*, 8218-8224, doi: 10.1063/1.477483.

[31] Bauernschmitt, R.; Ahlrichs, R., *Chem. Phys. Lett.* **1996**, *256*, 454-464, doi: 10.1016/0009-2614(96)00440-X.

[32] Casida, M. E.; Jamorski, C.; Casida, K. C.; Salahub, D. R., *J. Chem. Phys.* **1998**, *108*, 4439-4449, doi: 10.1063/1.475855.

[33] Klamt, A.; Schüürmann, G., *J. Chem. Soc., Perkin Trans. 2* **1993**, 799-805, doi: 10.1039/P29930000799.

[34] Barone, V.; Cossi, M., *J. Phys. Chem. A* **1998**, *102*, 1995-2001, doi: 10.1021/jp9716997.

[35] Cossi, M.; Rega, N.; Scalmani, G.; Barone, V., *J. Comput. Chem.* **2003**, *24*, 669-681, doi: 10.1002/jcc.10189.

[36] London, F., *J. Phys. Radium* **1937**, *8*, 397-409.

[37] McWeeny, R., *Phys. Rev.* **1962**, *126*, 1028-1034, doi: 10.1103/PhysRev.126.1028.

[38] Wolinski, K.; Hinton, J. F.; Pulay, P., *J. Am. Chem. Soc.* **1990**, *112*, 8251-8260, doi: 10.1021/ja00179a005.

[39] Cheeseman, J. R.; Trucks, G. W.; Keith, T. A.; Frisch, M. J., *J. Chem. Phys.* **1996**, *104*, 5497-5509, doi: 10.1063/1.471789.

[40] Barone, V., *Chem. Phys. Lett.* **1996**, *262*, 201-206, doi: 10.1016/0009-2614(96)01070-6.

[41] Rega, N.; Cossi, M.; Barone, V., *J. Chem. Phys.* **1996**, *105*, 11060-11067, doi: 10.1063/1.472906.

[42] Barone, V., 'Structure, Magnetic Properties and Reactivities of Open-Shell Species From Density Functional and Self-Consistent Hybrid Methods', in *Recent Advances in Density Functional Methods. Part 1* (Ed.: Chong, D. P.), World Scientific Publishing, Singapore, London, **1995**, pp. 287-334, doi: 10.1142/9789812830586_0008.

[43] Dennington, R.; Keith, T. A.; Millam, J. M. *GaussView, Version 6.1.1*; Semichem, Inc.: Shawnee Mission, KS, 2019.

[44] Chen, Z.; Wannere, C. S.; Corminboeuf, C.; Puchta, R.; von R. Schleyer, P., *Chem. Rev.* **2005**, *105*, 3842-3888, doi: 10.1021/cr030088+.

[45] Lee, C.; Yang, W.; Parr, R. G., *Phys. Rev. B* **1988**, *37*, 785-789.

[46] Becke, A. D., *J. Chem. Phys.* **1993**, *98*, 5648-5652.

[47] Koch, W.; Holthausen, M. C., *A Chemist's Guide to Density Functional Theory*, 2. ed., Wiley-VCH, Weinheim, **2002**.

[48] Miyazawa, Y.; Wang, Z.; Matsumoto, M.; Hatano, S.; Antol, I.; Kayahara, E.; Yamago, S.; Abe, M., *J. Am. Chem. Soc.* **2021**, *143*, 7426-7439, doi: 10.1021/jacs.1c01329.

[49] Wang, Z. *py.Aroma 4*; https://wongzit.github.io/program/pyaroma (accessed data, 05/2024).

[50] Geuenich, D.; Hess, K.; Köhler, F.; Herges, R., *Chem. Rev.* **2005**, *105*, 3758-3772, doi: 10.1021/cr0300901.

[51] *Persistence of Vision (Version 3.6)*; Persistence of Vision Pty. Ltd.: Williamstown, Australia, 2004. http://www.povray.org (accessed data, 07/2025).
